# Enabling microbial syringol conversion through structure-guided protein engineering

**DOI:** 10.1073/pnas.1820001116

**Published:** 2019-06-24

**Authors:** Melodie M. Machovina, Sam J. B. Mallinson, Brandon C. Knott, Alexander W. Meyers, Marc Garcia-Borràs, Lintao Bu, Japheth E. Gado, April Oliver, Graham P. Schmidt, Daniel J. Hinchen, Michael F. Crowley, Christopher W. Johnson, Ellen L. Neidle, Christina M. Payne, Kendall N. Houk, Gregg T. Beckham, John E. McGeehan, Jennifer L. DuBois

**Affiliations:** ^a^Department of Chemistry and Biochemistry, Montana State University, Bozeman, MT 59717;; ^b^Centre for Enzyme Innovation, School of Biological Sciences, Institute of Biological and Biomedical Sciences, University of Portsmouth, Portsmouth PO1 2UP, United Kingdom;; ^c^Biosciences Center, National Renewable Energy Laboratory, Golden, CO 80401;; ^d^National Bioenergy Center, National Renewable Energy Laboratory, Golden, CO 80401;; ^e^Department of Chemistry and Biochemistry, University of California, Los Angeles, CA 90095;; ^f^Department of Chemical Engineering, University of Kentucky, Lexington, KY 40506;; ^g^Department of Microbiology, University of Georgia, Athens, GA 30602;; ^h^Center for Bioenergy Innovation, Oak Ridge National Laboratory, Oak Ridge, TN 37830

**Keywords:** demethylase, P450, lignin, biorefinery

## Abstract

Microbial conversion of aromatic compounds is an emerging and promising strategy for valorization of the plant biopolymer lignin. A critical and often rate-limiting reaction in aromatic catabolism is *O*-aryl-demethylation of the abundant aromatic methoxy groups in lignin to form diols, which enables subsequent oxidative aromatic ring-opening. Recently, a cytochrome P450 system, GcoAB, was discovered to demethylate guaiacol (2-methoxyphenol), which can be produced from coniferyl alcohol-derived lignin, to form catechol. However, native GcoAB has minimal ability to demethylate syringol (2,6-dimethoxyphenol), the analogous compound that can be produced from sinapyl alcohol-derived lignin. Despite the abundance of sinapyl alcohol-based lignin in plants, no pathway for syringol catabolism has been reported to date. Here we used structure-guided protein engineering to enable microbial syringol utilization with GcoAB. Specifically, a phenylalanine residue (GcoA-F169) interferes with the binding of syringol in the active site, and on mutation to smaller amino acids, efficient syringol *O*-demethylation is achieved. Crystallography indicates that syringol adopts a productive binding pose in the variant, which molecular dynamics simulations trace to the elimination of steric clash between the highly flexible side chain of GcoA-F169 and the additional methoxy group of syringol. Finally, we demonstrate in vivo syringol turnover in *Pseudomonas putida* KT2440 with the GcoA-F169A variant. Taken together, our findings highlight the significant potential and plasticity of cytochrome P450 aromatic *O*-demethylases in the biological conversion of lignin-derived aromatic compounds.

## Significance

Lignin is an abundant but underutilized heterogeneous polymer found in terrestrial plants. In current lignocellulosic biorefinery paradigms, lignin is primarily slated for incineration, but for a nonfood plant-based bioeconomy to be successful, lignin valorization is critical. An emerging concept to valorize lignin uses aromatic–catabolic pathways and microbes to funnel heterogeneous lignin-derived aromatic compounds to single high-value products. For this approach to be viable, the discovery and engineering of enzymes to conduct key reactions is critical. In this work, we have engineered a two-component cytochrome P450 enzyme system to conduct one of the most important reactions in biological lignin conversion: aromatic O-demethylation of syringol, the base aromatic unit of S-lignin, which is highly abundant in hardwoods and grasses.

Lignin is a heterogeneous, recalcitrant biopolymer prevalent in plant cell walls, where it provides structure, defense against pathogens, and water and nutrient transport through plant tissue (1). Lignin is synthesized primarily from three aromatic building blocks (1, 2), making it the only abundant and renewable aromatic carbon feedstock available. Due to its recalcitrance, rot fungi and some bacteria have evolved powerful, oxidative enzymes that deconstruct lignin to smaller fragments (3, 4). Once broken down, the lignin oligomers can be assimilated as a carbon and energy source through at least four known aromatic-catabolic pathways (2, 5).

A critical reaction in the aerobic catabolism of lignin-derived compounds is *O*-aryl-demethylation, which occurs on methoxylated lignin-derived compounds to produce aromatic diols, such as catechol (1,2-dihydroxybenzene), protocatechuate (3,4-dihydroxybenzoate), and gallate (3,4,5-trihydroxybenzoate). Next, the aromatic rings are cleaved by intradiol or extradiol dioxygenases, and the products are funneled into central metabolism (6, 7). Harnessing this catabolic capability for transforming heterogeneous lignin streams into valuable chemicals is of keen interest (7–12) and essential for economical lignocellulose conversion (11, 13, 14).

In most plants, lignin comprises primarily coniferyl (G) and sinapyl (S) alcohol monomers, which have one methoxy group and two methoxy groups on the aryl ring, respectively. Nearly all lignin-derived aromatics require *O*-demethylation of these methoxy groups as an essential step in their conversion to central intermediates. Significant effort has been dedicated to the discovery of enzymes that can demethylate the methoxy substituents of diverse aromatic compounds (15–26). Ornston et al. (18, 19) characterized the *O*-demethylation of vanillin (4-hydroxy-3-methoxybenzaldehyde) and vanillate (4-hydroxy-3-methoxybenzoate) analogs by the VanAB monooxygenase from *Acinetobacter baylyi* ADP1, which contains a Rieske nonheme iron center. The three-component LigX monooxygenase system from *Sphingobium* sp. SYK-6, described by Masai et al. (22), also contains a Rieske nonheme iron component that is responsible for *O*-aryl-demethylation of a model biphenyl compound that mimics those in lignin. Masai et al. (20, 21) described two tetrahydrofolate-dependent enzymes, LigM and DesA, responsible for *O*-aryl-demethylation of vanillate and syringate (4-hydroxy-3,5-dimethoxybenzoate), respectively. Cytochrome P450 systems have also been reported to demethylate aromatic compounds, such as guaiacol, 4-methoxybenzoate, and guaethol (2-ethoxyphenol) (15, 17, 25, 26); however, the full gene sequences were either unreported or only recently identified (15, 17, 27), or the substrate was not of direct interest to lignin conversion (25, 26).

Our recent characterization of a two-component P450 enzyme system consisting of a reductase, GcoB, and a P450 oxidase, GcoA, demonstrated that it demethylates diverse aromatic compounds, including guaiacol (which can be derived from coniferyl alcohol and represents the aromatic functionality of G-lignin that must undergo demethylation), guaethol, anisole (methoxybenzene), 2-methylanisole, and 3-methoxycatechol (3MC) (28) with similar or greater efficiency than other *O*-aryl-demethylases described in the literature (22, 24, 29). However, GcoAB shows poor reactivity toward syringol, which can be derived from sinapyl alcohol via high-temperature reactions and represents the aromatic functionality of S-lignin that must undergo *O*-demethylation for further catabolism via ring-opening dioxygenases. G- and S-lignin are the major components of lignin in hardwoods and grasses (1). Due to their abundance, it is important to find enzymes that can act on the methoxy groups of both G- and S-lignin subunits. To date, there are no reports describing syringol *O*-demethylation or, more broadly, even its catabolism by microbes. Rather, the best-studied biological reaction of syringol is its 4–4 dimerization to form cerulignone (30–33).

Although our previous work showed that GcoA was not effective for syringol *O*-demethylation, crystallographic studies and molecular dynamics (MD) simulations indicated that a triad of active site phenylalanine residues is both highly mobile and important for positioning the substrate in its catalytically competent pose. In the present study, we hypothesized that substitution of GcoA-F169, which has the closest interaction with the bound substrate, may relax the specificity of the enzyme sufficiently to permit the *O*-demethylation of S-lignin type substrates. We tested that hypothesis using biochemical, structural, computational, and in vivo approaches. We demonstrate highly efficient in vitro and in vivo syringol turnover through structure-guided protein engineering, where the enzyme also retains highly efficient activity toward guaiacol.

## Results

### The Syringol Binding Mode Can Be Modulated by Active Site Engineering.

Guaiacol assumes a productive orientation in the active site of GcoA, resulting in a shift in the spin state of the heme iron from low (S = 1/2) to high (S = 5/2), due to the actions of amino acid side chains that create a tight-fitting hydrophobic pocket. The closest contact is with GcoA-F169, which forms a hydrophobic interaction with the C6 carbon on the aromatic ring of guaiacol. Previous MD simulations suggest that this residue is highly mobile, predicting that the productive complex forms dynamically (28). Superposition of the cocrystal structures of GcoA with guaiacol and syringol reveals a shift in the positions of GcoA-F169 and the reactive syringol methoxy group relative to the heme (see below). Functionally, these shifts in the GcoA-F169 position permit binding of syringol, although binding in the productive conformation as measured by the shift in Fe(III) spin state is substantially diminished relative to binding of guaiacol (Table 1) (28).

**Table 1.** Efficacy of GcoA-F169A relative to WT GcoA in binding and demethylating guaiacol and syringol

Parameter	WT GcoA			GcoA-F169A		
	Guaiacol	Syringol	3MC	Guaiacol	Syringol	3MC
*K*_*D*_, μM*	0.0065 ± 0.002	2.8 ± 0.5	3.7 ± 0.1	7.1 ± 0.1	1.7 ± 0.07	9.5 ± 0.2
*%*Fe(III) spin state conversion*	87 ± 1	56 ± 2	62 ± 0.01	72 ± 0.3	76 ± 0.7	65 ± 0.6
*k*_*cat*_, s^−1^^†^	6.8 ± 0.02	—	n/a^‡^	11 ± 0.03	5.9 ± 0.01	n/a
*K*_*M*_, mM^†^	60 ± 10	—	n/a	40 ± 6	10 ± 1	n/a
*k*_*cat*_*/K*_*M*_, mM^−1^s^−1^^†^	110 ± 20	—	n/a	290 ± 40	600 ± 90	n/a

* *K*_D_ was measured by titrating 0–60 µM of substrate into a solution containing 2–6 µM WT or F169A GcoA in air-saturated buffer (25 mM Hepes, 50 mM NaCl, pH 7.5, 25 °C) and recording the ferric spin state change from the low-spin (417 nm) to high-spin (388 nm) species. The % spin state conversion was calculated by dividing the final high-spin species (maximum absorbance at 388 nm) by the starting low-spin species (maximum absorbance at 417 nm).

^†^ The Michaelis constants are apparent, as the dioxygen and GcoB concentrations are not known to be saturating. The conditions used were 0.2 µM GcoAB, 100 µg/mL catalase, 300 µM NADH, 210 µM O_2_, and 0–300 µM substrate, 25 °C, 25 mM Hepes, 50 mM NaCl, pH 7.5.

^‡^ n/a and dashes: Michaelis constants for 3MC could not be directly measured because of a high level of NADH uncoupling, indicated by “n/a”; see text. A substantial amount of syringol turnover was not observed for WT GcoA, again making it impossible to measure Michaelis parameters. This is indicated by dashes.

We hypothesized that mutation of GcoA-F169 to a smaller residue (alanine) may relieve the apparent steric clash between it and the bound ligand in the active site, allowing syringol to adopt a productive conformation. Thus, we prepared the GcoA-F169A variant and measured its guaiacol and syringol binding properties (*SI Appendix*, Fig. S1). Both ligands were able to stimulate the Fe(III) spin state conversion at levels close to wild-type (WT) (Table 1), with *K*_D_ values in the low micromolar range. Notably, 3MC also bound and induced a spin state change in Fe(III), though with less affinity than guaiacol or syringol. We concluded that active site engineering could indeed lead to productive syringol binding and potentially to turnover by GcoA-F169 variants, and thus subsequently studied their reactivity with guaiacol, syringol, and 3MC.

### GcoA-F169A Efficiently Demethylates both Guaiacol and Syringol with only Limited Uncoupling.

**Scheme 1.**
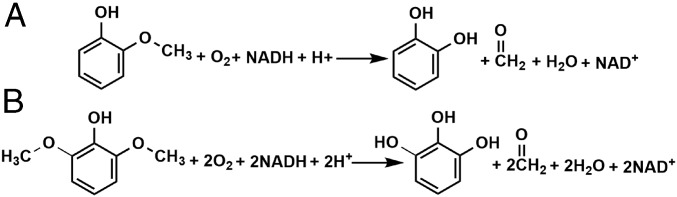
*O*-demethylation of guaiacol to form catechol and formaldehyde (*A*) and syringol to form pyrogallol and two formaldehydes (*B*). The singly demethylated species, 3MC, is expected to form as an intermediate in reaction (*B*). See Fig. 1.

Substrate analogs are known to stimulate the P450 reaction with NADH/O_2_ without concomitant oxygenation of the organic substrate. This leads to uncoupling of the NADH and substrate oxidation reactions and to reduction of O_2_ to either H_2_O_2_ or H_2_O (34). Previous work has shown that syringol stimulates NADH consumption by WT GcoA (28), although without substantial syringol turnover. To address whether guaiacol and/or syringol would serve as substrates of GcoA-F169A, we monitored the disappearance of NADH (UV/vis) and aromatic substrate (HPLC) over time (Scheme 1). The rates of organic substrate and NADH consumption were robust and similar within error regardless of whether guaiacol or syringol was used (*SI Appendix*, Table S1). This suggests that both guaiacol and syringol serve as substrates for GcoA-F169A.

**Fig. 1.**
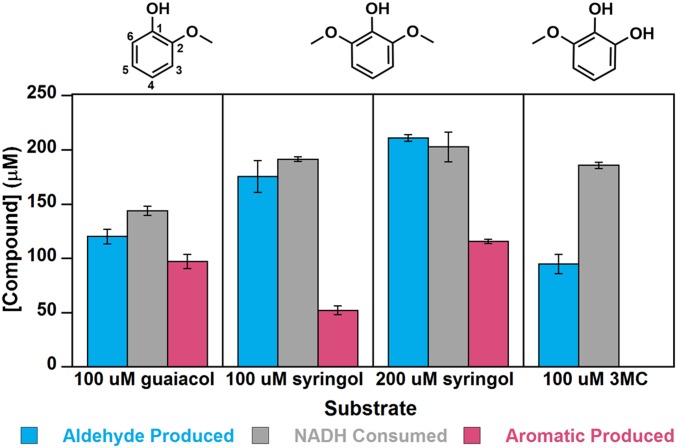
Quantitative analyses of substrate consumption and product generation indicate nearly complete coupling of NADH/O_2_ consumption to substrate *O*-demethylation for guaiacol and progressively more uncoupling for syringol and 3MC. NADH (200 µM) and guaiacol, syringol, or 3MC (100 or 200 µM) were incubated in air with 0.2 μM GcoA-F169A and GcoB (each with 25 mM Hepes, 50 mM NaCl, pH 7.5 at 25 °C and 210 µM O_2_). Reactants and products were quantified when the UV/vis spectrum ceased changing and the reaction was deemed complete. The total NADH consumed is compared with the amounts of formaldehyde and demethylated aromatic compound produced. Pyrogallol, the *O*-demethylated product of 3MC, is unstable in air under the conditions used in the assay and was not detected. Error bars represent ±1 SD from three or more independent measurements. *P* values comparing NADH consumption and formaldehyde production were 0.035 for guaiacol, 0.20 for 100 µM syringol, 0.41 for 200 µM syringol, and 0.0035 for 3MC. For NADH consumption and aromatic product production, these *P* values were 0.011 for guaiacol, 0.00031 for 100 µM syringol, and 0.0084 for 200 µM syringol.

Moreover, the oxidative *O-*demethylation of guaiacol appeared to be largely coupled to NADH consumption. When NADH and O_2_ were present in excess of guaiacol, the measured stoichiometry of the GcoA-F169A–catalyzed reaction was very close to 1 molecule each of guaiacol and NADH consumed to 1 formaldehyde and 1 catechol produced (Fig. 1), without overconsumption of NADH (103 ± 7% coupling efficiency; *SI Appendix*, Table S1).

Since both methoxy groups of syringol can potentially serve as substrates, we examined syringol turnover in several ways. Syringol (100 μM) was first incubated with NADH (200 μM) and excess dissolved O_2_ (210 μM), and the reaction with the GcoA-F169A mutant was allowed to go to completion. As with the guaiacol reaction, all the NADH and syringol were consumed (Fig. 1), implying that syringol undergoes two *O*-demethylations, producing 3MC and then pyrogallol. However, less formaldehyde (170 ± 10 μM) was produced than expected, suggesting some uncoupling of NADH/O_2_ consumption from the oxidative *O*-demethylation. Consistent with that hypothesis, 50 ± 4 μM of the singly demethylated intermediate 3MC was observed at the end of the reaction, even though sufficient NADH/O_2_ were present to enable its complete conversion to pyrogallol. Notably, pyrogallol was not detected under any of the conditions used here, possibly due to its well-known instability in the presence of O_2_ (35).

The stoichiometric analysis was next repeated with NADH and syringol present in equal concentrations (∼200 μM each; 210 μM O_2_), conditions expected to permit at most one-half of the available methoxy groups to react. All of the NADH and 150 ± 6 μM syringol were consumed, and 200 ± 3 μM formaldehyde and 120 ± 2 μM 3MC were generated (Fig. 1 and *SI Appendix*, Table S2). The accumulation of roughly one-half an equivalent of 3MC (relative to NADH) under these conditions suggested that the first *O*-demethylation of syringol must be faster than the second, and that the uncoupling reaction is likely stimulated by 3MC rather than by syringol. Consistent with those expectations, the rate of 3MC disappearance measured by HPLC was significantly slower than the disappearance of either guaiacol or syringol (2.6 ± 0.3 μM 3MC s^−1^ µmol GcoA-F169A^−1^ vs. 5.1 ± 0.8 μM syringol s^−1^ μmol GcoA-F169A^−1^; *SI Appendix*, Table S1). Moreover, the faster consumption of NADH relative to 3MC suggested diminished reaction coupling (64 ± 10% coupling; *SI Appendix*, Tables S1 and S2). In reactions containing 100 µM 3MC and 200 µM NADH, the majority of the initially available NADH was consumed, and ∼100 µM of formaldehyde was produced (Fig. 1). We hypothesized that the observed overconsumption of NADH was due to the uncoupled reaction, leading to H_2_O_2_ production. The production of H_2_O_2_ in the presence of excess NADH/O_2_ and limiting 3MC was confirmed using Amplex Red and horseradish peroxidase (HRP) (*SI Appendix*, Table S3). Consequently, accurate values for *k*_cat_ and *K*_M_ could not be measured using 3MC as a substrate (Table 1).

A broader survey of variants at the GcoA-F169 position (amino acids S, H, V, I, and L in addition to A) confirmed that GcoA-F169A exhibits the best catalytic performance in terms of both specific activity and reaction coupling, although other small side chains (S and V) also permitted reactivity with syringol, suggesting that these may permit syringol to assume a reactive conformation at the heme. Apparent steady-state kinetic parameters measured in air and at potentially subsaturating concentrations of GcoB (Table 1 and *SI Appendix*, Fig. S2 and Table S2) suggest that GcoA-F169A is a more effective catalyst toward the first methoxy group of syringol relative to guaiacol, with *k*_cat_/*K*_M_[syringol] nearly double *k*_cat_/*K*_M_[guaiacol]. Moreover, GcoA-F169A has a slightly improved performance with guaiacol as a substrate relative to the WT enzyme.

### Structural Analysis Reveals Productive Syringol Reorientation in GcoA-F169 Variants.

**Fig. 2.**
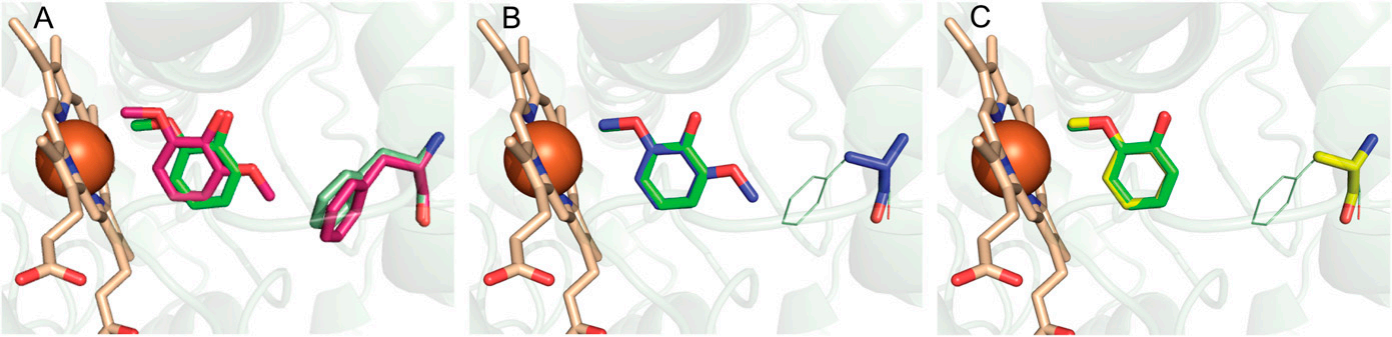
Structure-guided active site engineering of GcoA. Superpositions of WT and GcoA-F169A ligand-bound structures of GcoA, the P450 monooxygenase component of GcoAB, are shown. The heme is colored in bronze stick. (*A*) The guaiacol (green) and syringol (pink) complexes with WT GcoA are shown with the position of the GcoA-F169 residue highlighted. The translation and rotation of syringol compared with guaiacol result in a shift of the target methoxy carbon away from the heme. The Fe(III) to guaiacol methoxy carbon distance is 3.9 Å, and the Fe(III) to proximal syringol methoxy carbon distance is 4.3 Å. Data from PDB ID: 5NCB (28) and 5OMU (28). (*B*) The engineered GcoA-F169A-syringol complex (blue) enables positioning of the reactive methoxy group relative to the heme in a mode consistent with productive guaiacol binding. GcoA-F169 from the guaiacol-bound WT structure is shown in green lines. (*C*) Superposition of the WT (green) and GcoA-F169A (yellow) guaiacol-bound complexes reveals that guaiacol sits in an identical position in both crystal structures. GcoA-F169 from the guaiacol-bound WT structure is shown in green lines.

Superposition of the structures of GcoA-ligand complexes indicated a significant rotation and translation of bound syringol relative to guaiacol (Fig. 2*A*), and we hypothesized that this could form the basis for the unproductive syringol uncoupling in the native enzyme. The comparative distances between the heme and the proximal methoxy carbon of guaiacol vs. syringol are within 0.4 Å, and even closer between the heme and methoxy oxygens (within 0.1 Å). In addition, there is no significant deviation from the plane of the aromatic rings between these ligands. In contrast, the angle of presentation of the methoxy to the heme diverges significantly in these complexes. Using the angle between the methoxy oxygen, heme iron, and terminal methoxy carbon atoms [O-Fe(III)-C] as a convenient readout of relative orientation, there is a 55% increase in angle from the guaiacol-bound structure (8.3°) compared with the syringol-bound structure (12.9°).

To investigate this further, we generated multiple high-resolution GcoA-F169 variant cocrystal structures. A set of syringol-bound structures (*SI Appendix*, Table S4 and Fig. S4) provided direct insight into the minimal reduction in side chain bulk required to achieve the productive binding mode equivalent to that of guaiacol. A stepwise trajectory of the bound syringol toward this optimum orientation with decreasing side chain bulk was observed in the superposition of four cocrystal structures (*SI Appendix*, Fig. S3*A*). Specifically, GcoA-F169H creates an improved substrate orientation, further improved by GcoA-F169V, and essentially optimized in both the GcoA-F169S and -F169A proteins (*SI Appendix*, Fig. S3*A*). Indeed, a comparison of the GcoA-F169A syringol structure with the WT guaiacol structure revealed an almost perfect alignment relative to the aromatic rings of each substrate and a methoxy O-Fe-C angle of 8.6°, within 0.3° of that observed for guaiacol (Fig. 2*B*).

Each protein variant also crystallized successfully with guaiacol (*SI Appendix*, Table S5 and Fig. S5) and the structures showed that the orientation of the bound ligand remained consistent with that of the WT enzyme (*SI Appendix*, Fig. S3*B*). Even the largest reduction in side chain bulk, represented by the GcoA-F169A variant, retained the ideal reactive geometry for the natural substrate (Fig. 1*C*). Furthermore, comparison of the surrounding active site architecture confirmed no significant deviation from the WT. The resolution of these structures (1.66–2.17 Å) also provided sufficient electron density quality to explore changes in the hydration of the pocket (*SI Appendix*, Fig. S6). While the native enzyme excluded water from the active site pocket, we were interested to see whether this was maintained when a new cavity in the pocket was introduced. The syringol-bound mutants A, S, and V contain an additional ordered water in the active site (*SI Appendix*, Fig. S6), which may help maintain the substrate in a productive binding pose for catalysis. As expected, the bulkier GcoA-F169H mutant excluded water from the active site, as with the WT structure.

### Syringol Clashes with both GcoA-F169 and the Substrate Access Lid in Simulations of WT GcoA.

**Fig. 3.**
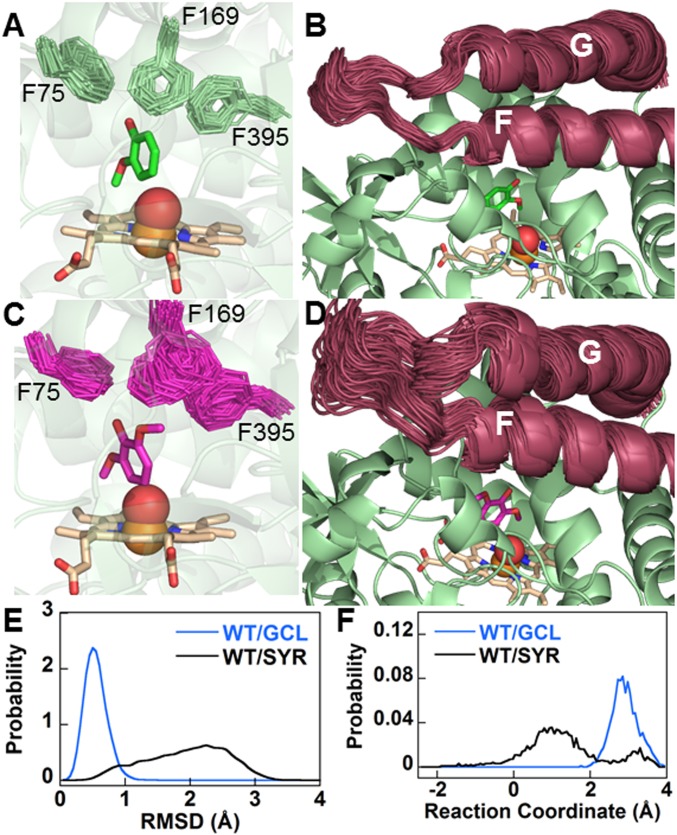
GcoA-F169 in WT GcoA and the substrate access loop are significantly displaced with bound syringol. MD simulations with bound guaiacol indicate that GcoA-F169 (*A*) and the substrate access lid (*B*) are relatively stable (Movies S1 and S3). Introducing syringol results in increased flexibility of GcoA-F169 (*C*) and the substrate access loop (*D*) (Movies S2 and S4). In *A*–*D*, the position of each of the labeled Phe side chains (or, alternatively, the substrate access loop) is shown every 4 ns over the course of the 80-ns MD simulation. Substrate, the Phe side chains, and heme are shown in sticks, and the Fe atom and the O atom of a reactive heme-oxo intermediate are shown as spheres. Probability distributions are shown for the rmsd of the six ring carbons of GcoA-F169 from their crystal structure positions (*E*) and the reaction coordinates for opening/closing of the substrate access loop (as defined in *SI Appendix*; lower values indicate more open configurations) (*F*).

In the WT enzyme with syringol bound, active site crowding can be relieved in several ways. First, as already noted, syringol can shift toward the heme (Fig. 2*A*); this effect is seen in MD simulations (80 ns) carried out on WT GcoA with either bound guaiacol or syringol, although the effect is much subtler than in the crystal structures (*SI Appendix*, Fig. S7). A second effect is more pronounced in MD simulations, which show that GcoA-F169 is significantly more flexible and perturbed from the crystal structure position when syringol rather than guaiacol is bound at the WT active site (Fig. 3 *A*, *C*, and *E*, *SI Appendix*, Fig. S8, and Movies S1 and S2). This effect is complemented by the static picture given by the crystal structures (Fig. 2*A*), showing that GcoA-F169 is “pushed away” from the substrate by a distance commensurate with the observed shift of the substrate.

Opening of the substrate access loop, a larger-scale phenomenon closely related to the movement of GcoA-F169, can also relieve active site crowding. All GcoA crystal structures reported to date present a closed active site “lid,” but previous MD simulations demonstrated the ability of the F/G helices (and their connecting loop) to move away from the active site, thus exposing the active site to solution, particularly in the apo form (28). Crystal packing may hinder lid opening in the GcoA-F169A structure; thus, the first two effects (shifting of substrate and GcoA-F169) are more pronounced in crystal structures. However, in MD of GcoA in solution, the active site loop is unconstrained, and the effect of the GcoA-F169 clash with syringol is observed less at the substrate and more on the enzyme. This includes the positioning and flexibility of GcoA-F169 (as mentioned above) and the substrate access lid (Fig. 3 *B*, *D*, and *F*; *SI Appendix*, Fig. S9; and Movies S3 and S4), which is significantly more prone to open with syringol bound in WT GcoA than with guaiacol, as well as either substrate bound in MD simulations on the GcoA-F169A mutant. Open and closed access loops have been observed in P450 enzyme [P450cam (34) and BM3 (35, 36)] crystal structures. The open GcoA configurations that we observe in MD simulations are approximately one-half as open as the aforementioned open crystal structures and possibly not sufficiently open to allow substrate ingress and egress. However, this principle observed over 80 ns is likely to be more pronounced over the course of the full catalytic cycle. We also note that to date, efforts to crystallize GcoA in the apo state have proven unsuccessful; when achieved, this may reveal a more open configuration of the substrate access lid.

The above conclusions from 80-ns MD simulations are also supported by a deeper analysis of three independent 1-µs MD trajectories of WT GcoA with syringol and guaiacol bound at the active site, which were originally presented in our previous study (figure S21 in ref. 28). GcoA-F169 is significantly perturbed from its crystal structure position and more mobile; this coincides with an increased propensity to open the substrate access lid, which is the only region of significant difference in flexibility (*SI Appendix*, Fig. S10).

We also performed density functional theory (DFT) calculations on a truncated active site model, demonstrating that the *O*-demethylation of syringol proceeds via a similar pathway as previously described for guaiacol (28) (*SI Appendix*, Fig. S11 and Table S6). Optimized transition state geometries and free energy barriers for the rate-limiting hydrogen atom transfer are likewise very similar in the two cases. In addition, replica exchange thermodynamic integration (RETI) simulations were conducted to examine relative free energies associated with substrate binding and mutating GcoA-F169 in the closed state of the enzyme (*SI Appendix*, Figs. S12 and S13). These RETI simulations revealed additional substrate binding modes, made possible by the “softer” interactions between the substrate and enzyme as the simulations gradually “turn on” and “turn off” electrostatic and van der Waals components of intermolecular interactions, and quantify the effect of this binding flexibility on binding thermodynamics.

### Sequence Position 169 in CYP255A Enzymes Is Highly Variable.

**Fig. 4.**
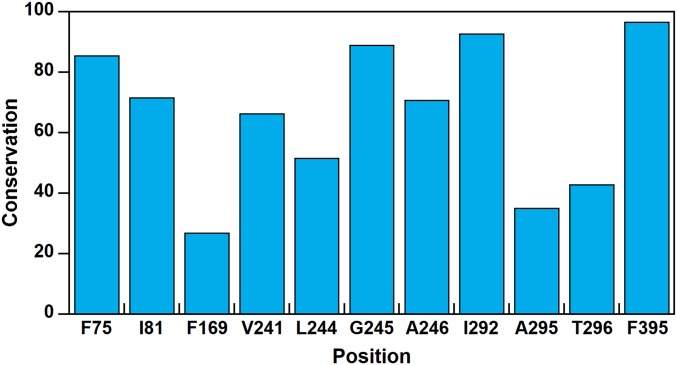
Bioinformatic analysis of CYP255A sequences indicates variability in the 169th sequence position. Conservation of residues within 6 Å of guaiacol in GcoA, determined via an analysis of protein sequences from 482 GcoA homologs. (Details provided in *SI Appendix*.) Conservation scores are reported as percentiles. F169 is less conserved than 73% of the positions in GcoA. Data from PDB ID: 5NCB (28).

GcoA belongs to the CYP255A family of cytochrome P450 enzymes (27). Conservation analyses of GcoA homologs revealed a notably variable position 169 among active site residues. Moreover, GcoA-F169 not only is the least conserved of the triad of phenylalanine residues in the active site, it is also among the least conserved positions in the entire protein (Fig. 4 and *SI Appendix*, Fig. S14 and Table S7). From a multiple sequence alignment, we determined that alanine and phenylalanine are the most frequent residues used by CYP255A enzymes at position 169, with alanine present in the majority of sequences. Thus, the GcoA-F169A mutant, which showed enhanced turnover on guaiacol and syringol, is closer to the CYP255A consensus protein than the WT. It is interesting that although none of the GcoA homologs in our analyses exhibits a histidine at position 169, the GcoA-F169H mutant was stable and showed the highest specific activity on guaiacol. Next to GcoA-F169, A295 and T296 show the greatest variability of residues in the active site. Besides these, other residues within 6 Å of the center of mass of the guaiacol substrate generally show high conservation.

### F169A Enables In Vivo Syringol Conversion by GcoA.

**Fig. 5.**
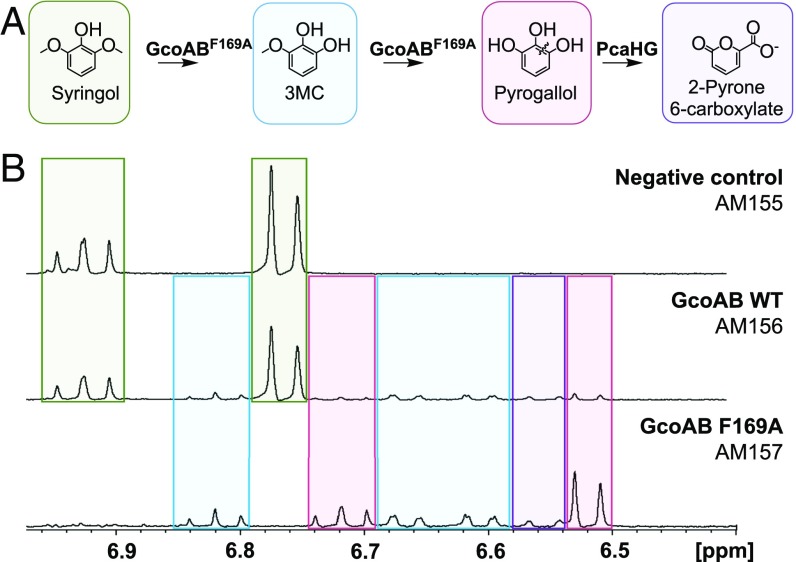
GcoA-F169A converts syringol in vivo. (*A*) A pathway for in vivo syringol *O*-demethylation to pyrogallol and cleavage to 2-pyrone 6-carboxylate is proposed. (*B*) After 6 h, strains were analyzed for their ability to turn over syringol via ^1^H NMR spectroscopy. Syringol (green) is completely converted to pyrogallol (pink), 3MC (blue), or 2-pyrone 6-carboxylate (purple) in AM157. The WT GcoA enzyme in AM156 shows only small amounts of conversion. AM155, which does not express GcoA, shows no conversion.

Finally, we aimed to demonstrate in vivo conversion of syringol to pyrogallol using *Pseudomonas putida* KT2440, which has many native aromatic-catabolic pathways relevant to lignin conversion (8, 30, 37, 38). To accomplish this, we transformed plasmids expressing WT GcoA or the GcoA-F169A variant and GcoB into a strain that constitutively overexpresses PcaHG (*SI Appendix*, Tables S8 and S9), a native 3,4-protocatechuate dioxygenase from *P. putida* that converts pyrogallol into 2-pyrone-6-carboxylate, a more stable product (Fig. 5*A*) (39). ^1^H NMR analysis of the culture medium revealed that, when cultured with 20 mM glucose and 1 mM syringol, peaks corresponding to syringol completely disappeared in cells expressing GcoA-F169A (AM157) after 6 h, which coincided with the appearance of 3MC, pyrogallol, and 2-pyrone 6-carboxylate (Fig. 5*B* and *SI Appendix*, Fig. S15). A standard for 2-pyrone 6-carboxylate was not available; however, we did verify the presence of 2-pyrone 6-carboxylate using LC-MS-MS (*SI Appendix*, Fig. S15). While small amounts of pyrogallol, 3MC, and 2-pyrone 6-carboxylate were also observed with the WT GcoAB (AM156), nearly 60% of the original syringol remained after 6 h. Neither pyrogallol nor 3MC was observed in the strain lacking GcoAB (AM155). These data indicate that the F169A variation enhances in vivo *O-*demethylation of syringol to 3MC and pyrogallol by GcoA.

## Discussion and Conclusions

The creation of enzymes that overcome the challenge of lignin heterogeneity through increased substrate promiscuity is an attractive goal, but this might come at the cost of reduced activity toward the natural substrate. Unexpectedly, we found that GcoA-F169A not only binds both guaiacol and syringol in a productive orientation analogous to guaiacol in WT GcoA (Fig. 2), but also is more catalytically efficient for *O*-demethylation of both guaiacol and syringol relative to WT, where *O*-demethylation is well coupled to NADH oxidation (Fig. 1 and Table 1). Along with this biochemical observation, the bioinformatics analysis shows that alanine is the most prevalent residue in the 169th sequence position in the CYP255A family. Considering these findings together, it is surprising that the WT GcoA does not possess an alanine at position 169 if guaiacol is the primary substrate, as was assumed in the original reports of GcoAB (17, 27, 28). Given that the GcoA-F169A mutation results in improved turnover of guaiacol, we speculate that either guaiacol is not the primary substrate or there has been little evolutionary pressure in *Amycolatopsis* sp. ATCC 39116 for improved turnover of guaiacol. Another potential explanation could be that syringol *O*-demethylation and subsequent ring cleavage lead to dead-end products that cannot be catabolized and may be toxic to the microbe; thus, GcoA-F169 could function to prevent natural syringol catabolism.

As a first step toward enabling syringol catabolism, in vivo experiments validated the in vitro studies by illustrating efficient *O*-demethylation of syringol and 3MC by the GcoA-F169 mutant. While 2-pyrone 6-carboxylate was detected, pyrogallol is a poor substrate of PcaHG, as most of the intermediate is lost to oxidation (*SI Appendix*, Fig. S15), and whether 2-pyrone-6-carboxylate can be catabolized further is unclear. Future work could focus on identifying or developing a dioxygenase capable of cleaving pyrogallol to a product that could be further metabolized. Coupled with GcoAB, the *meta*-cleavage pathway of *P. putida* mt-2 might enable complete assimilation of syringol if pyrogallol can be efficiently cleaved to 2-hydroxymuconate.

Cytochrome P450 systems represent one of the most versatile classes of enzymes, making them an ideal target for engineering enhanced activity and substrate promiscuity. Our system is a prime example. The mutation of a single residue resulted in efficient turnover of an S-lignin substrate, syringol, in addition to the native G-subunit substrate, guaiacol, which is not efficiently achieved in the WT enzyme. The plasticity of the GcoA active site may be amenable to even more modifications, allowing us to encompass other lignin monomers as substrates, such as four-substituted compounds (e.g., vanillin, syringaldehyde). Indeed, in previous work, we showed that T296 sterically clashes with the C4 position of vanillin, preventing *O*-demethylation (28). Interestingly, the 296 position is also quite variable according to the bioinformatics analysis (Fig. 4). The results described here suggest that we may use a similar structure-guided approach to investigate the activity of several T296 variants on 4-substituted compounds. As a large number of lignin degradation products contain *para*-substituted R groups that are bulkier than hydrogen, creating an engineered bacterium in which a minimal number of genetic insertions lead to maximal lignin bioconversion is of keen interest for future work. More broadly, the evolutionary trajectory, substrate specificity, and catalytic efficiency of the CYP255A family of aromatic *O*-demethylases will be the subject of future work aimed at elucidating the relevance of this cytochrome P450 family for microbial lignin conversion.

## Methods

### Protein Expression and Purification.

Mutagenesis was performed using primers listed in *SI Appendix*, with the Q5 polymerase and KLD enzyme mix (New England BioLabs) according to the manufacturer’s protocol. Proteins were expressed as described previously (28).

### Crystallization and Structure Determination.

Crystallization, diffraction experiments, and structure solution were carried out as described previously (28).

### Biochemical Characterization.

#### Heme quantification of GcoA-F169 mutants.

Catalytically active heme bound to each GcoA mutant was determined as reported previously (28, 40) and described in detail in *SI Appendix*.

#### Determination of [FAD] and nonheme [Fe] in GcoB.

The FAD and 2Fe-2S contents of GcoB were measured as reported previously (28, 41) and described in detail in *SI Appendix*.

#### Steady-state kinetics of GcoA-F169A.

The *O*-demethylation reactions of guaiacol, syringol, and 3MC were continuously monitored using the NADH consumption assay as reported previously (28) and described in detail in *SI Appendix*.

#### Determination of substrate dissociation constants (K_D_) with GcoA-F169A.

The equilibrium binding constant, *K*_*D*_, for GcoA-F169A and guaiacol, syringol, and 3MC was determined as described previously (28) and detailed in *SI Appendix*.

#### Formaldehyde determination.

The [formaldehyde] produced on reaction with GcoA-F169A and substrates was determined using a colorimetric assay with tryptophan (28, 42); details are provided in *SI Appendix*.

#### HPLC for product identification and specific activity measurement.

HPLC was used to verify the *O*-demethylated product of GcoA-F169A GcoA/GcoB with guaiacol, syringol, or 3MC. In addition, discontinuous HPLC was used to determine the specific activity of aromatic product disappearance. Details are provided in *SI Appendix*.

#### Detection of H_2_O_2_ via HRP and Amplex Red assay.

A colorimetric assay involving HRP and Amplex Red was used to quantify H_2_O_2_ in the reaction between GcoA-F169A GcoA/GcoB, NADH and guaiacol, syringol, or 3MC, as described in *SI Appendix*.

### MD, DFT, and Bioinformatics.

MD simulations and DFT calculations were performed following a similar methodology as in our previous work (28). Full details of the computational methods and references are provided in *SI Appendix*. A total of 482 homologous CYP255A sequences were retrieved from a blastp search against GcoA. After multiple sequence alignment, conservation was analyzed from relative entropy calculations for each site. Further details are provided in *SI Appendix*.

### In Vivo Syringol Utilization.

Strains used for shake flask experiments were grown overnight in LB media and resuspended the next day in M9 minimal media with 20 mM glucose, as described in *SI Appendix*. Cells were grown until they reached an OD_600_ of approximately 1, at which point syringol was added at a final concentration of 1 mM. ^1^H NMR spectroscopy was used to analyze syringol consumption.

### Data Deposition.

The atomic coordinates and structure factors have been deposited in the Protein Data Bank, https://www.wwpdb.org (PDB ID codes 6HQK, 6HQL, 6HQM, 6HQN, 6HQO, 6HQP, 6HQQ, 6HQR, 6HQS, and 6HQT).

## Supplementary Material

Supplementary File

Supplementary File

Supplementary File

Supplementary File

Supplementary File
