# Supplementary material for: Enabling microbial syringol conversion through structure-guided protein engineering
Source: Proc Natl Acad Sci U S A. 2019 Jun 24;116(28):13970–6. doi: 10.1073/pnas.1820001116 (PMC6628648; doi:10.1073/pnas.1820001116)
Supplement: Supplementary File [file pnas.1820001116.sapp.pdf]

Supplementary Information for

***Enabling microbial syringol conversion through structure-guided protein engineering***

Melodie M. Machovina[a,1], Sam J.B. Mallinson[b,1], Brandon C. Knott[c,1], Alexander W. Meyers[d,1], Marc Garcia-Borràs[e,1], Lintao Bu[c], Japheth E. Gado[d,f], April Oliver[a], Graham P. Schmidt[c], Daniel J. Hinchen[b], Michael F. Crowley[c], Christopher W. Johnson[d], Ellen L. Neidle[g], Christina M. Payne[f], Kendall N. Houk[e,*], Gregg T. Beckham[d,h*], John E. McGeehan[b,*], Jennifer L. DuBois[a,*]

[a]Department of Chemistry and Biochemistry, Montana State University, Bozeman MT, USA
[b]Centre for Enzyme Innovation, School of Biological Sciences, Institute of Biological and Biomedical Sciences, University of Portsmouth, United Kingdom
[c]Biosciences Center, National Renewable Energy Laboratory, Golden CO, USA
[d]National Bioenergy Center, National Renewable Energy Laboratory, Golden CO, USA
[e]Department of Chemistry and Biochemistry, University of California at Los Angeles, Los Angeles, CA, USA
[f]Department of Chemical Engineering, University of Kentucky, Lexington, KY, USA
[g]Department of Microbiology, University of Georgia, Athens, GA, USA
[h]Center for Bioenergy Innovation, Oak Ridge National Laboratory, Oak Ridge, TN, USA

* Email: jennifer.dubois1@montana.edu; john.mcgeehan@port.ac.uk; gregg.beckham@nrel.gov; houk@chem.ucla.edu
[1] These authors contributed equally to this work

**This PDF file includes:**

Supplementary Materials and Methods
Figs. S1 to S15
Tables S1 to S10
Captions for movies S1 to S4
DFT optimized geometries
DFT Cartesian coordinates of optimized structures
References for SI reference citations

**Other supplementary materials for this manuscript include the following:**

Movies S1 to S4



www.pnas.org/cgi/doi/10.1073/pnas.1820001116

## Supplementary Materials and Methods

### *Protein expression and purification*

Expression constructs were expressed in E. coli Rosetta™ 2 (DE3) cells (Novagen). Cells were transformed with plasmids for expression of the GcoA mutants (pGcoA-F196A, pGcoA-F196H, pGcoA-F196I, pGcoA-F196L, pGcoA-F196S, or pGcoA-F196V) and plated out lysogeny broth (LB) agar containing chloramphenicol (34 mg/L) and carbenicillin (50 mg/L). A single colony was selected and used to inoculate a 20 mL starter culture of LB. After overnight growth at 37 ºC, 250 rpm, the starter culture was inoculated into 2.5 L flasks containing 1 L of terrific broth (TB) with antibiotics. At an OD600 of 1.0, 0.2 mM Isopropyl β-D-1-thiogalactopyranoside (IPTG) was added to induce protein expression. 100 mg/L 5-aminolevulinic acid (GcoA) was added to support productive cofactor incorporation. Induction of protein expression was performed for 16-18 hr at 20 ºC with shaking at 250 rpm. Affinity purification was carried out using glutathione-sepharose 4B media (GE Lifesciences) followed by GST-tag cleavage with PreScission protease (GE Lifesciences). Anion exchange chromatography was performed with Source 30Q media (GE Lifesciences) packed into a (GE HR 16/100 Column) with a 10-40% gradient of buffers A (50 mM HEPES pH 7.5, 100 mM NaCl, 1 mM DTT) and B (50 mM HEPES pH 7.5, 1 M NaCl, 1 mM DTT) respectively. For each protein, a final gel filtration step was performed using a HiLoad S200 16/60 pg column (GE Lifesciences) in a buffer containing 25 mM HEPES pH 7.5 and 50 mM NaCl.

### *Cofactor Analyses.*

*Heme Quantification.* To determine the amount of catalytically active heme, CO gas was bubbled into a cuvette containing 1.0-2.5 µM (Pierce BCA assay) F169 GcoA mutants (A,H,S,V,I,L), made up in buffer (25 mM HEPES, 50 mM NaCl, pH 7.5) containing 1.0 mM EDTA, 20% glycerol, 0.5% sodium cholate, and 0.4% non-ionic detergent. Excess sodium dithionite (~1 mg) was added to reduce the heme iron and the peak attributed to the catalytically competent, ferrous CO-bound heme (~450 nm) gradually appeared. Several scans were taken to ensure complete binding of CO to heme. A spectrum for a control containing only dithionite-reduced GcoA was measured, and a difference spectrum computed. Absorbances at 420, 450, and 490 nm were recorded to calculate the amount of active GcoA (P450) or inactive GcoA (P420 nm) (see equations 1-3). Reported values are the average of three or more measurements.

$$(\Delta A450 - \Delta A490)/0.091 = \text{nmol of P450 per mL} \qquad (1)$$

$$[(\Delta A420 - A490)_{observed} - (A450 - A490)_{theoretical}]/0.110 = \text{nmol of cytochrome P420 per mL} \qquad (2)$$

$$\text{nmol of P450 per mL} \times (-0.041) = (\Delta A420 - A490)_{theoretical} \qquad (3)$$

Here ΔA450 and ΔA420 are the differences between the reference and sample spectra at absorbances 450 and 420 nm, respectively.

*Determination of [FAD] and non-heme [Fe] in GcoB.* FAD was released from GcoB by denaturing 200 µL of a protein (0.024 µM) solution with 5 µL saturated ammonium sulfate, pH 1.4 (7% v/v $H_2SO_4$). Precipitated protein was pelleted by centrifugation and the UV/vis spectrum of the FAD-containing supernatant was measured. The absorbance at 454 nm, $\varepsilon_{FAD}$ = 11.3 $mM^{-1}$ $cm^{-1}$, and total protein concentration determined by the BCA assay (Pierce) were used to determine [FAD] bound to GcoB. An extinction coefficient for GcoB-bound FAD was estimated via the slope of a line relating absorbance at 454 nm to [GcoB-FAD].
To determine the Fe-S content of GcoB, the protein was first denatured as described above. 50 µL of supernatant was added to 25 µL of 5% w/v sodium ascorbate to reduce the iron. 100 µL of bathophenanthroline disulfonate (0.1% w/v in dd$H_2O$) was added and the sample was incubated for 1h. The resulting Fe(II) complex was quantified via its absorbance at 535 nm ($\varepsilon_{535}$ = 22.14 $mM^{-1}$ $cm^{-1}$, determined using $FeSO_4$ standards). An extinction coefficient for GcoB-bound 2Fe-2S cluster was estimated via the slope of a line relating absorbance at 423 nm to [GcoB-2Fe-2S].

### ***Steady state kinetics and substrate dissociation constants.***

*Steady state kinetics of F169A.* 0.2 µM each of F169A GcoA and GcoB were dissolved in air-saturated buffer (25 mM HEPES, 50 mM NaCl) in a cuvette at pH 7.5, 25 °C. 100 µg/mL catalase was added to each reaction to capture any $H_2O_2$ formed during the uncoupled reaction. A saturating amount of NADH (≥ 5$K_M$, 300 µM) was added and a background rate of NADH oxidation in air (~210 µM $O_2$) recorded via continuous scanning of the UV/vis spectrum (Varian Cary 50). 20-300 µM guaiacol or syringol 2-20 mM stock dissolved in DMSO was added and the reaction was monitored via measurement of UV/vis spectra for several minutes. The initial velocity was determined by disappearance of the characteristic NADH absorbance at 340 nm ($\varepsilon_{344}$ = 6.22 $mM^{-1}$ $cm^{-1}$). A plot of $v_i$ versus [guaiacol] was fit to equation 4 to obtain $k_{cat}$, $K_M$, and $k_{cat}/K_M$. For specific activity determination, the above method was used but with saturating (300 µM) guaiacol, syringol, or 3-methoxycatechol (3MC), and in the presence of all F169 GcoA mutants (A,H,S,V,I,L). The linear portion of [NADH] vs time was fit and referenced to the amount of GcoA used (0.2 µM). Reported values are the average of ≥3 measurements and reported errors are standard deviations.

$$v_i = V_{max}[S]/(K_M + [S]) \quad (4)$$

*Determination of substrate dissociation constants ($K_D$) with F169A.* 0-60 µM of guaiacol, syringol, or 3MC in 0.5 or 1 µM aliquots, were titrated into a cuvette containing 3 µM F169A GcoA in 25 mM HEPES, 50 mM NaCl, pH 7.5. The spectrum after each substrate addition was recorded, beginning with no substrate bound. The solution reached equilibrium before the next addition. A difference spectrum was made to illustrate the shift from a low-spin aquo-heme complex to the high-spin substrate-bound complex (spectral shift from 417 nm to 388 nm). The resulting difference spectra showed a peak at 388 nm, and a trough at 417 nm. The absorbance at 388 nm ($Abs_{388\text{-}417\ nm}$) was plotted as a function of [substrate], yielding a quadratic curve that was fit to equation (5) to determine the $K_D$.

$$\Delta Abs_{obs} = \frac{\Delta Abs_{max}}{2E_t}(L_0 + E_t + K_D - \sqrt{(L_0 + E_t + K_d)^2 - 4E_t * L_0}) \quad (5)$$

Where $L_0$, $E_t$, $K_D$, and $\Delta Abs_{max}$ are the ligand concentrations, total protein (subunit) concentration, the equilibrium dissociation constant, and the maximum $Abs_{388\text{-}417\ nm}$, respectively. Reported values are the average of 2 or more measurements.

***Product analysis.***

*Formaldehyde determination.* A colorimetric assay using tryptophan can be used to quantify the amount of formaldehyde produced in F169 GcoA/B reactions with guaiacol, syringol, or 3MC. 0.2 µM each of F169 GcoA mutants and GcoB were dissolved in air-saturated buffer (25 mM HEPES, 50 mM NaCl) in a cuvette at pH 7.5, 25 °C. 100 µg/mL catalase was added to each reaction to capture any $H_2O_2$ formed during the uncoupled reaction. 200 µM NADH was added and the background rate recorded. 100 (guaiacol, syringol, or 3MC) or 200 (syringol) µM of substrate was then added and the reaction monitored until there was no more change, due to either substrate, NADH or $O_2$ depletion, whichever occurred first. 200 µL of the reaction was then quenched by adding 200 µL of a 0.1% tryptophan solution in 50% ethanol and 200 µL of 90% sulfuric acid. Upon thorough mixing, 40 µL of 1% $FeCl_3$ was added. The solution was then incubated in a heating block for 90 min at 70 °C. After cooling, the absorbance was read at 575 nm and the [formaldehyde] calculated by using $\varepsilon_{575\ nm}$ = 4.2 $mM^{-1}$ $cm^{-1}$, obtained with formaldehyde as a standard (1). A negative control included everything but the substrate and was used as a baseline.

*HPLC for product identification and specific activity measurement.* Analyte analysis of the above end-point reactions (100 µM guaiacol, syringol, or 3MC, or 200 µM syringol) was performed on an Agilent 1100 LC system (Agilent Technologies) equipped with a G1315B diode array detector (DAD). Each sample and standard was injected at a volume of 10 µL onto a Symmetry C18 column 5 µm, 4.6 x 150 mm column (Waters). The column temperature was maintained at 30 °C and the buffers used to separate the analytes of interest was 0.01% TFA in water (A)/ acetonitrile (B). The separation was carried out using a gradient program of: (A) = 99% and (B) = 1% at time $t$ = 0 min; (A) = 99% and (B) = 1% at time $t$ = 2 min, (A) = 50% and (B) = 50% at $t$ = 8 min; (A) = 1% and (B) = 99% at $t$ = 8.01 min; (A) = 99% and (B) = 1% at $t$ = 10.01 min; (A) = 99% and (B) = 1% at $t$ = 11 min. The flow rate was held constant at 1.5 mL $min^{-1}$, resulting in a run time of 11 minutes. DAD wavelengths of 210 and 325 nm were used for analysis of the analytes of interest. Standard curves were generated using 0-500 µM of guaiacol, syringol, 3MC, catechol, and pyrogallol. Integrated intensities vs [standards] were plotted and the resulting standard curves used to quantify the reactants and products.

For specific activity determination, 300 µM guaiacol, syringol, or 3MC were added from 0.1 M DMSO stocks to air saturated buffer (25 mM HEPES, 50 mM NaCl, pH 7.5), with a final volume of 1 mL. The [analyte] was measured via the above HPLC method. Then, 0.2 µM F169A/ GcoB and 100 µg/mL catalase were added. Upon addition of 300 µM NADH, the timer was started and 50 µL removed every 10 (guaiacol and syringol) or 30 (3MC) seconds. The reaction of each aliquot was immediately quenched with 12.5 µL saturated ammonium sulfate, 7% v/v $H_2SO_4$ (pH 2.0) prior to loading onto the HPLC column. [Substrate] disappearance was referenced to GcoA (0.2 µM) and fit to a linear line to determine specific activity.

*Data analysis.* The consumption of NADH and subsequent production of formaldehyde and aromatic product were measured in triplicate and the error reported as ±1 standard deviation. To determine the statistical significance between NADH consumption and the products produced, the p-value was determined for all runs containing guaiacol, syringol, and 3MC. These values were calculated using two degrees of freedom, a tail value of two, and assuming a t-statistical value for unequal variances.

***Uncoupling reactions.***

*Detection of $H_2O_2$ via horseradish peroxidase (HRP) and Amplex Red assay.* The reaction between 100 µM guaiacol or 3MC with 0.2 µM F169A/GcoB and 100 µM NADH in air-saturated buffer (25 mM HEPES, 50 mM NaCl, pH 7.5) was monitored continuously in a quartz cuvette, using the NADH consumption assay described above. The same thing was done for syringol, but with either 100 or 200 µM NADH. When there was no longer any change in the spectra, e.g., the reaction was completed, 100 µL was removed from the cuvette and pipetted into a 96-well microplate. A 5 mL solution containing 50 µL of 10 mM Amplex Red (prepared in DMSO and stored at -20 °C) and 100 µL of 10 U/mL HRP was made up in the above buffer. 100 µL of this was added to each of the wells with each reaction sample. The plate was incubated in the dark at room temperature for 30 min, at which point the absorbance at

572 nm was recorded by a Varioskan Lux microplate reader (Thermo Scientific). The absorbance was compared to a standard curve with 0-100 µM $H_2O_2$ to quantify the amount of peroxide produced in the reactions.

*Crystallography*
Purified protein was buffer exchanged into 10 mM HEPES pH 7.5 and concentrated to an A280 value of 12, as measured on a NanoDrop 2000 spectrophotometer (Thermo Fisher). Crystals of GcoA were grown with 2.4 M sodium malonate and 200 mM substrate, dissolved in 40% DMSO where necessary. Crystals were cryocooled directly in liquid N2 without further addition of cryoprotectants. All data were collected at Diamond Light Source (Harwell, UK). For each crystal, 1800 images were taken at 0.1° increments using the default wavelength of 0.9795Å on beamline i04. Data was captured on a Pilatus 6M-F detector. All phases were solved by molecular replacement from the original WT GcoA structure in complex with guaiacol with all non-polypeptide components removed. Data were processed, phased, and models were built and refined using Xia2 (2-6) and the Phenix suite (7-10).

*Strain construction*
For genomic manipulations in *P. putida* KT2440, an antibiotic-sacB counter and counterselection method was utilized as described in Blomfield at al. (11) and modified for *P. putida* KT2440 (12). Diagnostic PCR was utilized to confirm correct integrations or deletions using the 2X myTaq™ system (Bioline). Specific strain construction details are included in Table S8, plasmid construction details are included in Table S9, and oligo sequences are included in S10.

To transform cells for episomal expression in *P. putida*, cells were initially grown shaking at 225 rpm, 30 °C, overnight in Luria-Bertani (LB) medium containing 10 g/L tryptone, 5 g/L yeast extract, and 5 g/L NaCl, and used to inoculate LB the following day to an $OD_{600}$ of ~0.1 and grown until they reached an $OD_{600}$ of 0.6. Cells were then washed three times using 300 mM sucrose and resuspended in a volume 1/50 of the original culture (13). Plasmid DNA was added, and this mixture was then transferred to 1 mm electroporation cuvettes and electroporated at 1.6 kV, 25 µF, 200 Ω. Following the addition of 950 uL of SOC media (NEB), cells were shaken for an additional 45 minutes at 30°C, 220 rpm, and plated on solid media containing appropriate antibiotics.

*Shake flask experiments and in vivo product analyses*
Strains evaluated in shake flask experiments were initially grown overnight in LB media with appropriate antibiotics from glycerol stocks and resuspended the following day in M9 minimal media (6.78 g/L $Na_2HPO_4$, 3 g/L $KH_2PO_4$, 0.5 g/L NaCl, 1 g/L $NH_4Cl$, 2 mM $MgSO_4$, 100 µM $CaCl_2$, and 40 µM $FeSO_4 \cdot 7H_2O$) supplemented with 20 mM glucose (Sigma-Aldrich) and 50 mg/mL kanamycin. Cells were grown until they reached an $OD_{600}$ of 1, at which point syringol (Sigma-Aldrich) was added to a final concentration of 1 mM. Syringol concentrations were quantified using any $^1H$ NMR, and spectra were collected using a Bruker Avance III HD 400 MHz Spectrometer and analyzed using Bruker TopSpin3.7 software. For identification of unknown compounds, samples were analyzed via LC-MS-MS using the Acquity UPLC system (Waters Inc.). Compounds were separated using a C18 (evo) column (Kinetex) with the mobile phase comprised of a water, acetonitrile, and .1% formate. Flow was directly analyzed by SYNAPT HD-MS using electron spray ionization (ESI) in negative ion mode.

*Bioinformatics Analysis*
CYP255A sequences were retrieved by a blastp search against the non-redundant protein sequence database (14) using GcoA as the query sequence. From the blast results, only sequences with a query cover of at least 66% and sharing a sequence identity of greater than 28% but less than 97% with the query sequence were retained. In total, 482 homologs of GcoA were retrieved. A multiple sequence alignment (MSA) was carried out using MAFFT (15) with Biopython (16). Conservation analysis was implemented by computing the relative entropy for each site in the MSA (17). The relative entropy is given by equation (6):

$$\mathrm{R.E} = \sum_{i=1}^{20} (\mathrm{p_i} \log \frac{\mathrm{p_i}}{\mathrm{p_i^{MSA}}}) \quad (6)$$

where $p_i$ is the frequency of amino acid i in the given site and $\mathrm{p_i^{MSA}}$ is the frequency of amino acid i in the MSA.

*Molecular dynamics (MD) simulations*
*Unrestrained MD simulations.* Unrestrained MD simulations were performed on both WT GcoA and F169A mutant with either guaiacol or syringol bound at the active site. The previous constructed model of WT GcoA with guaiacol bound at the active site was used as the initial conformation to generate these structures (1). The heme group was kept in a hexacoordinate state (Compound I). Similar to previous work, five histidine residues, including His131, His221, His224, His255, and His343 were doubly protonated (+1 charge), while the remaining histidine residues were kept neutral (singly protonated). $Na^+$ cations were added to each system to achieve charge neutrality, resulting in ~74,000 atoms for each system.

The ff14SB Amber force field (18) parameters were used for the enzyme and Generalized Amber Force Field (GAFF) (19, 20) parameters were used for the various substrates, as reported previously (1). Force field parameters for the heme group were taken from ref (21). Particle mesh Ewald (PME) (22) was used for long-range interactions and the cutoff distance for nonbonded interactions was 9 Å. All the simulations were conducted using NAMD program(23) with a time-step of 2.0 fs. SHAKE algorithm (24) was used to keep bonds to hydrogen atoms fixed. Langevin thermostat with a collision frequency of 1.0 $ps^{-1}$ was used to keep the temperature at 300 K. Each system was relaxed in NPT ensemble for 500 ps first, followed by 80 ns of production was performed in the NVT ensemble. For the initial NPT dynamics, the pressure was held at 1 atm using a Nosé− Hoover barostat coupled to a Langevin piston (25, 26) with a damping time of 100 fs and a period of 200 fs.

As in our previous work (1) we employed the following reaction coordinate to describe the opening/closing motion of GcoA: $\xi = RMSD_{open} - RMSD_{closed}$, where $RMSD_{open}$ is the RMSD relative to the most open structure of WT GcoA/apo obtained via microsecond MD trajectory, and $RMSD_{closed}$ is relative to the closed crystal structure. The Cα atoms of residues 5:35 and 154:210 were chosen to calculate the RMSD.

*Replica exchange thermodynamic integration (RETI) simulations.* In order to assess relative free energies of binding in the active site of GcoA, RETI simulations were performed (27, 28) using NAMD 2.12 (29). Simulations were performed in the NPT ensemble with simulation parameters identical to those of the MD simulations described above, unless noted otherwise. Initial configurations for RETI were produced from the crystal structure appropriate for each system along with modified AMBER prmtop files to accommodate the dual-topology paradigm employed by NAMD for alchemical simulations. Each TI replica was simulated for between 150 -250 ns until convergence was achieved. The temperature was maintained at 308 K through Langevin dynamics using a collision frequency of 1.0 $ps^{-1}$. The cutoff distance for nonbonded interactions was 12.0 Å.

Two separate types of alchemical transitions were performed, with transformations targeting either the substrate or the enzyme. In each case, the transition parameter λ describes the progress of alchemical transformation. In the first kind, a syringol molecule at $\lambda = 0$ undergoes an alchemical transition to a guaiacol molecule at $\lambda = 1$. This simulation was performed with the substrate molecule in three different contexts: in a periodic water box, in the active site of solvated WT GcoA, and in the active site of solvated GcoA F169A mutant. In the second group of transformations, residue 169 of the GcoA is transformed from Phe at $\lambda = 0$ to Ala at $\lambda = 1$. This transformation was also performed in three separate contexts: with guaiacol at the active site, with syringol at the active site, and with empty active site (apo). Although these alchemical transitions were made between the entire substrate molecule (in the first set) or protein residue (in the second set), in both cases many atoms are shared in common, so zero-length 10 kcal/mol/$Å^2$ "bonds" were applied between equivalent heavy atoms, "pinning" them together. These additional restraints eliminate sampling of unphysical conformations that can slow the convergence of the calculations (30-32).

The RETI simulations were performed with 20 windows at $\lambda \in$ {0.00, 0.03, 0.06, 0.10, 0.15, 0.22, 0.29, 0.36, 0.43, 0.5, 0.56, 0.62, 0.68, 0.74, 0.80, 0.86, 0.90, 0.94, 0.97, 1.00}. Smaller increments in λ were used near the end points to avoid "end-point catastrophes" (33). The van der Waals contributions of both disappearing and appearing residues were simultaneously varied with the reaction coordinate λ. Electrostatic contributions of disappearing residues were turned off over the first half of the reaction coordinate, while those of the appearing residues were turned on over the second half of the reaction coordinate, such that both appearing and disappearing atoms were uncharged at $\lambda = 0.5$. Exchanges between adjacent replicas were attempted every 2 ps between alternating replica pairs, yielding an overall exchange attempt rate of once per 4 ps, consistent with literature guidelines (34). The combination of these parameters yielded an acceptance ratio for exchanges of approximately 50%. In each case, the final 100 ns were used to compute each ΔG reported in Figure S11. Third order spline interpolation was used to integrate the average $dU/d\lambda$ obtained from simulations at each window over the final 100 ns. Error estimates were obtained using the methodology detailed in Steinbrecher et al (35). To avoid underestimating noise error due to autocorrelations of the $dU/d\lambda$ timeseries, means and standard errors were computed by sampling all of the $dU/d\lambda$ data at a rate higher than the output rate. For each simulation, this rate was set by finding the longest triple e-folding time of the $dU/d\lambda$ autocorrelation, as determined by an exponential fit. Typical values of this time were 3-4 ps.

### *DFT calculations*

Density Functional Theory (DFT) calculations were performed using Gaussian 09 (36). A truncated model containing the porphyrin pyrrole core, Fe center and a methanethiol to mimic cysteine as Fe-axial ligand was used. Geometry optimizations and frequency calculations were performed using unrestricted B3LYP (UB3LYP) (37-39) with the LANL2DZ basis set for iron and 6-31G(d) on all other atoms. Transition states had one negative force constant corresponding to the desired transformation. Enthalpies and entropies were calculated for 1 atm and 298.15 K. A correction to the harmonic oscillator approximation, as discussed by Truhlar and co-workers, was also applied to the entropy calculations by raising all frequencies below 100 $cm^{-1}$ to 100 $cm^{-1}$ (40, 41). Single point energy calculations were performed using the dispersion-corrected functional (U)B3LYP-D3(BJ) (42, 43) with the LANL2DZ basis set on iron and 6-311+G(d,p) on all other atoms, within the CPCM polarizable conductor model (diethyl ether, $\varepsilon = 4$) (44, 45) to have an estimation of the dielectric permittivity in the enzyme active site. The use of a dielectric constant $\varepsilon$=4 has been proved

to be a good and general model to account for electronic polarization and small backbone fluctuations in enzyme active sites (46, 47). All stationary points were verified as minima or first-order saddle points by a vibrational frequency analysis. Computed structures are illustrated with CYLView (48). DFT optimized geometries and DFT Cartesian coordinates of optimized structures are present at the end of this document.

Supplementary Figures

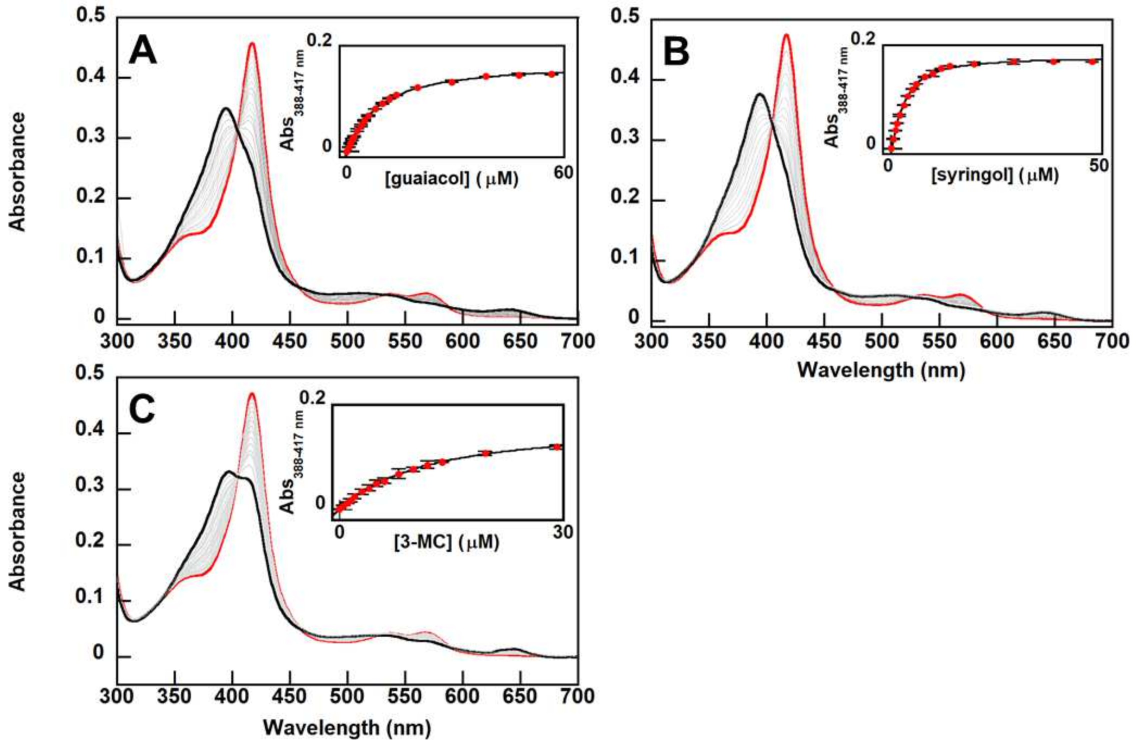


**Figure S1. Binding of both guaiacol and syringol shows a spin state change in GcoA-F169A and comparable $K_D$s.** Guaiacol (A), syringol (B), and 3MC (C) caused the Soret peak (λ = 417 nm) of GcoA-F169A to shift to 387 nm, indicating the conversion of a low-spin (red trace) to a high-spin (black trace) species. 0-60 µM substrate was titrated into a solution containing 3 µM GcoA-F169A and air-saturated buffer (25 mM HEPES, 50 mM NaCl, pH 7.5, 25 °C) and the spectra monitored until there was no more change, indicating saturation. The solution reached equilibrium prior to each substrate addition. $Abs_{388-417\ nm}$ was plotted against [substrate] and fit to the quadratic equation for weakly binding ligands (see *SI Appendix,* Methods) to obtain values for $K_D$: guaiacol = 7.1 ± 0.1 µM, syringol = 1.7 ± 0.07 µM, and 3MC = 9.5 ± 0.02 µM. Error bars represent ±1 standard deviation of three or more runs.

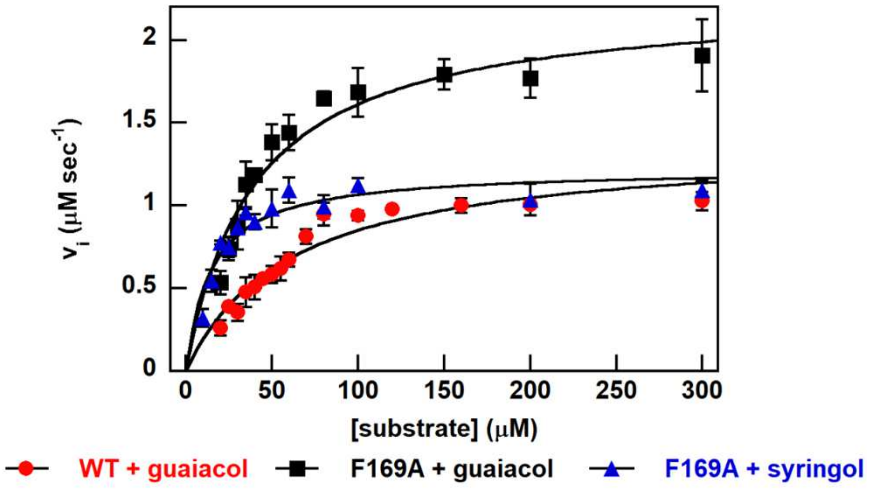


**Figure S2. GcoA-F169A demethylation of both guaiacol and syringol occurs as or more efficiently than with WT GcoA.** Initial rate of NADH consumption is plotted with either GcoA-F169A or WT GcoA as catalyst (0.2 µM) and either guaiacol or syringol as the substrate (300 µM NADH, 100 µg catalase, 210 µM $O_2$, 25 mM HEPES, 50 mM NaCl, pH 7.5, 25 °C). The data were fit to the Michaelis Menton equation. Error bars represent ±1 standard deviation of three or more runs. WT GcoA is unable to demethylate syringol; hence, only guaiacol data are shown.

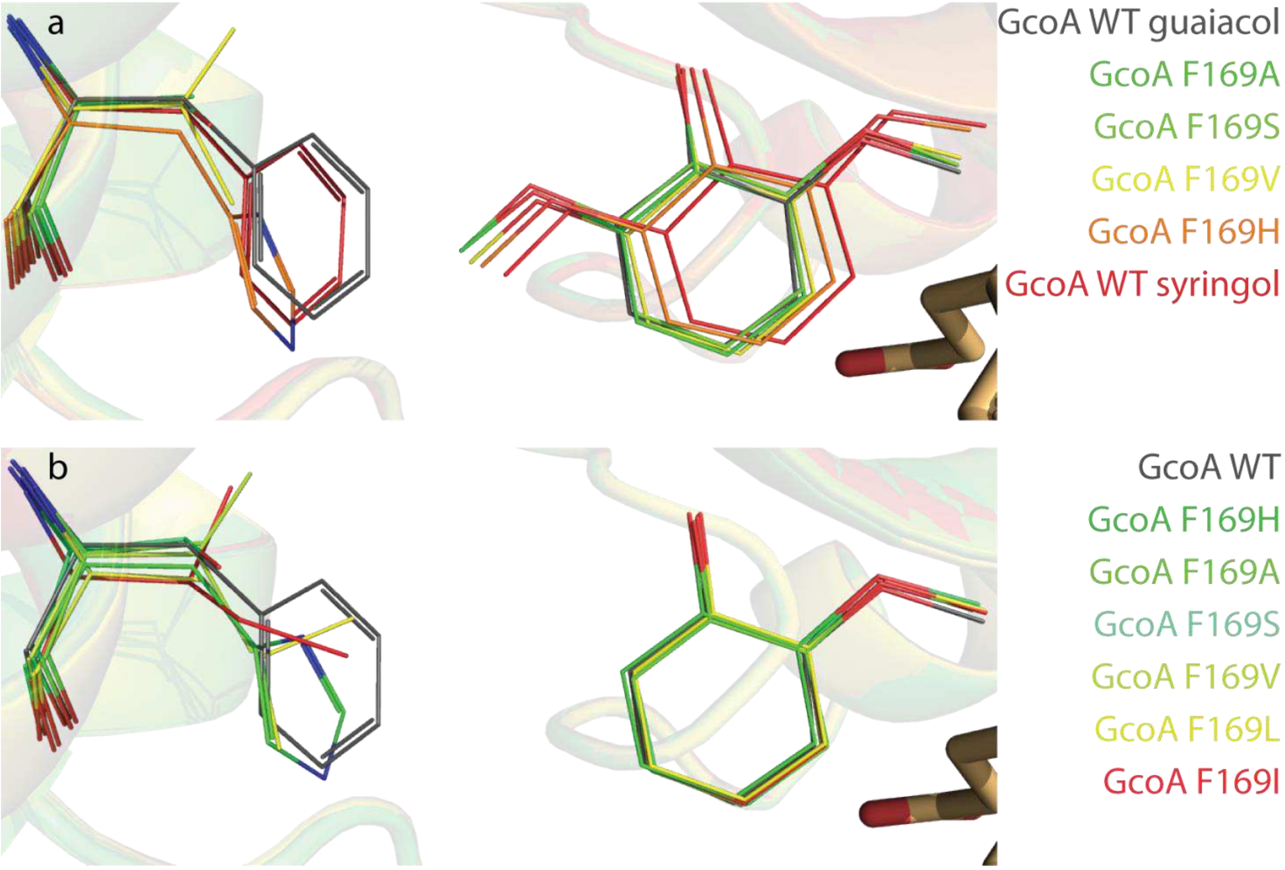


**Figure S3. Crystallographic analysis for optimal orientation of syringol and guaiacol in multiple complex structures.** Superposition of ten ligand-bound mutant structures with the previously characterized WT provided a basis for the determination of the minimum amino acid change required at position 169 for the optimum orientation of syringol, while maintaining a stable environment for guaiacol. The WT position of residue GcoA-F169 is shown in gray in both figures for reference. (A) The syringol molecules in each of the five structures shown are colored on a scale from the unproductive position of the WT in red, improved (orange and yellow), through to the optimum orientation (green) as judged by specific activity (*SI Appendix*, Table S4). (B) A superimposition of a total of six alternative mutant structures (GcoA-F169H, A, S, V, L, or I) with the WT indicated that there is no significant change from optimum guaiacol orientation induced by modification at this position.

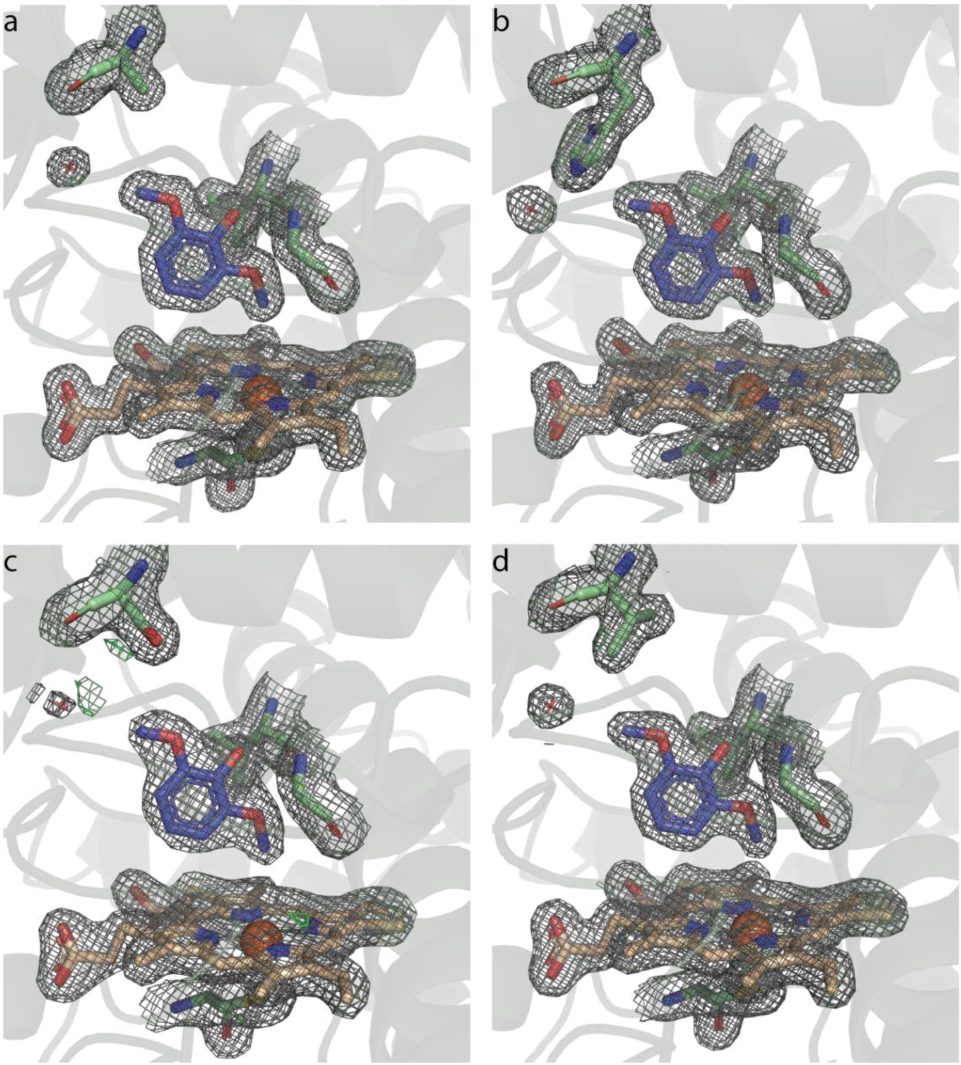


**Figure S4. Electron density of the active site of** GcoA-**F169 mutants bound to syringol.** Panels (A) – (D) show the structures of syringol bound to GcoA-F169A, H, S and V, respectively.

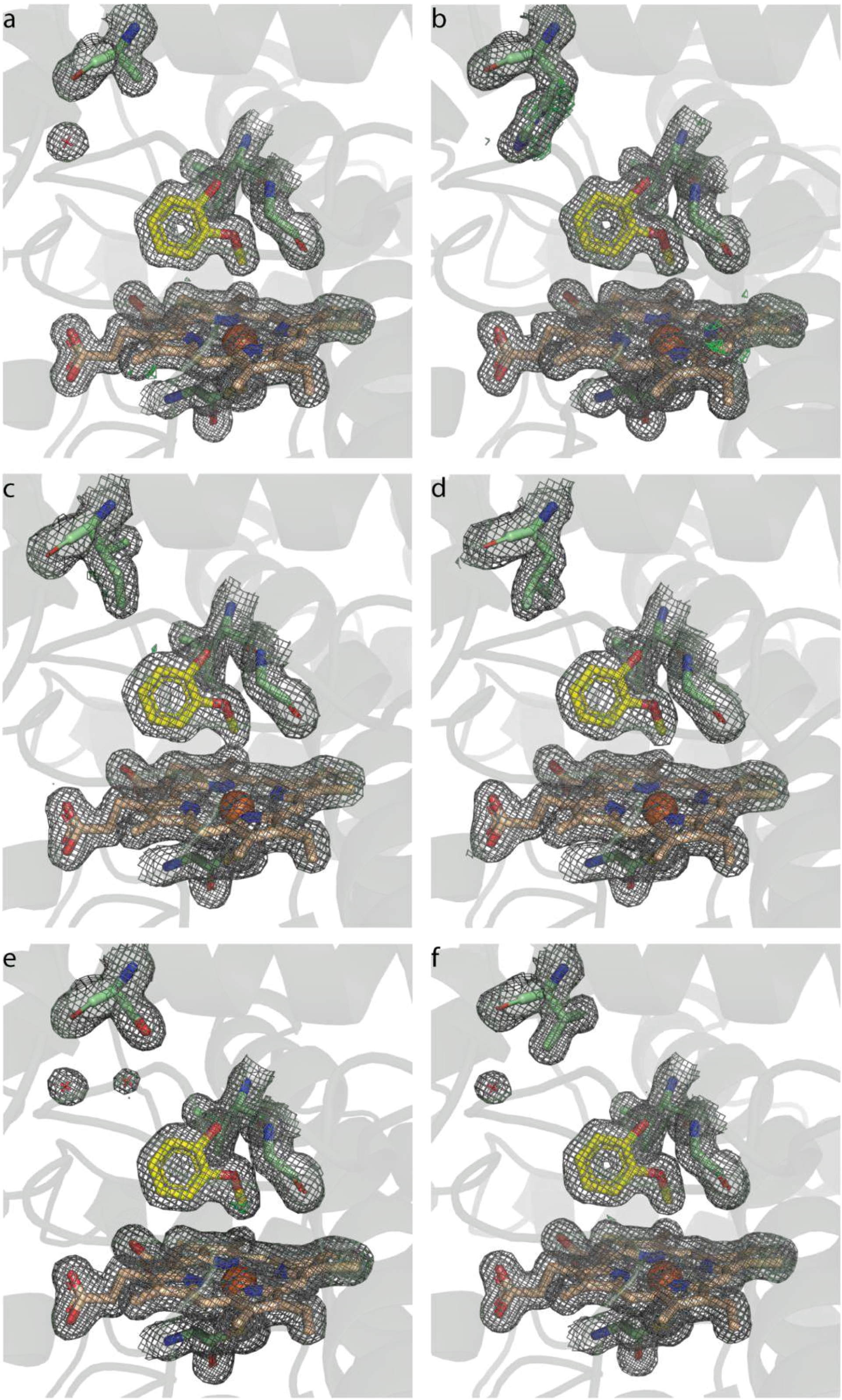


**Figure S5. Electron density of the active site of GcoA-F169 mutants bound to guaiacol.** Panels (A) – (F) show the structures of guaiacol bound to GcoA-F169A, H, I, L, S and V, respectively.

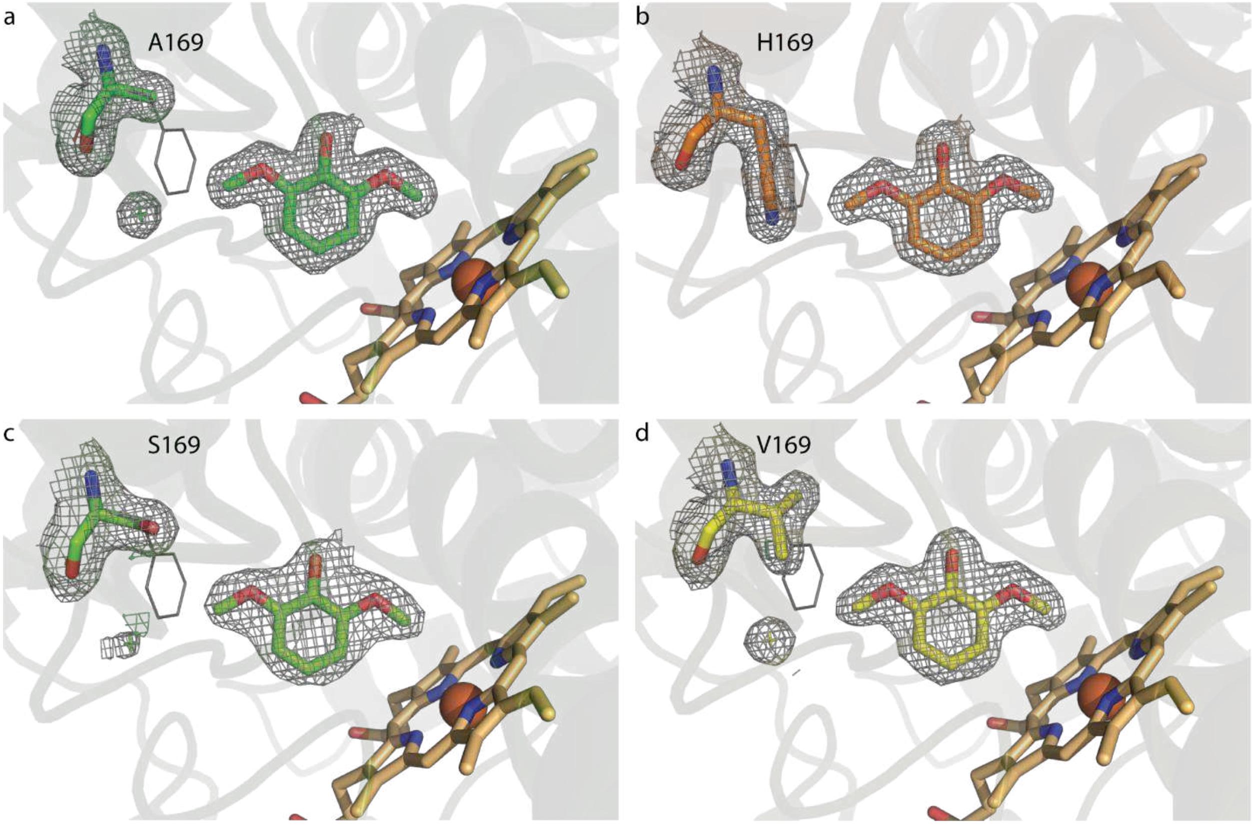


**Figure S6. Positioning of waters in the active sites of GcoA mutants with syringol.** The active sites of three of the syringol bound mutants (GcoA-F169A, S, and V – panels a, c, and d respectively) contain an extra ordered water molecule in the active site. GcoA-F169H does not (panel b). GcoA-F169A bound to syringol additionally contains another water molecule, but it has weak electron density, indicating low occupancy and/or a degree of disorder (not shown). We hypothesize that these ordered water molecules occupy space in the active site that helps to maintain the substrate in a productive binding pose.

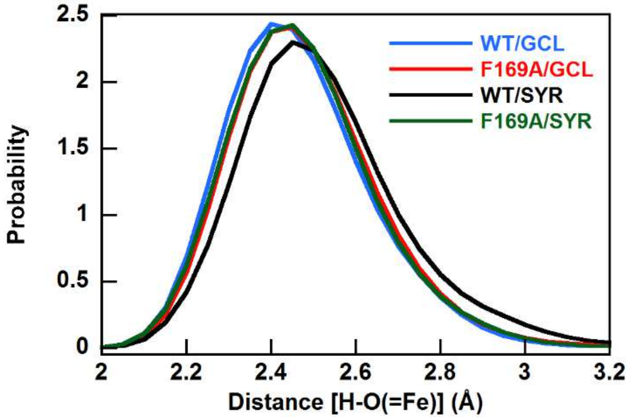


| GcoA variant | guaiacol | syringol |
|---|---|---|
| WT | 2.44 ± 0.003 Å | 2.50 ± 0.003 Å |
| F169A | 2.46 ± 0.003 Å | 2.45 ± 0.003 Å |

Figure S7. MD simulations indicate that WT GcoA bound with syringol lengthens the key distance for the rate-limiting step for demethylation, namely the distance between the methyl hydrogen and the oxygen atom bound by Compound I heme. The GcoA-F169A mutation brings this distance back to that seen with guaiacol, promoting demethylation. It should be noted that this shift as displayed in MD simulations is significantly subtler than the substrate shift seen in crystal structures (Figure 1A of main text). Error bars quoted in the table represent the standard error of the mean.

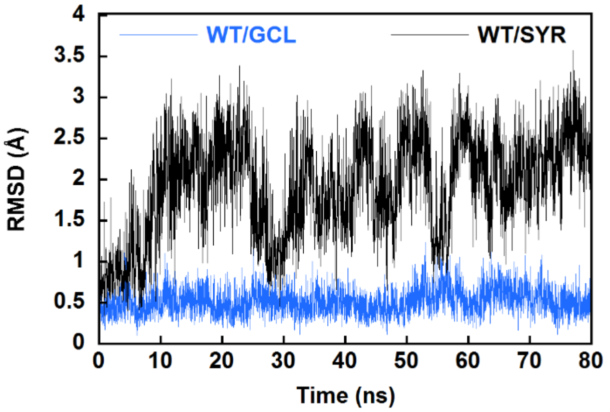


Figure S8. MD simulations indicate that introducing syringol into the active site of WT GcoA (rather than guaiacol) results in the displacement of GcoA-F169. Shown is the RMSD of the six ring carbons of GcoA-F169 from their crystal structure positions over the course of 80 ns MD simulation.

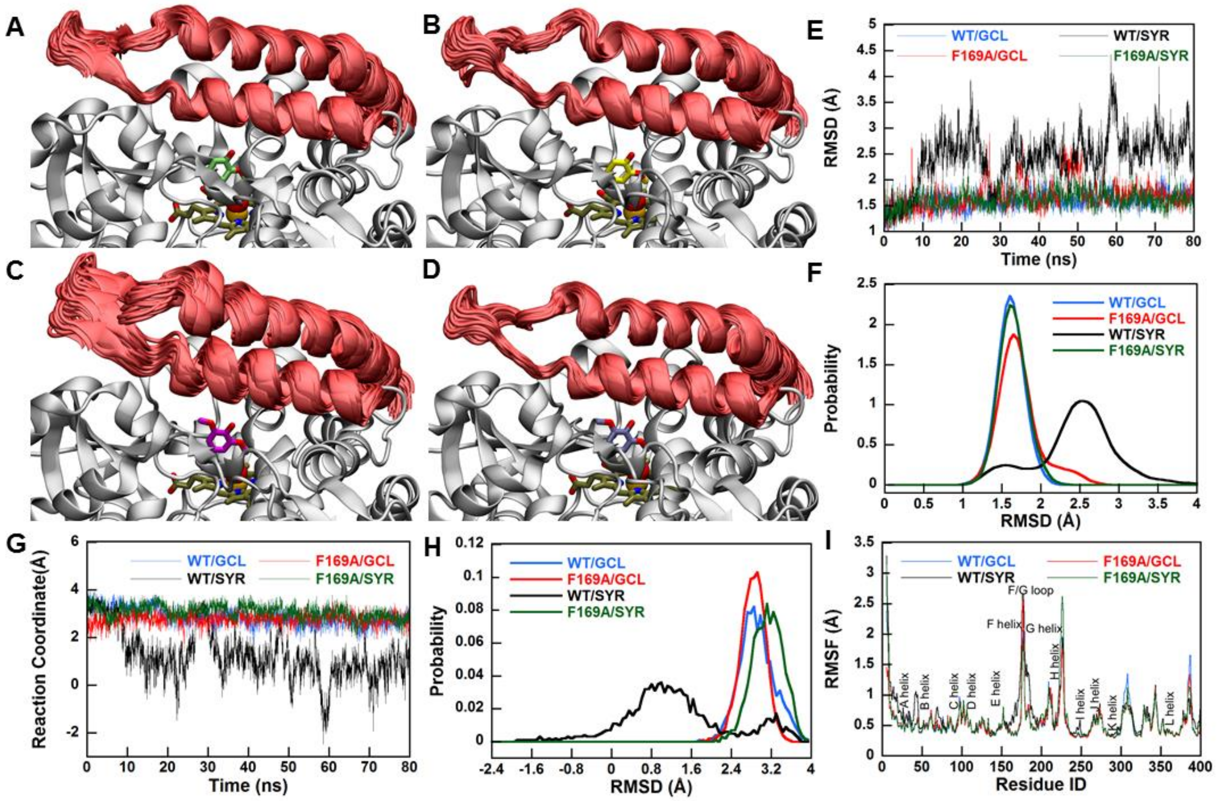


| | WT/guaiacol | F169A/guaiacol | WT/syringol | F169A/syringol |
|---|---|---|---|---|
| F169 / A169 ($C_\alpha$) | 0.33 | 0.37 | 0.67 | 0.36 |
| F169 (sidechain) | 0.41 | n/a | 0.98 | n/a |
| F75, F169 / A169, F395 ($C_\alpha$) | 0.39 | 0.37 | 0.53 | 0.37 |
| Ligand | 0.44 | 0.43 | 0.57 | 0.47 |
| Heme | 0.39 | 0.43 | 0.45 | 0.41 |
| Loop/helix F/G (154:210) ($C_\alpha$) | 0.62 | 0.66 | 0.89 | 0.62 |

Figure S9. MD simulations indicate that the substrate access loop is displaced and more flexible when syringol is bound in the WT enzyme. A) WT GcoA with guaiacol bound, B) GcoA-F169A GcoA with guaiacol bound, C) WT GcoA with syringol, and D) GcoA-F169A GcoA with syringol bound. Shown in "sticks" are the substrate and heme; heme Fe and Fe-bound oxygen are shown as spheres. The substrate access loop (residues 154:210) is shown in "cartoon" representation every 2 ns over the course of the 80 ns MD simulation. E) RMSD of all heavy atoms of the substrate access loop, as compared to the crystal structure position and F) probability distribution of the same. G) Time trace of the reaction coordinate for opening and closing of the substrate access loop (negative values indicate a more open architecture) and H) probability distribution of the same. I) RMSF (root mean square fluctuation) of GcoA (alpha carbons only) over the course of each 80 ns MS simulation. The greatest area of difference is seen in the substrate access loop region, with the fluctuations in WT GcoA with syringol bound are seen to be the greatest. The table highlights the RMSF (Å) of selected residues and regions of GcoA, as well as ligands. In this table, the RMSF calculation for "Ligand" and "Heme" is performed on all heavy atoms of those molecules (i.e. no hydrogen atoms). For the entries related to the GcoA enzyme, the RMSF calculation is performed on the alpha carbons only, with one exception: the line for "F169 (sidechain)", the RMSF calculation is for all heavy sidechain atoms.

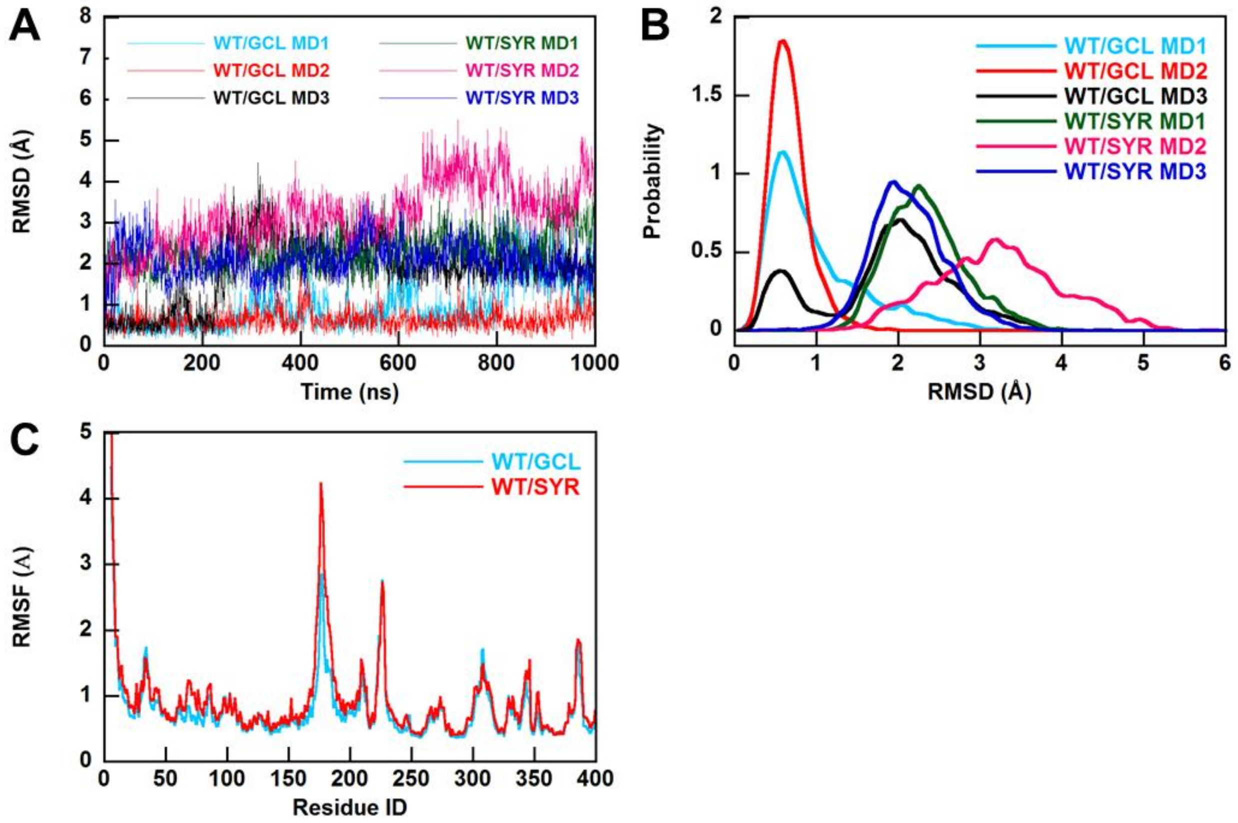


| | WT/guaiacol | | | WT/syringol | | |
|---|---|---|---|---|---|---|
| | MD1 | MD2 | MD3 | MD1 | MD2 | MD3 |
| F169 (sidechain) | 0.84 | 0.54 | 1.18 | 0.91 | 1.83 | 0.78 |
| F75, F169, F395 ($C_\alpha$) | 0.54 | 0.44 | 0.71 | 0.62 | 1.12 | 0.58 |
| Ligand | 0.56 | 0.53 | 1.60 | 0.64 | 1.79 | 0.58 |
| Heme | 0.53 | 0.50 | 0.56 | 0.58 | 0.69 | 0.53 |
| Loop/helix F/G (154:210) ($C_\alpha$) | 0.99 | 0.77 | 1.21 | 1.37 | 1.66 | 1.05 |

Figure S10. Microsecond MD simulations indicate the increased flexibility and displacement of F169 in WT GcoA when syringol is bound rather than guaiacol. These analyses are performed on three simulations (each) of WT GcoA with guaiacol and with syringol. These simulations were performed in our original study of GcoA (1); however our previous publication focused on the utilization of guaiacol and presented the results with syringol only very briefly (the fifth entry of Figure S21 in the Supplementary Information). Time traces of the reaction coordinate (RC) for opening and closing as well as the RMSD of the three active site Phe residues were presented in the SI of the prior publication. A) Time traces of the RMSD of residue F169 and B) probability distributions of the same indicate that F169 deviates substantially from its crystal structure position when syringol is bound. The movement of F169 is somewhat correlated with the motion of the substrate access loop. The probability distribution shows that each guaiacol simulation spends significant time with F169 very near the crystal structure position (first peak) whereas none of the syringol simulations display this peak. C) RMSF averaged over three simulations each for WT GcoA with either guaiacol or syringol. The significant area of deviation is in the substrate access loop (which includes F169), indicating greater dynamic flexibility when syringol is bound. Table presents RMSF (Å) of selected residues and regions of GcoA, as well as ligands. In this table, the RMSF calculation for "Ligand" and "Heme" is performed on all heavy atoms of those molecules (i.e. no hydrogen atoms). For the entries related to the GcoA enzyme, the RMSF calculation is performed on the alpha carbons only, with one exception: the line for "F169 (sidechain)", the RMSF calculation is for all heavy sidechain atoms.

P450cam has been captured in both open (e.g. PDB codes 3L61, 3L62) and closed (e.g. 3L63) states (49). Lee *et al.* note that the RMSD between the closed and open conformation is approximately 4-5 Å for the F helix and G helix and peaked around 8.5 Å at the F/G loop (Figure 2 in that publication). In our 80 ns MD simulations, the RMSD of the combined F and G helices, as well as the adjoining loop fluctuate around an RMSD value of 3 Å, whereas the adjoining loop alone fluctuates around 5 Å, as shown in the graphs here. Thus, the degree of opening by this metric is on the same order, but lower by about 50% from that observed in P450cam crystal structures.

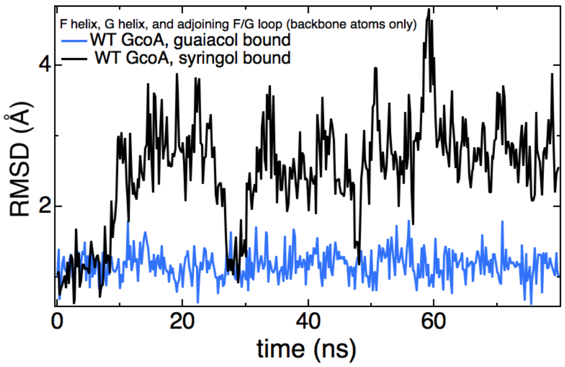


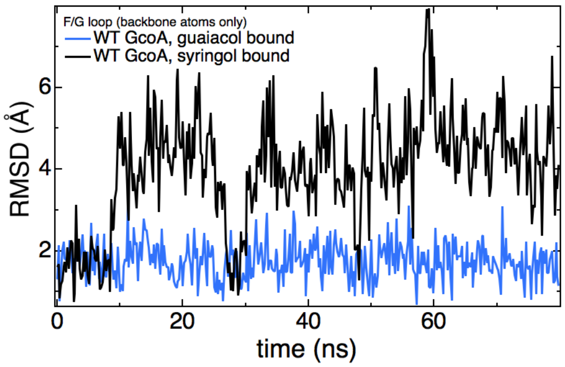


In addition to P450cam, we also note P450 BM3, which also displays crystallographic evidence of open (e.g. PDB codes 1BVY (50)) and closed (e.g. 1JPZ (51) and 1FAG (52)) structures. Dubey *et al.* presented MD simulations of BM3 in which the opening/closing of access loops was shown to be regulated by substrate binding (53). Below, we show an aligned structural comparison of GcoA, P450cam, and BM3 in their closed states.

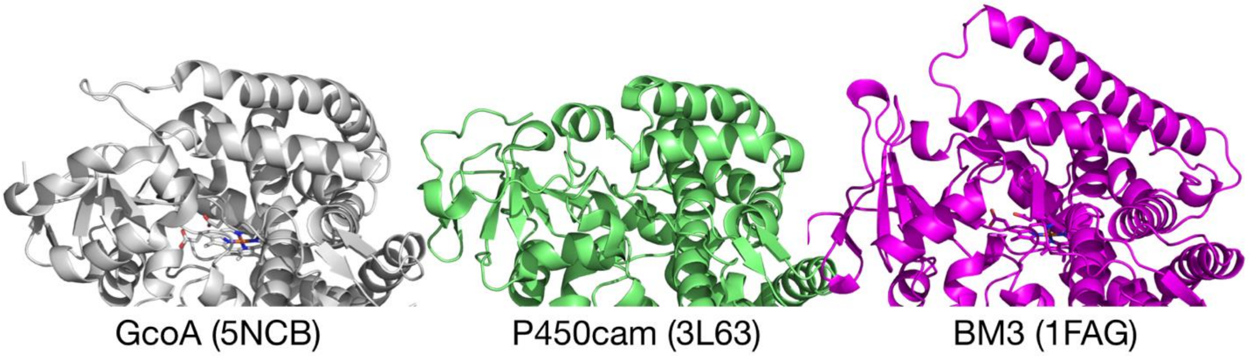


Dubey *et al*. utilize a single distance from alpha carbons to describe the opening motion of BM3 in their simulations (this distance involves a loop that is opposite of the F/G loop). Follmer *et al*. also present residue-residue distance as a metric for channel opening in P450cam (54). In order to compare our degree of openness for GcoA with those seen in the crystal structures of open/closed forms of P450cam and BM3, we have chosen a metric for each case that stretches from the F/G loop to a loop that is across the substrate access channel. For BM3, this metric is the distance from the alpha carbon of Pro45 to that of Pro196, which ranges from 19.5 Å in the closed (1JPZ) to 26.9 Å in the open state (1BVY), giving a difference of 7.4 Å. For P450cam, an analogous distance is from the alpha carbon of Asn59 to that of Ser190, which ranges from 17.3 Å in the closed structure (3L63) to 25.5 Å in the open structure (3L61), giving a difference of 8.2 Å. For GcoA, a similar metric is the distance between alpha carbons of Gly44 and Gly178, which is 6.35 Å in the closed state (5NCB). In our simulations of WT GcoA, the difference between the simulation with syringol bound and that with guaiacol bound is slightly more than 3 Å. As shown in the graph below, the average distance over the final 40 ns of the simulation is 8.0 Å (guaiacol) and 11.1 Å (syringol).

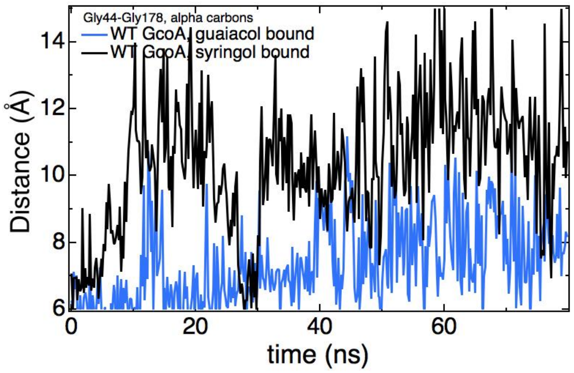


We note that, to date, efforts to crystallize GcoA in the apo state have proven unsuccessful. As a result, all existing crystal structures demonstrate GcoA in the closed state. Thus, the range of motion of the substrate access lid for GcoA is currently unverified experimentally. The range of motion that we observe in our 80 ns simulations is not necessarily the full range of motion in the catalytic cycle of GcoA (and very likely is not). Additionally, it is possible that the primary effect of the increased flexibility and propensity for the substrate access lid to open when syringol is bound could be upon substrate binding/egress or on the catalytic demethylation step.

In our 80 ns MD simulations, we find that the substrate access loop displays opening (whether by an RMSD metric or cross-channel distance) within the first 10 ns and remains open for the remaining 70 ns, with only one very brief excursion to the closed state around 30 ns. Focusing on the F/G loop backbone atoms and taking RMSD=3.0 Å as the cutoff between the open and closed states, the simulation with syringol is in the open state for 81% of the simulation, whereas the guaiacol-bound simulation is open for less than 1% of the simulation time. (We note again, that this definition of “open” does not signify that this is the most open state of GcoA).

A)

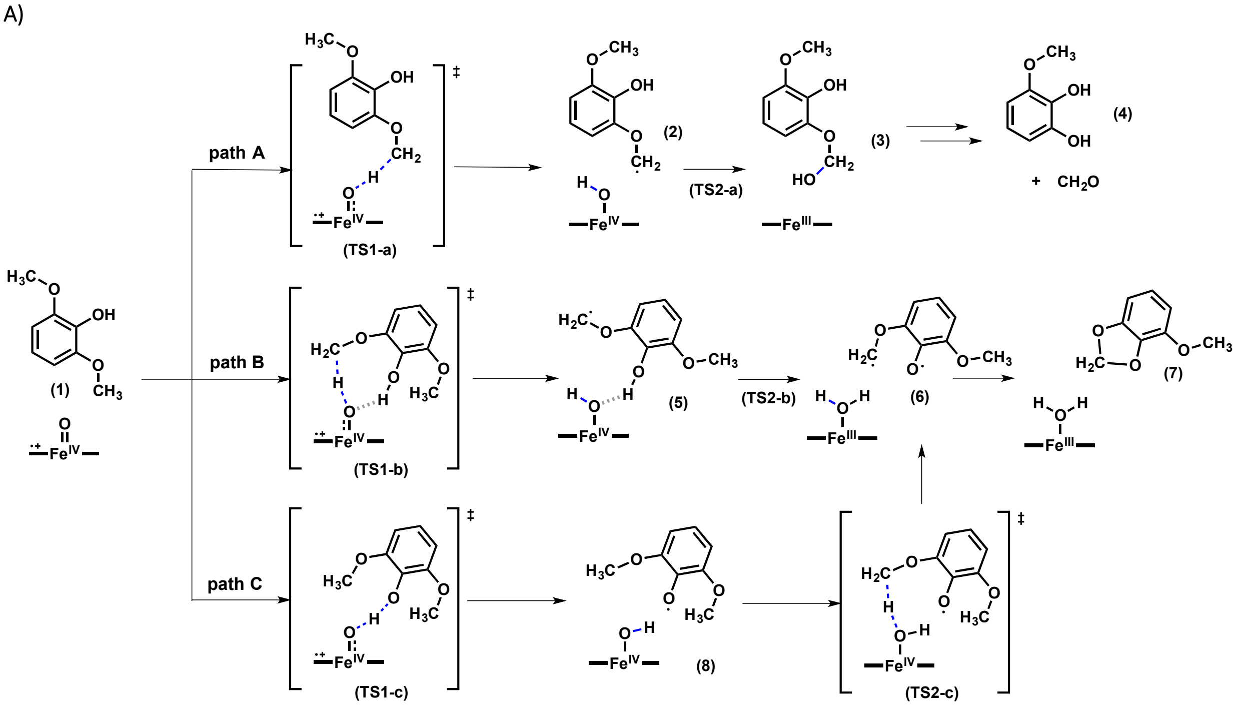
path A
path B
path C
(1)
(TS1-a)
(2)
(TS2-a)
(3)
(4)
+ CH2O
(TS1-b)
(5)
(TS2-b)
(6)
(7)
(TS1-c)
(8)
(TS2-c)

B)

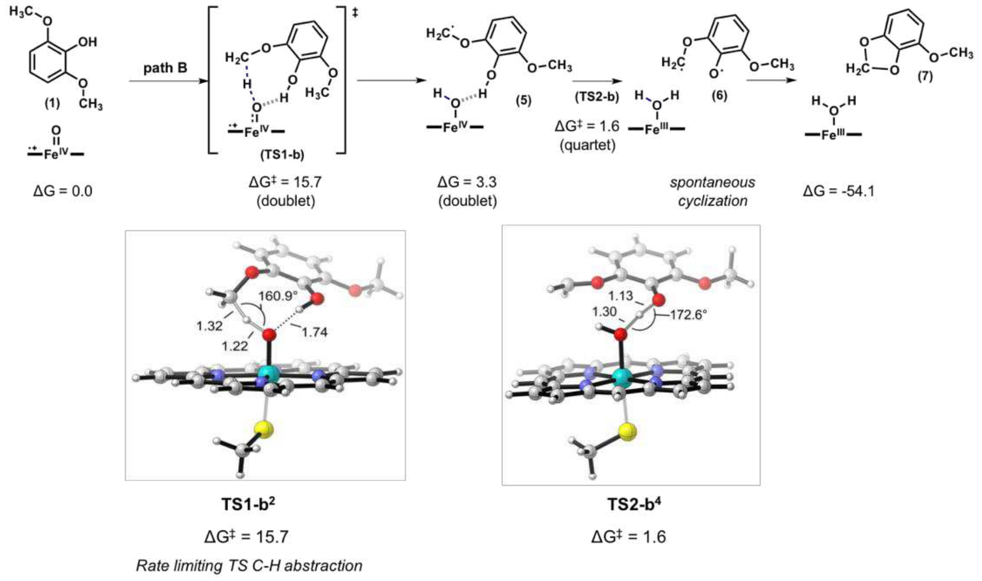
path B
(1)
(TS1-b)
(5)
(TS2-b)
(6)
(7)
ΔG = 0.0
ΔG‡ = 15.7
(doublet)
ΔG = 3.3
(doublet)
ΔG‡ = 1.6
(quartet)
spontaneous
cyclization
ΔG = -54.1
160.9°
1.32
1.22
1.74
1.13
1.30
172.6°
TS1-b2
ΔG‡ = 15.7
Rate limiting TS C-H abstraction
TS2-b4
ΔG‡ = 1.6

C)

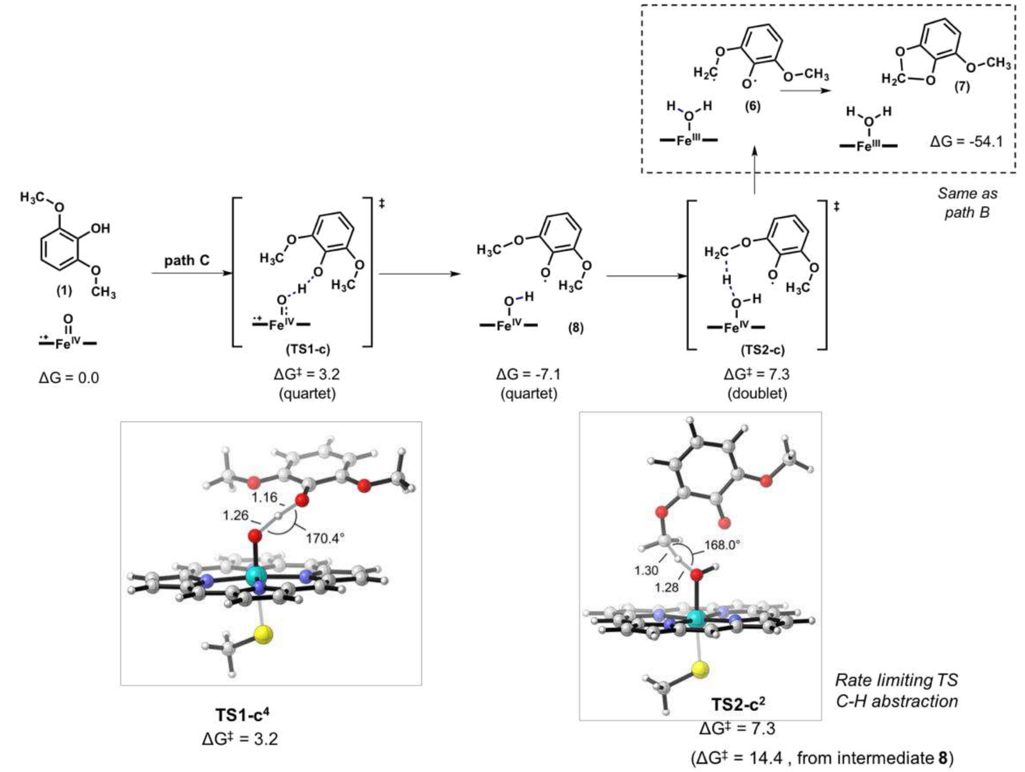


**Figure S11**. **DFT calculations for possible reaction pathways for syringol degradation catalyzed by Compound I (CpI).** A) Target pathway for syringol demethylation to 3MC catalyzed by P450 CpI. Our calculations indicate syringol demethylation follows a similar pathway to that previously described for guaiacol (1). Free energy barriers for the rate-limiting initial hydrogen atom transfer (HAT) from the methoxy group to the heme-bound oxygen atom are very similar for guaiacol and syringol ($\Delta G^{\ddagger}$ = 18.6 and 18.9 kcal·mol$^{-1}$, respectively), and also are the optimized transition state (TS) geometries. Consequently, the demethylation reaction likely proceeds similarly for both substrates when they can bind productively with the CpI reactive species in the enzyme active site. Unproductive pathways **b** (B) and **c** (C) lead to undesired products (and possibly uncoupling) and are intrinsically lower in energy than pathway **a**. This is indicating that enzymatic environment that forces an specific substrate binding pose, as demonstrated by X-ray structures and MD simulations, is preventing pathways **b** and **c** to take place and promotes desired pathway **a**. Gibbs energies, obtained at B3LYP-D3BJ/6-311+G(d,p)+Fe(LanL2DZ)(PCM=Diethylether)//B3LYP/6-31G(d)+Fe(LanL2DZ), are given in kcal/mol. Distances and angles in DFT optimized key TSs are given in Å and degrees, respectively.

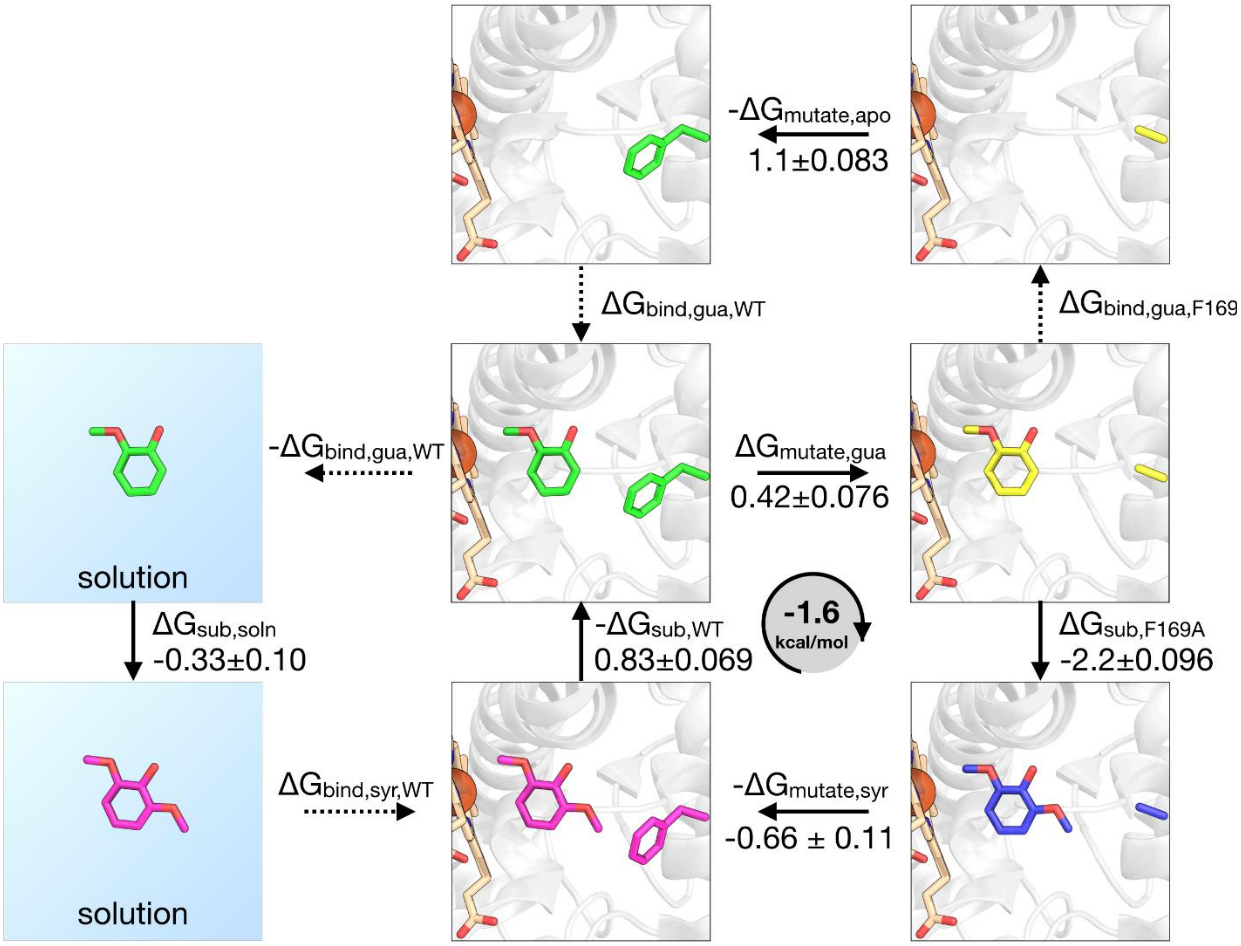


**Figure S12. Replica exchange thermodynamic integration (RETI) simulations indicate the change in free energy for different alchemical transformations.** Each solid arrow indicates a RETI calculation that was performed and has the associated free energy change for the process in the direction of the arrow. Dashed arrows represent transformations that were not performed. A full four-step thermodynamic cycle was completed, represented by the four boxes in the lower right. The closure in performing these four steps theoretically is equal to zero. We calculate a closure of -1.6 kcal/mol, which is on the order of the expected cumulative error one would expect given the errors in the individual steps (approximately 0.2 kcal/mol).

The most surprising of these thermodynamic results is the relatively low free energy difference between syringol and guaiacol bound at the active site of WT GcoA. This can explained by the fact that these simulations indicate that binding syringol in the WT can produce binding complexes in which the substrate is flipped "upside down" from the configuration seen in crystal structures (*see* **Fig. S12**). This may help explain the experimental result that while WT demethylation activity is greatly reduced when exchanging syringol for guaiacol, the binding preference is less dramatic. Substrate flipping could reduce the crowding penalty in WT GcoA. The analogous set of simulations in the GcoA-F169A mutant (as well as mutation transformations with either syringol or guaiacol bound) showed comparatively few flips (for substrate transformation in WT, the substrate was flipped ~90% of the time, compared to ~10% in the other three cases). Experimentally, syringol binding affinity is not much changed from the WT to GcoA-F169A ($K_D$ values indicate a difference in binding free energy of 0.3 kcal·mol$^{-1}$), even as the activity is improved markedly. The RETI results indicate the possibility that this may be because $K_D$ measurements detect all binding at the active site capable of displacing a Fe-coordinating water molecule whereas catalytic turnover will only result from productive binding with catalytically relevant positioning of substrate relative to CpI heme. RETI results present the possibility that this is because the GcoA-F169A mutant is able to keep both substrates oriented correctly whereas WT GcoA is not.

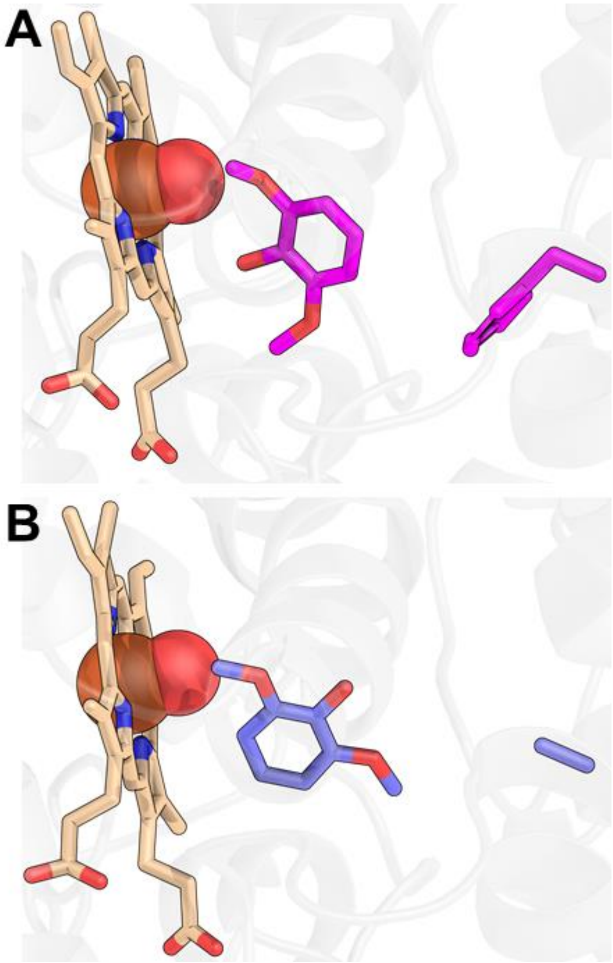


**Figure S13.** Alternate substrate binding configurations seen in RETI simulations. These results indicate a flip of substrate that is unique to the $\Delta G_{sub,WT}$ calculation, which involves an alchemical transformation between guaiacol and syringol in the active site of WT GcoA. Given the differences in $K_D$, activity, and crystal structures, the expected result for this transformation would be that guaiacol would be significantly favored at the WT active site. It is therefore surprising that this transformation results in a free energy change that slightly *favors* syringol (by 0.83 ± 0.069 kcal/mol); this is explained by the fact that the substrate adopts a unique binding pose not seen in crystal structures or any other MD simulations. A) Exemplified by syringol, in all 20 windows (in which syringol is gradually "turned off" and guaiacol is gradually "turned on") of the $\Delta G_{sub,WT}$ transformation, this "upside down" configuration dominates, spending only about 10-15% of the simulation time in a configuration similar to that seen in crystal structures. By contrast, in the other transformations ($\Delta G_{mutate,gua}$, $\Delta G_{sub,F169A}$, and $\Delta G_{mutate,syr}$) the "upright" substrate configuration is displayed in upwards of 90% of the simulation time. This is represented in panel B by a representative frame from the $\Delta G_{sub,F169A}$ simulation. Heme, substrate, and sidechain of residue 169 (Phe in panel A and Ala in panel B) are shown in sticks; heme Fe and Fe-bound oxygen are shown as spheres.

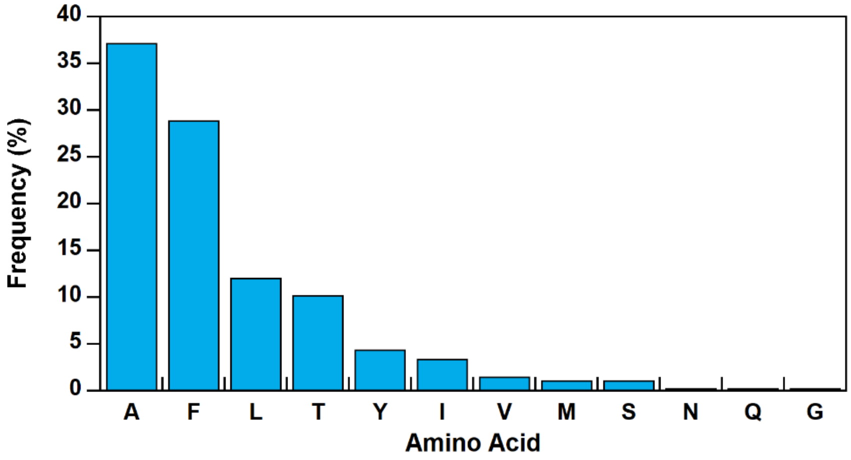


**Figure S14. Frequency of amino acids occurring at position 169 among GcoA homologs.** Ala is more frequent than Phe, and none of the 482 homologs utilizes His at the 169$^{th}$ position.

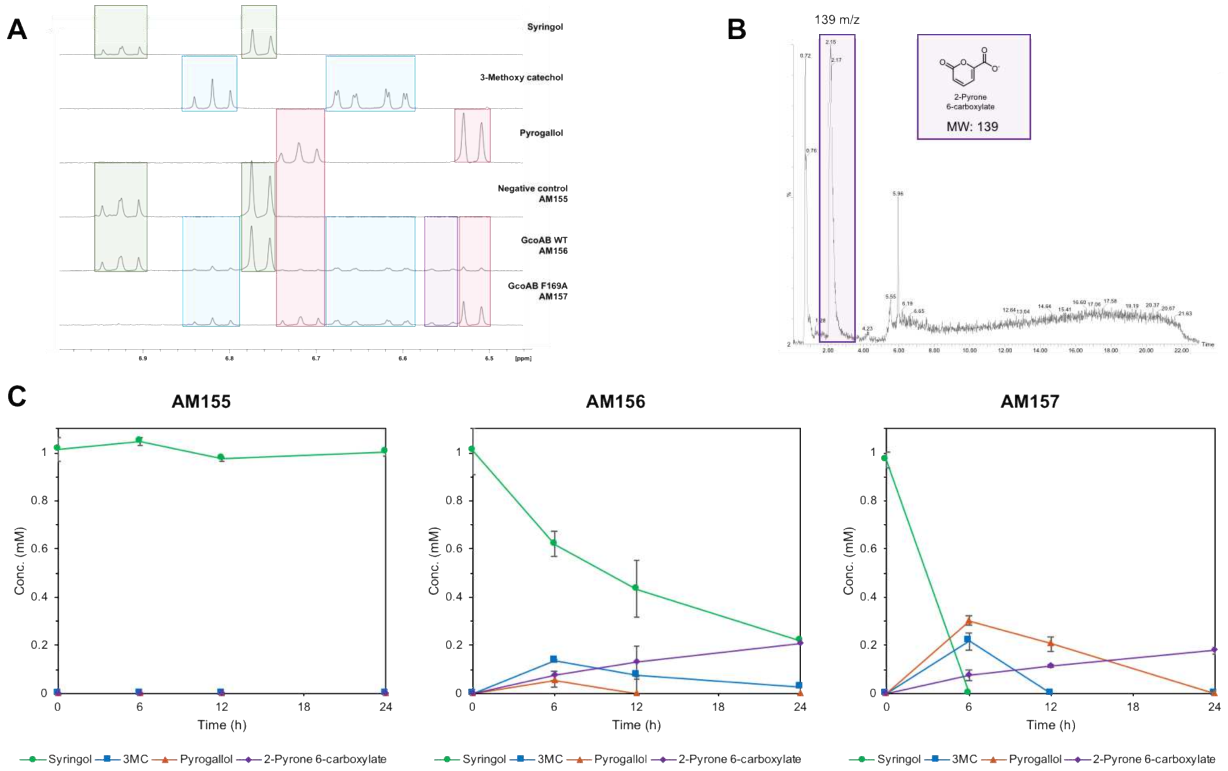


**Figure S15.** ***In vivo* syringol consumption**. A) Expanded version of Figure 5 including standards of syringol, 3MC, and pyrogallol. B) Extracted ion chromatograph of 139 m/z verifying presence of 2-pyrone 6-carboxylate in AM157. C) Time course analysis of syringol depletion and product formation. Absolute analyte concentrations were measured via integration of $^{1}$H NMR peaks, by comparison to an integrated standard peak (TMSP). Plasmid-based expression of $GcoAB_{F169A}$ (AM157) allows for complete turnover of syringol after 6 hours, while syringol still remains after 24 hours in cells expressing WT GcoAB (AM156). Data points represent averages of three replicates, and error bars indicate the standard deviations of the measurements.

Table S1. Rate of substrate disappearance or product appearance (specific activity) for F169A.

| Substrate | NADH disappearance ($\mu M\ s^{-1}\ \mu mol\ F169A^{-1}$) | aromatic substrate disappearance ($\mu M\ s^{-1}\ \mu mol\ F169A^{-1}$) | $K_D$ (μM) | |
|---|---|---|---|---|
| | | | WT | F169A |
| guaiacol | 5.8 ± 0.8 | 5.6 ± 0.3 | 0.0060 ± 0.002 | 7.1 ± 0.1 |
| syringol | 5.1 ± 0.8 | 4.1 ± 0.3 | 2.8 ± 0.4 | 1.7 ± 0.07 |
| 3MC | 5.6 ± 0.8 | 2.6 ± 0.3 | 3.7 ± 0.1 | 9.5 ± 0.02 |

Table S2. Rates of substrate disappearance and coupling efficiencies for reactions catalyzed by the GcoA-F169 variants.

| | syringol | | guaiacol | | 3MC | |
|---|---|---|---|---|---|---|
| GcoA variant | Specific activity ($\mu mol\ sec^{-1}\ \mu mol^{-1}$ GcoA)[a] | Coupling efficiency (%)[b] | Specific activity ($\mu mol\ sec^{-1}\ \mu mol^{-1}$ GcoA)[a] | Coupling efficiency (%)[b] | Specific activity ($\mu mol\ sec^{-1}\ \mu mol^{-1}$ GcoA)[a] | Coupling efficiency (%)[b] |
| WT | n/a | 7.1 ± 0.8 | 5.0 ± 0.1 | 110 ± 10 | 3.2 ± 0.2 | 78 ± 3 |
| F169A | 5.1 ± 0.8 | 104 ± 6 | 5.8 ± 0.8 | 103 ± 7 | 5.6 ± 0.8 | 64 ± 10 |
| F169S | 5.1 ± 0.9 | 85 ± 5 | 5.7 ± 0.8 | 67 ± 8 | 6.0 ± 0.8 | 67 ± 8 |
| F169V | 4.1 ± 0.8 | 100 ± 10 | 5.3 ± 0.2 | 105 ± 2 | 5.7 ± 0.3 | 40 ± 3 |
| F169H | 3.9 ± 0.2 | 56 ± 7 | 7.9 ± 3 | 103 ± 10 | 4.3 ± 0.4 | 28 ± 20 |
| F169I | 0.56 ± 0.2 | 14 ± 2 | 1.4 ± 0.2 | 41 ± 10 | 1.1 ± 0.7 | 10 ± 9 |
| F169L | 0.54 ± 0.1 | 7.8 ± 2 | 4.5 ± 0.3 | 73 ± 3 | 0.57 ± 0.2 | 5.0 ± 4 |

[a]NADH consumption was monitored continuously over time via UV/vis and quantifying with $\varepsilon_{340} = 6.22\ mM^{-1}\ cm^{-1}$ at 25°C, 25 mM HEPES, 50 mM NaCl, pH 7.5 and saturating (200 μM) concentrations of all substrates (syringol, guaiacol, and 3MC).

[b]Calculated as the molar ratio of formaldehyde produced per NADH consumed in a fixed-time assay. Assay conditions: 0.2 μM GcoA variant, 0.2 μM GcoB, 200 μM NADH, 100 μg/mL catalase, 200 μM aromatic substrate in 25 mM HEPES, 50 mM NaCl, pH 7.5, 25°C, 210 μM $O_2$.

Table S3. Uncoupling reactions with GcoA-F169A and guaiacol, syringol, or 3MC.

| Substrate | % Formaldehyde produced[a] | % $H_2O_2$ produced[b] |
|---|---|---|
| guaiacol + 100 μM NADH | 100 ± 10 | 3.7 ± 0.6 |
| syringol + 100 μM NADH | 125 ± 6 | 7.4 ± 0.03 |
| syringol + 200 μM NADH | 91 ± 8 | 7.9 ± 0.5 |
| 3-MC + 100 μM NADH | 52 ± 7 | 17 ± 0.2 |

[a]0.2 μM GcoA-F169A and GcoB were reacted with 100 μg catalase, 100 or 200 μM NADH, and 100 μM substrate in 25 mM HEPES, 50 mM NaCl, pH 7.5, 25 °C, 210 μM $O_2$. Endpoint analyses were done to quantify formaldehyde produced (see Methods), which was then referenced to NADH consumed.

[b]The same reaction done above was repeated to quantify $H_2O_2$ produced using the Amplex Red/HRP assay (see *SI* Appendix, Methods). The amount of hydrogen peroxide was then referenced to NADH consumed.

**Table S4**. X-ray tables for GcoA syringol-bound structures

| Protein | F169A with syringol | F169H with syringol | F169S with syringol | F169V with syringol |
|---|---|---|---|---|
| PDB code | 6HQQ | 6HQR | 6HQS | 6HQT |
| Data collection | | | | |
| Space group | P43212 | P43212 | P43212 | P43212 |
| Wavelength (Å) | 0.9795 | 0.9795 | 0.9795 | 0.9795 |
| Cell dimension | | | | |
| a, b, c (Å) | 104.07, 104.07, 116.10 | 102.15, 102.15, 109.98 | 104.25, 104.25, 114.03 | 103.90, 103.90, 115.58 |
| α, β, γ (˚) | 90, 90, 90 | 90, 90, 90 | 90, 90, 90 | 90, 90, 90 |
| Resolution range (Å) | 77.49-1.66 (1.70-1.66) | 72.23-1.79 (1.84-1.79) | 61.91-2.17 (2.23-2.17) | 62.00-1.85 (1.90-1.85) |
| Rmerge | 0.066 (1.244) | 0.081 (0.938) | 0.066 (0.816) | 0.070 (0.813) |
| Rmeas | 0.071 (1.351) | 0.087 (1.018) | 0.072 (0.888) | 0.075 (0.917) |
| Rpim | 0.027 (0.525) | 0.034 (0.393) | 0.028 (0.347) | 0.029 (0.415) |
| I/σI | 19.0 (2.0) | 15.8 (2.2) | 20.9 (3.0) | 22.5 (2.3) |
| Completeness (%) | 100 (100) | 100.0 (100.0) | 100.0 (100.0) | 99.9 (99.9) |
| Multiplicity | 12.9 (12.7) | 12.5 (12.7) | 12.7 (12.3) | 12.7 (8.9) |
| CC 1/2 | 1.00 (0.794) | 0.999 (0.878) | 0.999 (0.865) | 1.000 (0.809) |
| Refinement | | | | |
| Resolution range (Å) | 73.59-1.66 (1.69-1.66) | 51.05-1.79 (1.82-1.79) | 52.15-2.17 (2.23-2.17) | 50.50-1.85 (1.88-1.85) |
| No. Of reflections | 75469 | 53703 | 33716 | 54478 |
| Rwork | 0.1676 (0.3303) | 0.1692 (0.2999) | 0.1608 (0.2507) | 0.1563 (0.2428) |
| Rfree | 0.1909 (0.3748) | 0.1991 (0.3939) | 0.1858 (0.2932) | 0.1788 (0.2987) |
| No. Of atoms | 3752 | 3629 | 3411 | 3601 |
| Protein | 3151 | 3157 | 3122 | 3154 |
| Ligand/ion | 54 | 54 | 54 | 54 |
| Water | 547 | 418 | 235 | 393 |
| B-factors | 21.9 | 21.97 | 37.01 | 27.66 |
| Protein | 27.19 | 24.89 | 41.15 | 29.53 |
| Ligand/ion | 18.06 | 15.15 | 28.99 | 21.89 |
| Water | 41.41 | 37.93 | 45.92 | 41.32 |
| Rmsd | | | | |
| Bond lengths (Å) | 0.008 | 0.016 | 0.008 | 0.007 |
| Bond angles (˚) | 1.012 | 1.353 | 0.938 | 0.9 |

Table S5. X-ray tables for GcoA guaiacol-bound structures

| Protein | F169A with guaiacol | F169H with guaiacol | F169I with guaiacol | F169L with guaiacol | F169S with guaiacol | F169V with guaiacol |
|---|---|---|---|---|---|---|
| PDB code | 6HQK | 6HQL | 6HQM | 6HQN | 6HQO | 6HQP |
| Data collection | | | | | | |
| Space group | P43212 | P43212 | P43212 | P43212 | P43212 | P43212 |
| Wavelength (Å) | 0.9795 | 0.9795 | 0.9795 | 0.9795 | 0.9795 | 0.9795 |
| Cell dimension | | | | | | |
| a, b, c (Å) | 103.67, 103.67, 114.88 | 102.00, 102.00, 109.83 | 105.20, 105.20, 112.52 | 104.00, 104.00, 111.58 | 103.85, 103.85. 115.10 | 103.80, 103.80, 115,78 |
| α, β, γ (˚) | 90, 90, 90 | 90, 90, 90 | 90, 90, 90 | 90, 90, 90 | 90, 90, 90 | 90, 90, 90 |
| Resolution range (Å) | 76.97-1.57 (1.61-1.57) | 60.29-1.49 (1.53-1.49) | 56.25-1.85 (1.90-1.85) | 76.08-1.87 (1.92-1.87) | 73.43-1.70 (1.74-1.70) | 73.40-1.62 (1.66-1.62) |
| Rmerge | 0.071 (0.964) | 0.077 (1.040) | 0.064 (1.002) | 0.080 (0.991) | 0.064 (1.309) | 0.069 (0.985) |
| Rmeas | 0.077 (1.068) | 0.080 (1.040) | 0.069 (1.085) | 0.087 (1.076) | 0.069 (1.420) | 0.075 (1.096) |
| Rpim | 0.029 (0.457) | 0.032 (0.449) | 0.027 (0.415) | 0.034 (0.417) | 0.026 (0.547) | 0.029 (0.343) |
| I/σI | 18.3 (2.2) | 14.9 (2.3) | 10.4 (2.6) | 17.0 (2.4) | 24.0 (2.0) | 17.4 (2.1) |
| completeness (%) | 100 (100) | 100.0 (100.0) | 100.0 (100.0) | 100.0 (100.0) | 100.0 (100.0) | 100.0 (100.0) |
| multiplicity | 12.5 (10.5) | 12.6 (12.2) | 12.7 (13.1) | 12.7 (12.7) | 13.1 (12.9) | 12.4 (10.2) |
| CC 1/2 | 0.999 (0.802) | 0.999 (100.0) | 1.000 (0.849) | 0.999 (0.808) | 1.000 (0.786) | 0.999 (0.828) |
| Refinement | | | | | | |
| Resolution range (Å) | 76.98-1.57 (1.59-1.57) | 60.00-1.49 (1.51-1.49) | 52.61-1.85 (1.88-1.85) | 73.54-1.87 (1.91-1.87) | 73.43-1.70 (1.72-1.70) | 73.40-1.62 (1.64-1.62) |
| No. Of reflections | 87452 | 93998 | 54123 | 51138 | 69625 | 80413 |
| Rwork | 0.1581 (0.2897) | 0.1744 (0.3340) | 0.1610 (0.3039) | 0.1455 (0.2439) | 0.1517 (0.2641) | 0.1661 (0.3377) |
| Rfree | 0.1812 (0.3208) | 0.1984 (0.3800) | 0.1799 (0.3934) | 0.1697 (0.2627) | 0.1811 (0.3150) | 0.1874 (0.3602) |
| No. Of atoms | 3744 | 3655 | 3578 | 3650 | 3684 | 3697 |
| Protein | 3161 | 3157 | 3087 | 3158 | 3144 | 3154 |
| Ligand/ion | 52 | 52 | 52 | 52 | 52 | 52 |
| Water | 531 | 446 | 439 | 440 | 488 | 491 |
| B-factors | 16.87 | 17.15 | 27.3 | 33.44 | 25.32 | 19.35 |
| Protein | 20.56 | 21.82 | 28.17 | 35.61 | 29.52 | 23.73 |
| Ligand/ion | 13.52 | 13.31 | 18.97 | 25.26 | 21.79 | 15.97 |
| Water | 35.47 | 36.19 | 41.21 | 44.83 | 42.7 | 38.27 |
| Rmsd | | | | | | |
| Bond lengths (Å) | 0.016 | 0.014 | 0.009 | 0.013 | 0.018 | 0.016 |
| Bond angles (˚) | 1.455 | 1.371 | 1.042 | 1.319 | 1.456 | 1.368 |

**Table S6**. Gibbs energy profiles computed at B3LYP-D3BJ/6-311+G(d,p)+Fe(LanL2DZ)(PCM=Diethylether)//B3LYP/6-31G(d)+Fe(LanL2DZ), considering doublet and quadruplet electronic states. Energies are given in kcal/mol.

| ΔG | | | | | |
|---|---|---|---|---|---|
| Path A | Reactants | TS1-a | Int1-a complex | TS2-a | Product 4 formation |
| doublet | 0.2 | 18.6 | *(barrierless)* | | -55.7 |
| quartet | 0.0 | 20.8 | 12.6 | 15.1 | -55.7 |

| ΔG | | | | | |
|---|---|---|---|---|---|
| Path B | Reactants | TS1 | int - complex | TS2 | Product 7 formation |
| doublet | 0.2 | 15.7 | 3.3 | *(barrierless)* | -52.4 |
| quartet | 0.0 | 15.7 | 5.8 | 1.6 | -52.4 |

| ΔG | | | | | |
|---|---|---|---|---|---|
| Path C | Reactants | TS1 | int - complex | TS2 | Product 7 formation |
| doublet | 0.2 | 4.3 | (*could not be optimized*) | 7.3 | -52.4 |
| quartet | 0.0 | 3.2 | -7.1 | 8.4 | -52.4 |

Table S7. GcoA Sequence conservation analysis.

| resid (GcoA) | AA (GcoA) | relative entropy | z-score | percentile |
|---|---|---|---|---|
| 1 | M | 3.28 | 2.59 | 98.0 |
| 2 | T | 1.42 | -0.18 | 51.4 |
| 3 | T | 0.95 | -0.89 | 16.5 |
| 4 | T | 1.44 | -0.16 | 52.6 |
| 5 | E | 0.59 | -1.42 | 1.7 |
| 6 | R | 0.63 | -1.36 | 2.7 |
| 7 | P | 0.69 | -1.28 | 4.7 |
| 8 | D | 0.79 | -1.12 | 9.1 |
| 9 | L | 0.67 | -1.31 | 3.7 |
| 10 | A | 1.02 | -0.78 | 20.9 |
| 11 | W | 1.49 | -0.09 | 55.5 |
| 12 | L | 0.96 | -0.87 | 17.7 |
| 13 | D | 1.04 | -0.75 | 21.6 |
| 14 | E | 1.10 | -0.67 | 27.8 |
| 15 | V | 1.67 | 0.19 | 64.4 |
| 16 | T | 2.10 | 0.83 | 81.3 |
| 17 | M | 1.10 | -0.67 | 28.0 |
| 18 | T | 0.84 | -1.06 | 11.8 |
| 19 | Q | 1.28 | -0.39 | 43.2 |
| 20 | L | 2.17 | 0.94 | 83.8 |
| 21 | E | 0.96 | -0.87 | 17.4 |
| 22 | R | 0.84 | -1.06 | 11.5 |
| 23 | N | 2.14 | 0.89 | 83.0 |
| 24 | P | 2.56 | 1.52 | 91.6 |
| 25 | Y | 3.15 | 2.40 | 97.3 |
| 26 | E | 1.95 | 0.61 | 77.6 |
| 27 | V | 1.30 | -0.37 | 44.2 |
| 28 | Y | 3.24 | 2.54 | 97.5 |
| 29 | E | 1.05 | -0.74 | 22.6 |
| 30 | R | 2.11 | 0.84 | 81.6 |
| 31 | L | 1.98 | 0.65 | 78.6 |
| 32 | R | 2.43 | 1.32 | 88.2 |
| 33 | A | 0.98 | -0.85 | 18.9 |
| 34 | E | 1.81 | 0.39 | 71.5 |
| 35 | A | 1.20 | -0.52 | 36.9 |
| 36 | P | 2.43 | 1.32 | 88.5 |
| 37 | L | 1.74 | 0.29 | 67.8 |
| 38 | A | 1.20 | -0.51 | 37.3 |
| 39 | F | 1.64 | 0.15 | 62.9 |
| 40 | V | 1.79 | 0.36 | 70.0 |
| 41 | P | 1.61 | 0.10 | 61.4 |
| 42 | V | 0.88 | -1.00 | 13.5 |
| 43 | L | 0.97 | -0.85 | 18.7 |
| 44 | G | 1.62 | 0.11 | 62.2 |
| 45 | S | 0.75 | -1.19 | 6.6 |
| 46 | Y | 1.65 | 0.16 | 63.6 |
| 47 | V | 1.38 | -0.25 | 47.7 |
| 48 | A | 1.28 | -0.39 | 43.5 |
| 49 | S | 2.13 | 0.88 | 82.6 |
| 50 | T | 1.53 | -0.03 | 57.5 |
| 51 | A | 1.09 | -0.68 | 26.8 |
| 52 | E | 1.19 | -0.54 | 35.9 |
| 53 | V | 0.92 | -0.93 | 15.5 |
| 54 | C | 2.79 | 1.86 | 95.6 |
| 55 | R | 1.06 | -0.72 | 24.1 |
| 56 | E | 0.73 | -1.22 | 6.1 |
| 57 | V | 1.71 | 0.25 | 66.1 |
| 58 | A | 1.07 | -0.71 | 24.8 |
| 59 | T | 0.69 | -1.28 | 4.2 |
| 60 | S | 1.37 | -0.27 | 47.4 |
| 61 | P | 1.01 | -0.80 | 19.9 |
| 62 | D | 1.06 | -0.72 | 24.3 |
| 63 | F | 2.67 | 1.69 | 94.3 |
| 64 | E | 1.17 | -0.56 | 34.2 |
| 65 | A | 1.30 | -0.37 | 44.7 |
| 66 | V | 0.82 | -1.08 | 10.6 |
| 67 | I | 0.96 | -0.87 | 17.2 |
| 68 | T | 1.03 | -0.77 | 21.4 |
| 69 | P | 1.02 | -0.79 | 20.6 |
| 70 | A | 1.06 | -0.72 | 23.6 |

| resid (GcoA) | AA (GcoA) | relative entropy | z-score | percentile |
|---|---|---|---|---|
| 71 | G | 1.25 | -0.43 | 41.3 |
| 72 | G | 0.84 | -1.06 | 11.3 |
| 73 | R | 1.87 | 0.48 | 74.9 |
| 74 | T | 1.27 | -0.41 | 41.8 |
| 75 | F | 2.23 | 1.02 | 85.5 |
| 76 | G | 2.00 | 0.68 | 79.4 |
| 77 | H | 1.18 | -0.55 | 34.6 |
| 78 | P | 1.61 | 0.10 | 61.7 |
| 79 | A | 1.32 | -0.34 | 45.7 |
| 80 | I | 1.48 | -0.10 | 54.8 |
| 81 | I | 1.81 | 0.40 | 71.7 |
| 82 | G | 1.12 | -0.63 | 30.7 |
| 83 | V | 1.46 | -0.12 | 53.8 |
| 84 | N | 1.84 | 0.44 | 73.2 |
| 85 | G | 2.06 | 0.77 | 80.6 |
| 86 | D | 1.29 | -0.37 | 44.0 |
| 87 | I | 0.83 | -1.07 | 10.8 |
| 88 | H | 3.61 | 3.09 | 99.3 |
| 89 | A | 0.76 | -1.17 | 7.4 |
| 90 | D | 0.79 | -1.12 | 9.3 |
| 91 | L | 1.44 | -0.15 | 53.1 |
| 92 | R | 2.60 | 1.58 | 93.1 |
| 93 | S | 0.78 | -1.15 | 8.1 |
| 94 | M | 1.18 | -0.55 | 34.9 |
| 95 | V | 1.74 | 0.29 | 67.3 |
| 96 | E | 1.15 | -0.60 | 31.9 |
| 97 | P | 1.69 | 0.22 | 65.1 |
| 98 | A | 0.85 | -1.04 | 12.0 |
| 99 | L | 1.86 | 0.47 | 74.0 |
| 100 | Q | 1.38 | -0.24 | 48.2 |
| 101 | P | 2.15 | 0.91 | 83.3 |
| 102 | A | 1.00 | -0.81 | 19.7 |
| 103 | E | 0.74 | -1.21 | 6.4 |
| 104 | V | 1.80 | 0.38 | 71.3 |
| 105 | D | 1.04 | -0.75 | 21.9 |
| 106 | R | 0.72 | -1.24 | 5.7 |
| 107 | W | 1.50 | -0.07 | 56.5 |
| 108 | I | 1.58 | 0.06 | 59.7 |
| 109 | D | 0.80 | -1.12 | 9.8 |
| 110 | D | 0.90 | -0.96 | 14.7 |
| 111 | L | 1.39 | -0.23 | 48.6 |
| 112 | V | 1.51 | -0.04 | 57.0 |
| 113 | R | 1.42 | -0.19 | 51.1 |
| 114 | P | 1.09 | -0.67 | 27.5 |
| 115 | I | 1.40 | -0.21 | 49.9 |
| 116 | A | 1.49 | -0.08 | 55.8 |
| 117 | R | 1.09 | -0.68 | 26.5 |
| 118 | R | 0.81 | -1.10 | 10.1 |
| 119 | Y | 1.91 | 0.55 | 76.7 |
| 120 | L | 1.40 | -0.21 | 50.1 |
| 121 | E | 0.97 | -0.86 | 18.4 |
| 122 | R | 0.77 | -1.16 | 7.9 |
| 123 | F | 1.65 | 0.16 | 63.9 |
| 124 | E | 1.10 | -0.66 | 29.0 |
| 125 | N | 0.75 | -1.18 | 6.9 |
| 126 | D | 0.88 | -1.00 | 14.0 |
| 127 | G | 2.33 | 1.18 | 87.2 |
| 128 | H | 0.78 | -1.14 | 8.8 |
| 129 | A | 1.11 | -0.66 | 29.2 |
| 130 | E | 1.87 | 0.49 | 75.2 |
| 131 | L | 1.96 | 0.63 | 77.9 |
| 132 | V | 1.20 | -0.52 | 36.4 |
| 133 | A | 0.65 | -1.33 | 3.2 |
| 134 | Q | 1.11 | -0.65 | 29.5 |
| 135 | Y | 2.23 | 1.03 | 85.7 |
| 136 | C | 2.19 | 0.97 | 84.5 |
| 137 | E | 1.43 | -0.17 | 51.8 |
| 138 | P | 2.58 | 1.55 | 91.9 |
| 139 | V | 1.55 | 0.01 | 59.0 |
| 140 | S | 1.91 | 0.54 | 76.4 |
| 141 | V | 1.21 | -0.49 | 38.3 |

| resid (GcoA) | AA (GcoA) | relative entropy | z-score | percentile |
|---|---|---|---|---|
| 142 | R | 1.61 | 0.10 | 60.9 |
| 143 | S | 1.32 | -0.33 | 46.4 |
| 144 | L | 1.85 | 0.46 | 73.5 |
| 145 | G | 1.17 | -0.56 | 34.4 |
| 146 | D | 1.23 | -0.47 | 39.8 |
| 147 | L | 1.24 | -0.45 | 40.5 |
| 148 | L | 1.78 | 0.36 | 69.8 |
| 149 | G | 2.62 | 1.60 | 93.6 |
| 150 | L | 1.74 | 0.29 | 67.6 |
| 151 | Q | 0.43 | -1.66 | 0.2 |
| 152 | E | 1.22 | -0.48 | 39.1 |
| 153 | V | 1.30 | -0.37 | 44.5 |
| 154 | D | 1.21 | -0.49 | 38.1 |
| 155 | S | 0.86 | -1.02 | 12.5 |
| 156 | D | 1.09 | -0.68 | 27.3 |
| 157 | K | 1.47 | -0.10 | 54.3 |
| 158 | L | 1.93 | 0.58 | 77.1 |
| 159 | R | 1.32 | -0.33 | 46.7 |
| 160 | E | 1.32 | -0.34 | 45.9 |
| 161 | W | 3.88 | 3.50 | 99.8 |
| 162 | F | 2.38 | 1.26 | 87.7 |
| 163 | A | 1.89 | 0.52 | 76.2 |
| 164 | K | 0.72 | -1.23 | 5.9 |
| 165 | L | 1.99 | 0.66 | 78.9 |
| 166 | N | 1.22 | -0.48 | 39.3 |
| 167 | R | 0.71 | -1.25 | 5.2 |
| 168 | S | 1.75 | 0.30 | 68.3 |
| 169 | F | 1.09 | -0.68 | 27.0 |
| 170 | T | 1.30 | -0.36 | 45.2 |
| 171 | N | 3.30 | 2.63 | 98.3 |
| 172 | A | 1.14 | -0.61 | 31.0 |
| 173 | A | 0.82 | -1.09 | 10.3 |
| 174 | V | 0.97 | -0.86 | 17.9 |
| 175 | D | 1.54 | 0.00 | 58.5 |
| 176 | E | 1.64 | 0.15 | 62.7 |
| 177 | N | 1.93 | 0.57 | 76.9 |
| 178 | G | 2.62 | 1.60 | 93.9 |
| 179 | E | 1.83 | 0.44 | 73.0 |
| 180 | F | 3.02 | 2.21 | 96.3 |
| 181 | A | 1.41 | -0.20 | 50.4 |
| 182 | N | 2.72 | 1.75 | 94.8 |
| 183 | P | 1.67 | 0.19 | 64.6 |
| 184 | E | 1.24 | -0.46 | 40.0 |
| 185 | G | 0.84 | -1.06 | 11.1 |
| 186 | F | 1.97 | 0.64 | 78.4 |
| 187 | A | 0.63 | -1.37 | 2.5 |
| 188 | E | 0.66 | -1.33 | 3.4 |
| 189 | G | 1.16 | -0.58 | 33.2 |
| 190 | D | 1.82 | 0.41 | 72.2 |
| 191 | Q | 0.78 | -1.14 | 8.4 |
| 192 | A | 1.48 | -0.10 | 54.5 |
| 193 | K | 1.28 | -0.40 | 42.8 |
| 194 | A | 0.94 | -0.91 | 15.7 |
| 195 | E | 1.86 | 0.47 | 74.2 |
| 196 | I | 1.83 | 0.43 | 72.5 |
| 197 | R | 0.76 | -1.17 | 7.1 |
| 198 | A | 0.94 | -0.90 | 16.2 |
| 199 | V | 0.79 | -1.12 | 9.6 |
| 200 | V | 1.40 | -0.22 | 49.6 |
| 201 | D | 1.08 | -0.69 | 25.8 |
| 202 | P | 1.03 | -0.77 | 21.1 |
| 203 | L | 1.06 | -0.72 | 23.8 |
| 204 | I | 1.16 | -0.57 | 33.4 |
| 205 | D | 1.15 | -0.59 | 32.4 |
| 206 | K | 1.14 | -0.60 | 31.4 |
| 207 | W | 1.50 | -0.07 | 56.8 |
| 208 | I | 0.86 | -1.02 | 12.8 |
| 209 | E | 0.54 | -1.51 | 0.7 |
| 210 | H | 1.16 | -0.58 | 32.9 |
| 211 | P | 2.54 | 1.49 | 90.4 |
| 212 | D | 1.87 | 0.49 | 75.7 |

| resid (GcoA) | AA (GcoA) | relative entropy | z-score | percentile |
|---|---|---|---|---|
| 213 | D | 0.90 | -0.96 | 15.0 |
| 214 | S | 2.21 | 1.00 | 85.0 |
| 215 | A | 1.10 | -0.66 | 28.5 |
| 216 | I | 1.41 | -0.20 | 50.6 |
| 217 | S | 2.84 | 1.94 | 95.8 |
| 218 | H | 1.82 | 0.41 | 72.0 |
| 219 | W | 2.23 | 1.03 | 86.0 |
| 220 | L | 1.38 | -0.24 | 47.9 |
| 221 | H | 2.10 | 0.82 | 81.1 |
| 222 | D | 1.17 | -0.56 | 33.9 |
| 223 | G | 1.47 | -0.11 | 54.1 |
| 224 | M | 1.87 | 0.48 | 74.7 |
| 225 | P | 2.22 | 1.02 | 85.3 |
| 226 | P | 1.44 | -0.16 | 52.8 |
| 227 | G | 2.48 | 1.40 | 89.2 |
| 228 | Q | 1.66 | 0.17 | 64.1 |
| 229 | T | 1.53 | -0.01 | 58.2 |
| 230 | R | 1.58 | 0.06 | 60.0 |
| 231 | D | 1.15 | -0.59 | 32.2 |
| 232 | R | 0.77 | -1.16 | 7.6 |
| 233 | E | 1.19 | -0.53 | 36.1 |
| 234 | Y | 1.24 | -0.46 | 40.3 |
| 235 | I | 2.05 | 0.75 | 80.1 |
| 236 | Y | 1.70 | 0.23 | 65.4 |
| 237 | P | 1.59 | 0.07 | 60.4 |
| 238 | T | 2.13 | 0.88 | 82.3 |
| 239 | I | 1.55 | 0.00 | 58.7 |
| 240 | Y | 2.26 | 1.07 | 86.2 |
| 241 | V | 1.72 | 0.27 | 66.6 |
| 242 | Y | 1.14 | -0.60 | 31.2 |
| 243 | L | 1.73 | 0.28 | 67.1 |
| 244 | L | 1.43 | -0.17 | 51.6 |
| 245 | G | 2.44 | 1.35 | 88.9 |
| 246 | A | 1.80 | 0.38 | 71.0 |
| 247 | M | 1.87 | 0.49 | 75.4 |
| 248 | Q | 2.73 | 1.78 | 95.1 |
| 249 | E | 2.54 | 1.50 | 90.9 |
| 250 | P | 2.54 | 1.49 | 90.7 |
| 251 | G | 1.73 | 0.27 | 66.8 |
| 252 | H | 2.52 | 1.46 | 90.2 |
| 253 | G | 1.18 | -0.54 | 35.1 |
| 254 | M | 1.28 | -0.40 | 42.5 |
| 255 | A | 1.01 | -0.79 | 20.1 |
| 256 | S | 1.79 | 0.36 | 70.3 |
| 257 | T | 1.43 | -0.16 | 52.3 |
| 258 | L | 1.40 | -0.22 | 49.1 |
| 259 | V | 1.08 | -0.70 | 25.3 |
| 260 | G | 2.11 | 0.85 | 81.8 |
| 261 | L | 2.37 | 1.24 | 87.5 |
| 262 | F | 1.68 | 0.20 | 64.9 |
| 263 | S | 1.04 | -0.75 | 22.1 |
| 264 | R | 1.61 | 0.11 | 61.9 |
| 265 | P | 2.19 | 0.97 | 84.3 |
| 266 | E | 1.57 | 0.03 | 59.2 |
| 267 | Q | 3.39 | 2.76 | 98.5 |
| 268 | L | 1.32 | -0.34 | 46.2 |
| 269 | E | 1.16 | -0.57 | 33.7 |
| 270 | E | 0.94 | -0.91 | 16.0 |
| 271 | V | 2.06 | 0.77 | 80.3 |
| 272 | V | 0.89 | -0.98 | 14.3 |
| 273 | D | 0.87 | -1.01 | 13.0 |
| 274 | D | 1.53 | -0.03 | 57.2 |
| 275 | P | 2.16 | 0.92 | 83.5 |
| 276 | T | 0.57 | -1.45 | 1.0 |
| 277 | L | 1.12 | -0.63 | 30.5 |
| 278 | I | 1.48 | -0.09 | 55.3 |
| 279 | P | 1.10 | -0.67 | 28.3 |
| 280 | R | 1.14 | -0.60 | 31.7 |
| 281 | A | 1.88 | 0.50 | 75.9 |
| 282 | I | 1.76 | 0.32 | 68.8 |
| 283 | A | 1.05 | -0.73 | 22.9 |

| resid (GcoA) | AA (GcoA) | relative entropy | z-score | percentile |
|---|---|---|---|---|
| 284 | E | 2.65 | 1.66 | 94.1 |
| 285 | G | 1.76 | 0.32 | 68.6 |
| 286 | L | 1.39 | -0.24 | 48.4 |
| 287 | R | 2.60 | 1.58 | 92.9 |
| 288 | W | 3.80 | 3.37 | 99.5 |
| 289 | T | 1.32 | -0.33 | 46.9 |
| 290 | S | 1.64 | 0.15 | 63.1 |
| 291 | P | 2.67 | 1.69 | 94.6 |
| 292 | I | 2.59 | 1.57 | 92.6 |
| 293 | W | 3.60 | 3.07 | 99.0 |
| 294 | S | 1.75 | 0.30 | 68.1 |
| 295 | A | 1.18 | -0.54 | 35.4 |
| 296 | T | 1.28 | -0.40 | 43.0 |
| 297 | A | 0.90 | -0.96 | 14.5 |
| 298 | R | 2.38 | 1.26 | 88.0 |
| 299 | I | 0.96 | -0.87 | 17.0 |
| 300 | S | 1.12 | -0.64 | 30.2 |
| 301 | T | 1.48 | -0.09 | 55.0 |
| 302 | K | 1.06 | -0.73 | 23.1 |
| 303 | P | 1.53 | -0.02 | 57.7 |
| 304 | V | 1.44 | -0.15 | 53.3 |
| 305 | T | 1.39 | -0.23 | 48.9 |
| 306 | I | 1.49 | -0.08 | 56.0 |
| 307 | A | 0.97 | -0.86 | 18.2 |
| 308 | G | 2.30 | 1.14 | 86.5 |
| 309 | V | 1.29 | -0.38 | 43.7 |
| 310 | D | 0.48 | -1.59 | 0.5 |
| 311 | L | 1.83 | 0.43 | 72.7 |
| 312 | P | 1.78 | 0.35 | 69.5 |
| 313 | A | 1.24 | -0.45 | 41.0 |
| 314 | G | 2.31 | 1.15 | 87.0 |
| 315 | T | 1.02 | -0.79 | 20.4 |
| 316 | P | 0.62 | -1.38 | 2.2 |
| 317 | V | 2.05 | 0.75 | 79.9 |
| 318 | M | 1.22 | -0.48 | 39.6 |
| 319 | L | 0.85 | -1.03 | 12.3 |
| 320 | S | 1.18 | -0.54 | 35.6 |
| 321 | Y | 1.77 | 0.33 | 69.0 |
| 322 | G | 1.60 | 0.08 | 60.7 |
| 323 | S | 2.43 | 1.33 | 88.7 |
| 324 | A | 2.19 | 0.97 | 84.8 |
| 325 | N | 2.97 | 2.14 | 96.1 |
| 326 | H | 2.13 | 0.88 | 82.1 |
| 327 | D | 2.49 | 1.41 | 89.4 |
| 328 | T | 1.26 | -0.43 | 41.5 |
| 329 | G | 0.69 | -1.28 | 4.4 |
| 330 | K | 0.78 | -1.14 | 8.6 |
| 331 | Y | 2.51 | 1.44 | 89.7 |
| 332 | E | 0.92 | -0.93 | 15.2 |
| 333 | A | 1.11 | -0.65 | 29.7 |
| 334 | P | 1.77 | 0.33 | 69.3 |
| 335 | S | 1.10 | -0.66 | 28.7 |
| 336 | Q | 0.61 | -1.40 | 2.0 |
| 337 | Y | 2.79 | 1.86 | 95.3 |
| 338 | D | 1.99 | 0.67 | 79.1 |
| 339 | L | 1.41 | -0.19 | 50.9 |
| 340 | H | 1.22 | -0.49 | 38.8 |
| 341 | R | 2.60 | 1.58 | 93.4 |
| 342 | P | 0.99 | -0.83 | 19.2 |
| 343 | P | 1.27 | -0.41 | 42.0 |
| 344 | L | 0.69 | -1.28 | 4.9 |
| 345 | P | 1.53 | -0.02 | 58.0 |
| 346 | H | 3.27 | 2.58 | 97.8 |
| 347 | L | 1.43 | -0.16 | 52.1 |
| 348 | A | 1.59 | 0.07 | 60.2 |
| 349 | F | 3.12 | 2.36 | 96.8 |
| 350 | G | 2.52 | 1.45 | 89.9 |
| 351 | A | 1.00 | -0.81 | 19.4 |
| 352 | G | 2.31 | 1.14 | 86.7 |
| 353 | N | 1.07 | -0.71 | 24.6 |
| 354 | H | 3.59 | 3.07 | 98.8 |

| resid (GcoA) | AA (GcoA) | relative entropy | z-score | percentile |
|---|---|---|---|---|
| 355 | A | 1.31 | -0.34 | 45.5 |
| 356 | C | 4.79 | 4.86 | 100.0 |
| 357 | A | 1.40 | -0.22 | 49.4 |
| 358 | G | 2.55 | 1.51 | 91.4 |
| 359 | I | 1.09 | -0.68 | 26.3 |
| 360 | Y | 2.01 | 0.69 | 79.6 |
| 361 | F | 1.33 | -0.31 | 47.2 |
| 362 | A | 1.65 | 0.16 | 63.4 |
| 363 | N | 1.71 | 0.24 | 65.8 |
| 364 | H | 0.88 | -1.00 | 13.8 |
| 365 | V | 1.49 | -0.08 | 56.3 |
| 366 | M | 1.24 | -0.45 | 40.8 |
| 367 | R | 1.30 | -0.36 | 45.0 |
| 368 | I | 1.95 | 0.61 | 77.4 |
| 369 | A | 1.20 | -0.51 | 37.1 |
| 370 | L | 1.86 | 0.48 | 74.4 |
| 371 | E | 1.28 | -0.40 | 42.3 |
| 372 | E | 1.21 | -0.50 | 37.8 |
| 373 | L | 1.80 | 0.38 | 70.8 |
| 374 | F | 2.07 | 0.79 | 80.8 |
| 375 | E | 1.06 | -0.73 | 23.3 |
| 376 | A | 1.05 | -0.75 | 22.4 |
| 377 | I | 1.58 | 0.06 | 59.5 |
| 378 | P | 2.18 | 0.95 | 84.0 |
| 379 | N | 1.85 | 0.46 | 73.7 |
| 380 | L | 1.79 | 0.37 | 70.5 |
| 381 | E | 1.16 | -0.58 | 32.7 |
| 382 | R | 1.21 | -0.49 | 38.6 |
| 383 | D | 1.21 | -0.50 | 37.6 |
| 384 | T | 0.87 | -1.01 | 13.3 |
| 385 | R | 0.59 | -1.43 | 1.5 |
| 386 | E | 0.68 | -1.29 | 3.9 |
| 387 | G | 0.63 | -1.36 | 2.9 |
| 388 | V | 1.20 | -0.52 | 36.6 |
| 389 | E | 0.58 | -1.44 | 1.2 |
| 390 | F | 1.61 | 0.10 | 61.2 |
| 391 | W | 1.46 | -0.13 | 53.6 |
| 392 | G | 2.59 | 1.56 | 92.4 |
| 393 | W | 3.14 | 2.39 | 97.1 |
| 394 | G | 1.07 | -0.71 | 25.1 |
| 395 | F | 3.03 | 2.23 | 96.6 |
| 396 | R | 2.55 | 1.51 | 91.2 |
| 397 | G | 1.97 | 0.63 | 78.1 |
| 398 | P | 1.72 | 0.26 | 66.3 |
| 399 | T | 1.08 | -0.69 | 26.0 |
| 400 | S | 0.72 | -1.24 | 5.4 |
| 401 | L | 2.14 | 0.89 | 82.8 |
| 402 | H | 1.63 | 0.12 | 62.4 |
| 403 | V | 1.70 | 0.24 | 65.6 |
| 404 | T | 0.95 | -0.89 | 16.7 |
| 405 | W | 2.59 | 1.56 | 92.1 |
| 406 | E | 1.11 | -0.64 | 30.0 |
| 407 | V | 1.08 | -0.70 | 25.6 |

Table S8. *Pseudomonas putida* strains used in this study.

| Strain ID | Genotype | Description of strain construction |
|---|---|---|
| CJ025 | KT2440 Δ*catA2* | *catA2* was deleted from *P. putida* KT2440 using pCJ004 and this deletion was confirmed by diagnostic colony PCR amplification of a 2,089 bp product with primer pair oCJ084/oCJ085. |
| CJ028 | KT2440 Δ*catBCA*::*Ptac*:*xylE* Δ*catA2* | *catBCA* was replaced in CJ025 with Ptac:*xylE* using pCJ005 and this gene replacement was confirmed by diagnostic colony PCR amplification of a 3,078 bp product with primer pair oCJ086/oCJ087. |
| AM140 | KT2440 Δ*catBCA*::*Ptac*:*xylE* Δ*catA2* Δ*pcaHG* | *pcaHG* was deleted by transforming CJ028 with pCJ011. The gene replacement was confirmed by amplification of a 2049 bp fragment using primers oCJ106/oCJ107. |
| AM142 | KT2440 Δ*catBCA*::*Ptac*:*xylE* Δ*catA2* Δ*pcaHG* / pBTL-*2* | AM140 transformed with pBTL-2 |
| AM144 | KT2440 Δ*catBCA*::*Ptac*:*xylE* Δ*catA2* Δ*pcaHG* / pCJ021 (*gcoAB* in pBTL-2) | AM140 transformed with pCJ021 |
| AM148 | KT2440 Δ*catBCA*::*Ptac*:*xylE* Δ*catA2* Δ*pcaHG* / p0AM27 ($gcoAB_{F169A}$ in pBTL-2) | AM140 transformed with pAM027 |
| CJ612 | KT2440 Δ*catBCA* Δ*catA2* | *catBCA* was deleted from CJ025 by transforming CJ025 with pCJ105. The gene deletion was confirmed by amplification of a 2407 bp product using primers oCJ086/oCJ087. |
| AM154 | KT2440 Ptac:*pcaHG* Δ*catBCA* Δ*catA2* | Native promoter of *pcaHG* replaced with Ptac by transforming CJ612 with pCJ020. The gene replacement was confirmed by amplification of an 1201 bp fragment using primers oAM127/oCJ135. |
| AM155 | KT2440 Ptac:*pcaHG* Δ*catBCA* Δ*catA2* / pBTL-2 | AM154 transformed with pBTL-2 |
| AM156 | KT2440 Ptac:*pcaHG* Δ*catBCA* Δ*catA2* / pCJ021 (*gcoAB* in pBTL-2) | AM154 transformed with pCJ021 |
| AM157 | KT2440 Ptac:*pcaHG* Δ*catBCA* Δ*catA2* / ($gcoAB_{F169A}$ in pBTL-2) | AM154 transformed with pAM027 |

Table S9. Plasmids used in this study.

| Plasmid ID | Use | Description of strain construction |
|---|---|---|
| pBTL-2 | Empty vector | pBTL-2 was described previously in (55) |
| pCJ004 | Deletion of *catA2* in *P. putida* KT2440 | Construction of pCJ004 was described previously in Johnson and Beckham (12). |
| pCJ005 | Replacing *catBCA* with Ptac:*xylE* in *P. putida* KT2440 | The 5' targeting region was amplified from *P. putida* KT2440 genomic DNA with primer pair oCJ042/oCJ043 (1,104 bp, which incorporated the tac promoter), *xylE* (969 bp) was amplified from *P. putida* mt-2 (ATCC 23973) genomic DNA with primer pair oCJ044/oCJ045, and the 3' targeting region was amplified using primer pair oCJ046/oCJ047 (1033 bp). These fragments were then assembled into pCM433 digested with AatII and SacI (7991 bp). |
| pCJ011 | Deletion of *pcaHG* in *P. putida* KT2440 | Construction of pCJ011 was described previously in Johnson and Beckham (12). |
| pCJ020 | Insertion of tac promoter upstream of *pcaHG* in *P. putida* KT2440 | Construction of pCJ020 was described previously in Johnson and Beckham (12). |
| pCJ021 | Episomal expression of *gcoAB* on pBTL-2 | Construction of pCJ021 was described previously in Tumen-Velasquez, *et al.* (56). |
| pCJ105 | Deletion of *catBCA* in *P. putida* KT2440 | The 5' (1054 bp) and 3' (1044 bp) targeting regions were amplifed from pCJ005 with primers oCJ542/oCJ543 and oCJ544/oCJ545, respectively, and assembled into pK18sB (Genbank MH166772 (57)) digested with EcoRI and HindII. |
| pAM027 | Episomal expression of $gcoAB_{F169A}$ on pBTL-2 | pCJ021 was linearly amplified using oAM173 and oAM174 to introduce the gcoA F169 mutation by site-directed mutagenesis and treated with NEB KLD Enzyme Mix (New England Biolabs), which includes kinase, ligase, and DpnI enzymes to phosphorylate and circularize the PCR product and digest the template, according to the manufacturer's instructions. |
| pGcoA-F196A, pGcoA-F196H, pGcoA-F196I, pGcoA-F196L, pGcoA-F196S, pGcoA-F196V | Expression of GcoA containing F196A, F196H, F196I, F196L, F196S, or F196V mutations | pCJ047 (pGEX-6P-1 vector containing WT GcoA (1)) was linearly amplified using forward primers F196A, F196H, F196I, F196L, F196S, or F196V and F169rev to introduce the various gcoA mutations by site-directed |

| | | |
|---|---|---|
| | | mutagenesis and treated with NEB KLD Enzyme Mix (New England Biolabs), which includes kinase, ligase, and DpnI enzymes to phosphorylate and circularize the PCR product and digest the template, according to the manufacturer's instructions. |

Table S10. Sequences of DNA oligos used in this study.

| | |
|---|---|
| F169rev | AGCTTGGCGAACCACTCG |
| F169A | GAACCGCTCGgcCACCAACGCC |
| F169H | GAACCGCTCGcaCACCAACGCC |
| F169I | GAACCGCTCGaTCACCAACGC |
| F169L | GAACCGCTCGcTCACCAACGC |
| F169S | GAACCGCTCGTcCACCAACGC |
| F169V | GAACCGCTCGgTCACCAACGC |
| oCJ042 | ccgaaaagtgccacctGACGTCcctgttgctcgatcaacgc |
| oCJ043 | tcataAGATCTctcctgtgtgaaattgttatccgctcacaattccacacattatacgagccgatgattaattgtcaacagctctgttgccaggtcccgtc |
| oCJ044 | aggagAGATCTtatgaacaaaggtgtaatgcgacc |
| oCJ045 | cgaacGCGGCCGCgcaataagtcgtaccggaccatc |
| oCJ046 | attgcGCGGCCGCgttcgaggttatgtcactgtgattttg |
| oCJ047 | gctggatcctctagtGAGCTCcgcctgctccaggttg |
| oCJ084 | CCTCAATGGCTTTGCCAG |
| oCJ085 | GTACAACACACTGCCAGC |
| oCJ086 | TGTGGGCATGGTGTGTTC |
| oCJ087 | TCTTCAAAGCGTCCGGTG |
| oCJ106 | ATCTTGAACCAACGCACC |
| oCJ107 | CACAAGGCAATCCTGATCG |
| oCJ135 | AGGCTGATGTTGATGTGC |
| oCJ542 | aggaaacagctatgacatgattacGAATTCcctgttgctcgatcaacgccag |
| oCJ543 | cgaacGCGGCCGCtgttgccaggtcccgtcagg |
| oCJ544 | gacgggacctggcaacaGCGGCCGCgttcgag |
| oCJ545 | cgttgtaaaacgacggccagtgccAAGCTTcgcctgctccaggttgaatgc |
| oAM127 | GAGCTGTTGACAATTAATCATCGGC |
| oAM173 | GAACCGCTCGgcCACCAACGCC |
| oAM174 | AGCTTGGCGAACCACTCG |

**Movie S1. Motion of active site Phe residues in WT GcoA with guaiacol bound.** 80 ns MD simulation demonstrates the relative stability of F169 (center), F75 (left), and F395 (right). Compound I heme is shown in sticks along with Fe molecule (orange) and bound oxygen atom (red) in sphere representation.

**Movie S2. Motion of active site F residues in WT GcoA with syringol bound.** 80 ns MD simulation reveals the increased flexibility of F75 (left), F395 (right), and especially F169 (center). Compound I heme is shown in sticks along with Fe molecule (orange) and bound oxygen atom (red) in sphere representation.

**Movie S3. Substrate access loop is relatively stable in WT GcoA with guaiacol bound.** 80 ns MD simulation reveals a relatively stable substrate access loop (residues 154-210, shown in raspberry color) when guaiacol (green "sticks" representation) is bound at the active site. Compound I heme is shown in sticks along with Fe molecule (orange) and bound oxygen atom (red) in sphere representation.

**Movie S4. Substrate access loop is flexible in WT GcoA with syringol bound.** 80 ns MD simulation reveals a more active substrate access loop (residues 154-210, shown in raspberry color) when syringol (pink "sticks" representation) is bound at the active site. Compound I heme is shown in sticks along with Fe molecule (orange) and bound oxygen atom (red) in sphere representation.

*DFT optimized geometries*

Electronic energies (E), zero point energy (ZPE), free energy (G(T)), quasiharmonic corrected free energy (qh-G(T)), and electronic energy from high level single point calculation (E Single point) of all stationary points (in a.u.). Cartesian coordinates are reported in xyz format.

| Structure | E (au) | ZPE (au) | G(T) (au) | qh-G(T) (au) | E Single Point (au) |
|---|---|---|---|---|---|
| 1 - syringol | -536.495040 | 0.170476 | -536.361137 | -536.360232 | -536.706360 |
| Fe=O-Porph - doublet | -1625.106262 | 0.317660 | -1624.839694 | -1624.837490 | -1625.567736 |
| Fe=O-Porph - quartet | -1625.106093 | 0.317696 | -1624.840115 | -1624.837883 | -1625.567464 |
| FeH2O-Porph - doublet | -1626.387773 | 0.338497 | -1626.101953 | -1626.099515 | -1626.878338 |
| 3 - hemiacetal | -611.711120 | 0.175635 | -611.573661 | -611.572350 | -611.959659 |
| 7 - acetal | -535.284576 | 0.149181 | -535.170634 | -535.169544 | -535.479338 |
| Fe-Porph - sextet | -1549.964112 | 0.312990 | -1549.704649 | -1549.700465 | -1550.402389 |
| Fe=O-Porph - doublet + Syringol (reactant complex) | -2161.610445 | 0.489091 | -2161.193023 | -2161.181629 | -2162.291362 |
| Fe=O-Porph - quartet + Syringol (reactant complex) | -2161.610252 | 0.489162 | -2161.193304 | -2161.181929 | -2162.290915 |
| TS1-a - doublet | -2161.574263 | 0.482285 | -2161.159708 | -2161.150955 | -2162.264394 |
| TS1-b - doublet | -2161.578885 | 0.482284 | -2161.163139 | -2161.155284 | -2162.269456 |
| TS1-a - quartet | -2161.572822 | 0.482653 | -2161.160202 | -2161.149881 | -2162.260628 |
| TS1-b - quartet | -2161.578118 | 0.482402 | -2161.163372 | -2161.155008 | -2162.268868 |
| Int1-b - doublet | -2161.595046 | 0.484420 | -2161.180622 | -2161.170366 | -2162.290290 |
| Int1-a - quartet | -2161.585020 | 0.485263 | -2161.172652 | -2161.160884 | -2162.274849 |
| Int1-b - quartet | -2161.593829 | 0.485312 | -2161.178962 | -2161.168993 | -2162.286346 |
| TS2-b - quartet | -2161.590346 | 0.480888 | -2161.177325 | -2161.169410 | -2162.289260 |
| TS2-a - quartet | -2161.579121 | 0.484609 | -2161.165749 | -2161.154995 | -2162.270819 |
| TS1-c - doublet | -2161.598186 | 0.483285 | -2161.182677 | -2161.174168 | -2162.287930 |
| TS1-c - quartet | -2161.599963 | 0.483375 | -2161.184856 | -2161.176470 | -2162.289148 |
| Int1-c - quartet | -2161.623252 | 0.487968 | -2161.207527 | -2161.195638 | -2162.309777 |
| TS2-c - doublet | -2161.593206 | 0.481997 | -2161.180272 | -2161.170068 | -2162.282252 |
| TS2-c - quartet | -2161.589202 | 0.481568 | -2161.176370 | -2161.167267 | -2162.279422 |

*DFT Cartesian coordinates of optimized structures*

1 - syringol

| | | | |
|---|---|---|---|
| H | 1.39112 | -2.28267 | 1.27967 |
| C | 1.55655 | -3.16232 | 0.65137 |
| O | 1.83943 | -2.80032 | -0.70216 |
| H | 0.64644 | -3.76575 | 0.61463 |
| H | 2.37296 | -3.76518 | 1.07192 |
| C | 3.00432 | -2.10863 | -0.92089 |
| C | 3.94560 | -2.65330 | -1.80389 |
| C | 3.24620 | -0.85454 | -0.34585 |
| C | 5.11402 | -1.96235 | -2.10854 |
| H | 3.72774 | -3.62174 | -2.24238 |
| C | 4.44085 | -0.17289 | -0.64837 |
| O | 2.33108 | -0.28387 | 0.49369 |
| C | 5.37564 | -0.71604 | -1.52678 |
| H | 5.83572 | -2.39322 | -2.79606 |
| H | 6.29099 | -0.18504 | -1.76219 |
| O | 4.54423 | 1.04142 | -0.00637 |
| C | 5.68200 | 1.84488 | -0.27327 |
| H | 5.55607 | 2.75526 | 0.31574 |
| H | 6.60729 | 1.33912 | 0.03158 |
| H | 5.74538 | 2.10416 | -1.33793 |
| H | 2.70407 | 0.57525 | 0.76038 |

Fe=O-Porph - doublet

| | | | |
|---|---|---|---|
| Fe | 0.09001 | 0.04213 | -0.36230 |
| N | 1.85561 | -0.91507 | -0.14137 |
| N | -0.85946 | -1.74466 | -0.26081 |
| N | 1.02135 | 1.81096 | -0.13520 |
| N | -1.69352 | 0.98033 | -0.29018 |
| C | 3.09603 | -0.33521 | -0.04630 |
| C | -2.21650 | -1.96098 | -0.33048 |
| C | 2.07516 | -2.26630 | -0.20461 |
| C | -0.27579 | -2.99282 | -0.30557 |
| C | 2.37035 | 2.02263 | -0.03347 |
| C | -2.93239 | 0.40160 | -0.37143 |
| C | 0.44628 | 3.05350 | -0.17221 |
| C | -1.90782 | 2.33688 | -0.31952 |
| C | 4.12324 | -1.34905 | -0.03748 |
| C | -2.49372 | -3.37588 | -0.38397 |
| C | 3.48944 | -2.54821 | -0.14220 |
| C | -1.29180 | -4.01358 | -0.37375 |
| C | 2.65560 | 3.43696 | 0.00387 |
| C | -3.95735 | 1.41528 | -0.43884 |
| C | 1.45873 | 4.07841 | -0.08606 |
| C | -3.32040 | 2.61780 | -0.40765 |
| H | 5.18468 | -1.14989 | 0.03703 |
| H | -3.48552 | -3.80699 | -0.43432 |
| H | 3.92107 | -3.54060 | -0.16883 |
| H | -1.09269 | -5.07702 | -0.41026 |

| | | | |
|---|---|---|---|
| H | 3.64697 | 3.86392 | 0.08620 |
| H | -5.01933 | 1.21486 | -0.50274 |
| H | 1.26084 | 5.14271 | -0.09160 |
| H | -3.75032 | 3.61082 | -0.44037 |
| C | 3.34041 | 1.02846 | 0.01904 |
| C | -3.18338 | -0.96714 | -0.37529 |
| C | -0.91718 | 3.30603 | -0.26237 |
| C | 1.08664 | -3.23934 | -0.29289 |
| H | 4.37546 | 1.34644 | 0.09875 |
| H | -4.22017 | -1.28302 | -0.44097 |
| H | -1.23209 | 4.34481 | -0.28732 |
| H | 1.40798 | -4.27526 | -0.33853 |
| O | 0.16134 | 0.05021 | -1.98164 |
| S | -0.02287 | -0.39701 | 2.22638 |
| C | -1.70575 | -0.05646 | 2.81715 |
| H | -2.40211 | -0.74305 | 2.31979 |
| H | -1.75557 | -0.23660 | 3.89501 |
| H | -2.01065 | 0.96742 | 2.58591 |

Fe=O-Porph - quartet

| | | | |
|---|---|---|---|
| Fe | 0.08895 | 0.03952 | -0.36576 |
| N | 1.86276 | -0.90115 | -0.14522 |
| N | -0.84332 | -1.75056 | -0.26510 |
| N | 1.00580 | 1.81862 | -0.12816 |
| N | -1.70236 | 0.96435 | -0.29294 |
| C | 3.09813 | -0.31000 | -0.04804 |
| C | -2.19942 | -1.97867 | -0.33111 |
| C | 2.09490 | -2.25008 | -0.20789 |
| C | -0.24962 | -2.99497 | -0.30398 |
| C | 2.35278 | 2.04153 | -0.02887 |
| C | -2.93637 | 0.37652 | -0.37754 |
| C | 0.42013 | 3.05592 | -0.16389 |
| C | -1.92741 | 2.31953 | -0.31803 |
| C | 4.13428 | -1.31466 | -0.03929 |
| C | -2.46453 | -3.39574 | -0.37792 |
| C | 3.51148 | -2.51948 | -0.14488 |
| C | -1.25745 | -4.02362 | -0.36644 |
| C | 2.62631 | 3.45830 | 0.00914 |
| C | -3.96936 | 1.38196 | -0.44505 |
| C | 1.42390 | 4.08957 | -0.07820 |
| C | -3.34204 | 2.58935 | -0.40862 |
| H | 5.19381 | -1.10596 | 0.03651 |
| H | -3.45284 | -3.83511 | -0.42546 |
| H | 3.95197 | -3.50797 | -0.17119 |
| H | -1.04971 | -5.08554 | -0.39877 |
| H | 3.61423 | 3.89349 | 0.08978 |
| H | -5.02956 | 1.17322 | -0.51164 |
| H | 1.21688 | 5.15213 | -0.08305 |
| H | -3.77974 | 3.57904 | -0.43907 |
| C | 3.33110 | 1.05529 | 0.02080 |
| C | -3.17533 | -0.99432 | -0.38054 |
| C | -0.94542 | 3.29684 | -0.25451 |
| C | 1.11433 | -3.23131 | -0.29390 |
| H | 4.36354 | 1.38158 | 0.10089 |

| | | | |
|---|---|---|---|
| H | -4.20904 | -1.32004 | -0.44639 |
| H | -1.26930 | 4.33292 | -0.27674 |
| H | 1.44343 | -4.26486 | -0.33763 |
| O | 0.17228 | 0.08273 | -1.98469 |
| S | -0.02234 | -0.40009 | 2.22592 |
| C | -1.70860 | -0.07328 | 2.81526 |
| H | -2.39975 | -0.76382 | 2.31616 |
| H | -1.75761 | -0.25730 | 3.89258 |
| H | -2.02090 | 0.94912 | 2.58760 |

Fe-Porph-H2O - doublet

| | | | |
|---|---|---|---|
| Fe | 0.05972 | 0.00013 | -0.10196 |
| N | 1.52154 | -1.39355 | -0.16026 |
| N | -1.35210 | -1.42758 | -0.24699 |
| N | 1.48798 | 1.42930 | -0.14419 |
| N | -1.38396 | 1.39301 | -0.26691 |
| C | 2.88049 | -1.19649 | -0.11935 |
| C | -2.71273 | -1.26488 | -0.35222 |
| C | 1.33516 | -2.75365 | -0.11963 |
| C | -1.13438 | -2.78246 | -0.20834 |
| C | 2.84999 | 1.26525 | -0.10808 |
| C | -2.74203 | 1.19720 | -0.36684 |
| C | 1.26887 | 2.78338 | -0.11069 |
| C | -1.19962 | 2.75389 | -0.22468 |
| C | 3.56891 | -2.46469 | -0.07150 |
| C | -3.36949 | -2.55028 | -0.37862 |
| C | 2.61021 | -3.43062 | -0.06861 |
| C | -2.39038 | -3.49155 | -0.28494 |
| C | 3.50874 | 2.55002 | -0.06016 |
| C | -3.42848 | 2.46610 | -0.39609 |
| C | 2.52745 | 3.49217 | -0.05877 |
| C | -2.47205 | 3.43125 | -0.30342 |
| H | 4.64489 | -2.58133 | -0.03836 |
| H | -4.43967 | -2.69494 | -0.45811 |
| H | 2.73599 | -4.50556 | -0.03417 |
| H | -2.49058 | -4.56969 | -0.27351 |
| H | 4.58169 | 2.69211 | -0.02841 |
| H | -4.50190 | 2.58524 | -0.47434 |
| H | 2.62670 | 4.56997 | -0.02729 |
| H | -2.59898 | 4.50658 | -0.29181 |
| C | 3.50881 | 0.04236 | -0.10607 |
| C | -3.36883 | -0.04179 | -0.41789 |
| C | 0.02654 | 3.40280 | -0.14383 |
| C | 0.10743 | -3.40215 | -0.13942 |
| H | 4.59388 | 0.05546 | -0.06899 |
| H | -4.45176 | -0.05500 | -0.49653 |
| H | 0.01191 | 4.48831 | -0.11591 |
| H | 0.11893 | -4.48766 | -0.11005 |
| O | 0.14625 | -0.00294 | -2.28515 |
| S | 0.09768 | -0.00434 | 2.11918 |
| C | -1.62246 | 0.00586 | 2.73814 |
| H | -2.17234 | -0.87980 | 2.40992 |
| H | -1.56172 | -0.00070 | 3.83151 |
| H | -2.15871 | 0.90343 | 2.41984 |

| | | | |
|---|---|---|---|
| H | 0.93595 | -0.21781 | -2.80169 |
| H | -0.58708 | 0.20225 | -2.88235 |

3 - hemiacetal

| | | | |
|---|---|---|---|
| H | 1.47660 | -2.15525 | 1.29974 |
| C | 1.65474 | -3.08055 | 0.74435 |
| O | 1.89375 | -2.82733 | -0.64181 |
| H | 0.76319 | -3.71086 | 0.78355 |
| H | 2.49942 | -3.62123 | 1.19285 |
| C | 3.02799 | -2.11676 | -0.94605 |
| C | 3.98867 | -2.71084 | -1.77105 |
| C | 3.22349 | -0.79990 | -0.50240 |
| C | 5.12895 | -2.00881 | -2.15682 |
| H | 3.80864 | -3.72736 | -2.10610 |
| C | 4.38488 | -0.10993 | -0.88963 |
| O | 2.29480 | -0.18985 | 0.29202 |
| C | 5.33803 | -0.69771 | -1.71889 |
| H | 5.86142 | -2.47956 | -2.80567 |
| H | 6.20470 | -0.12883 | -2.03365 |
| O | 4.44792 | 1.18592 | -0.38917 |
| C | 5.72711 | 1.75426 | -0.15307 |
| H | 5.53348 | 2.56952 | 0.55470 |
| H | 6.39884 | 1.01182 | 0.28822 |
| H | 2.64998 | 0.69606 | 0.48749 |
| O | 6.36026 | 2.20627 | -1.31420 |
| H | 5.78788 | 2.87382 | -1.72693 |

7 - acetal

| | | | |
|---|---|---|---|
| H | 0.75901 | -2.13039 | 0.20764 |
| C | 1.37443 | -2.94185 | 0.61315 |
| O | 2.41466 | -3.30572 | -0.29130 |
| H | 0.76365 | -3.83940 | 0.72998 |
| H | 1.77222 | -2.63702 | 1.58600 |
| C | 3.30561 | -2.35851 | -0.69474 |
| C | 4.16238 | -2.71603 | -1.75861 |
| C | 3.47947 | -1.08234 | -0.16486 |
| C | 5.13019 | -1.84415 | -2.24549 |
| H | 4.03282 | -3.70405 | -2.18811 |
| C | 4.45089 | -0.21884 | -0.67160 |
| O | 2.83799 | -0.47001 | 0.90347 |
| C | 5.29943 | -0.55616 | -1.70747 |
| H | 5.76328 | -2.16355 | -3.06797 |
| H | 6.04758 | 0.13582 | -2.07631 |
| O | 4.43425 | 0.95661 | 0.04740 |
| C | 3.24772 | 0.89673 | 0.83689 |
| H | 3.46062 | 1.25930 | 1.84528 |
| H | 2.45740 | 1.49707 | 0.35901 |

Fe=O-Porph - doublet + Syringol (reactant complex)

| | | | |
|---|---|---|---|
| Fe | 1.66063 | 0.05527 | -0.03921 |
| N | 3.04911 | -1.39611 | -0.26839 |
| N | 0.35785 | -1.33879 | 0.64003 |
| N | 3.06410 | 1.45172 | -0.40051 |
| N | 0.35984 | 1.50567 | 0.47421 |
| C | 4.34704 | -1.22997 | -0.68429 |
| C | -0.94055 | -1.12329 | 1.04267 |
| C | 2.85094 | -2.74973 | -0.18366 |
| C | 0.51885 | -2.70836 | 0.60635 |
| C | 4.35884 | 1.23443 | -0.79035 |
| C | -0.93846 | 1.33919 | 0.87846 |
| C | 2.88645 | 2.80978 | -0.42212 |
| C | 0.53628 | 2.85969 | 0.31984 |
| C | 4.98544 | -2.51254 | -0.85571 |
| C | -1.60112 | -2.38078 | 1.29926 |
| C | 4.05466 | -3.45656 | -0.54959 |
| C | -0.69648 | -3.36056 | 1.02618 |
| C | 5.01973 | 2.48868 | -1.05834 |
| C | -1.59806 | 2.61726 | 0.99244 |
| C | 4.10287 | 3.46883 | -0.83131 |
| C | -0.68062 | 3.56200 | 0.64812 |
| H | 6.01176 | -2.65249 | -1.17037 |
| H | -2.63265 | -2.47640 | 1.61194 |
| H | 4.15720 | -4.53420 | -0.55777 |
| H | -0.82471 | -4.43350 | 1.09393 |
| H | 6.04946 | 2.58691 | -1.37760 |
| H | -2.63204 | 2.74888 | 1.28294 |
| H | 4.22212 | 4.54078 | -0.92438 |
| H | -0.80016 | 4.63732 | 0.60907 |
| C | 4.96445 | -0.01029 | -0.91712 |
| C | -1.54636 | 0.11921 | 1.15894 |
| C | 1.71106 | 3.47247 | -0.08954 |
| C | 1.67137 | -3.36781 | 0.21454 |
| H | 6.00159 | -0.02923 | -1.23771 |
| H | -2.58719 | 0.13924 | 1.46416 |
| H | 1.71210 | 4.55606 | -0.15834 |
| H | 1.65748 | -4.45318 | 0.23290 |
| O | 1.14036 | -0.02621 | -1.57519 |
| S | 2.36987 | -0.20717 | 2.46738 |
| C | 1.16959 | 0.65749 | 3.52049 |
| H | 0.18231 | 0.19813 | 3.38687 |
| H | 1.46207 | 0.54653 | 4.56872 |
| H | 1.09435 | 1.71498 | 3.25440 |
| H | -1.07718 | -0.37293 | -2.57255 |
| C | -1.79496 | -1.05523 | -3.03990 |
| O | -2.85649 | -1.38660 | -2.14522 |
| H | -1.29448 | -1.99801 | -3.26607 |
| H | -2.18822 | -0.62208 | -3.96832 |
| C | -3.66396 | -0.38021 | -1.68442 |
| C | -3.65525 | 0.94760 | -2.11738 |
| C | -4.55592 | -0.80526 | -0.68591 |
| C | -4.55721 | 1.84644 | -1.54559 |
| H | -2.95673 | 1.27826 | -2.87683 |
| C | -5.45808 | 0.10746 | -0.11835 |
| O | -4.53653 | -2.10136 | -0.25399 |
| C | -5.45552 | 1.43972 | -0.55644 |
| H | -4.56185 | 2.88077 | -1.87671 |
| H | -3.83051 | -2.53793 | -0.76524 |

| | | | |
|---|---|---|---|
| H | -6.15037 | 2.15606 | -0.13398 |
| O | -6.27786 | -0.40292 | 0.84856 |
| C | -7.23509 | 0.46360 | 1.42823 |
| H | -7.78763 | -0.14083 | 2.15039 |
| H | -6.75983 | 1.30557 | 1.95102 |
| H | -7.93302 | 0.85863 | 0.67740 |

Fe=O-Porph - quartet + Syringol (reactant complex)

| | | | |
|---|---|---|---|
| Fe | 1.61447 | 0.06766 | -0.02681 |
| N | 3.11789 | -1.24448 | -0.34701 |
| N | 0.45737 | -1.45705 | 0.61792 |
| N | 2.88746 | 1.59800 | -0.34715 |
| N | 0.20516 | 1.37728 | 0.57368 |
| C | 4.38804 | -0.94746 | -0.77659 |
| C | -0.84092 | -1.37194 | 1.06885 |
| C | 3.03974 | -2.61245 | -0.32033 |
| C | 0.73655 | -2.80564 | 0.52723 |
| C | 4.18672 | 1.51103 | -0.77005 |
| C | -1.06465 | 1.08300 | 0.99472 |
| C | 2.59109 | 2.93431 | -0.30970 |
| C | 0.26027 | 2.74684 | 0.47088 |
| C | 5.13058 | -2.16075 | -1.01823 |
| C | -1.37820 | -2.69136 | 1.29833 |
| C | 4.29134 | -3.19502 | -0.74015 |
| C | -0.40184 | -3.57719 | 0.95881 |
| C | 4.72990 | 2.82839 | -0.99756 |
| C | -1.83123 | 2.29298 | 1.16833 |
| C | 3.73609 | 3.71407 | -0.71337 |
| C | -1.00664 | 3.32660 | 0.84635 |
| H | 6.15836 | -2.19666 | -1.35635 |
| H | -2.38209 | -2.88634 | 1.65179 |
| H | 4.48632 | -4.25820 | -0.79941 |
| H | -0.43287 | -4.65894 | 0.99052 |
| H | 5.73971 | 3.02952 | -1.33186 |
| H | -2.86751 | 2.32294 | 1.47809 |
| H | 3.75892 | 4.79519 | -0.76452 |
| H | -1.22056 | 4.38787 | 0.85043 |
| C | 4.89413 | 0.33007 | -0.96266 |
| C | -1.55446 | -0.19547 | 1.24357 |
| C | 1.36882 | 3.47658 | 0.06952 |
| C | 1.92828 | -3.34669 | 0.07656 |
| H | 5.92218 | 0.41484 | -1.30139 |
| H | -2.58363 | -0.28399 | 1.57467 |
| H | 1.27346 | 4.55791 | 0.04574 |
| H | 2.00776 | -4.42902 | 0.04899 |
| O | 1.08710 | 0.03453 | -1.56267 |
| S | 2.40565 | -0.23114 | 2.45270 |
| C | 1.16332 | 0.48786 | 3.56521 |
| H | 0.21514 | -0.04881 | 3.43670 |
| H | 1.49131 | 0.36242 | 4.60133 |
| H | 0.99259 | 1.54446 | 3.34371 |
| H | -1.06081 | -0.27475 | -2.73086 |
| C | -1.74051 | -1.03195 | -3.13502 |
| O | -2.75218 | -1.37532 | -2.18921 |
| H | -1.18116 | -1.95098 | -3.31617 |
| H | -2.18687 | -0.68750 | -4.07653 |

| | | | |
|---|---|---|---|
| C | -3.60507 | -0.39371 | -1.75724 |
| C | -3.71526 | 0.89441 | -2.28574 |
| C | -4.41105 | -0.80029 | -0.68160 |
| C | -4.65046 | 1.77013 | -1.73212 |
| H | -3.08332 | 1.21244 | -3.10651 |
| C | -5.34429 | 0.09034 | -0.13037 |
| O | -4.27296 | -2.05380 | -0.15528 |
| C | -5.46430 | 1.38052 | -0.66608 |
| H | -4.74768 | 2.77302 | -2.13740 |
| H | -3.55897 | -2.47679 | -0.66713 |
| H | -6.18579 | 2.07843 | -0.25766 |
| O | -6.06478 | -0.39591 | 0.92507 |
| C | -7.05489 | 0.44260 | 1.49057 |
| H | -7.51851 | -0.13837 | 2.29041 |
| H | -6.62045 | 1.35864 | 1.91526 |
| H | -7.82096 | 0.72046 | 0.75386 |

TS1-a - doublet

| | | | |
|---|---|---|---|
| Fe | 1.40144 | -0.00970 | -0.13820 |
| N | 0.02879 | 1.02247 | -1.18756 |
| N | 0.58529 | -1.74916 | -0.74189 |
| N | 2.33774 | 1.72212 | 0.30115 |
| N | 2.87407 | -1.04991 | 0.80041 |
| C | -0.01668 | 2.37511 | -1.40464 |
| C | 0.99263 | -3.01496 | -0.41623 |
| C | -1.05845 | 0.50016 | -1.83663 |
| C | -0.59229 | -1.89416 | -1.43142 |
| C | 1.98873 | 2.98792 | -0.10102 |
| C | 2.98946 | -2.41204 | 0.91115 |
| C | 3.42523 | 1.87082 | 1.12441 |
| C | 3.88234 | -0.51795 | 1.56627 |
| C | -1.17227 | 2.71889 | -2.19986 |
| C | 0.06442 | -3.99007 | -0.93829 |
| C | -1.82699 | 1.55280 | -2.46088 |
| C | -0.92299 | -3.29322 | -1.56522 |
| C | 2.88371 | 3.96031 | 0.47790 |
| C | 4.11719 | -2.75157 | 1.75011 |
| C | 3.76823 | 3.26761 | 1.24733 |
| C | 4.66516 | -1.57706 | 2.16244 |
| H | -1.42970 | 3.72268 | -2.51415 |
| H | 0.16470 | -5.06182 | -0.82203 |
| H | -2.73714 | 1.40007 | -3.02637 |
| H | -1.80134 | -3.67365 | -2.07094 |
| H | 2.82563 | 5.02867 | 0.31242 |
| H | 4.42627 | -3.76110 | 1.99008 |
| H | 4.58905 | 3.65034 | 1.84054 |
| H | 5.52079 | -1.42245 | 2.80756 |
| C | 0.90559 | 3.29508 | -0.91577 |
| C | 2.12507 | -3.33147 | 0.33108 |
| C | 4.12949 | 0.83655 | 1.73307 |
| C | -1.36134 | -0.85381 | -1.93683 |
| H | 0.75001 | 4.34015 | -1.16685 |
| H | 2.32478 | -4.38431 | 0.50758 |
| H | 4.96658 | 1.11472 | 2.36642 |

| | | | |
|---|---|---|---|
| H | -2.26888 | -1.12019 | -2.47002 |
| O | 0.50962 | -0.06377 | 1.35876 |
| S | 3.07820 | -0.04244 | -1.97027 |
| C | 3.15592 | -1.70612 | -2.69886 |
| H | 2.18015 | -2.02106 | -3.07767 |
| H | 3.89369 | -1.70219 | -3.50665 |
| H | 3.47996 | -2.42484 | -1.93685 |
| H | -0.24219 | 0.81085 | 1.51956 |
| C | -1.15170 | 1.77408 | 1.85790 |
| O | -2.29284 | 1.65803 | 1.09011 |
| H | -0.65694 | 2.71174 | 1.61014 |
| H | -1.31360 | 1.61176 | 2.92754 |
| C | -3.20106 | 0.64803 | 1.32546 |
| C | -3.04553 | -0.37833 | 2.26000 |
| C | -4.33500 | 0.73029 | 0.50454 |
| C | -4.07139 | -1.31066 | 2.39468 |
| C | -5.36388 | -0.21681 | 0.65778 |
| O | -4.45886 | 1.70791 | -0.43539 |
| C | -5.22755 | -1.23155 | 1.61165 |
| H | -3.96870 | -2.11844 | 3.11294 |
| H | -3.61352 | 2.19199 | -0.45190 |
| H | -6.01085 | -1.96946 | 1.73898 |
| O | -6.43467 | -0.05314 | -0.17285 |
| C | -7.51111 | -0.96422 | -0.04985 |
| H | -8.25381 | -0.65082 | -0.78621 |
| H | -7.19872 | -1.99507 | -0.26721 |
| H | -7.95887 | -0.93072 | 0.95285 |
| H | -2.13264 | -0.46845 | 2.83620 |

TS1-b - doublet

| | | | |
|---|---|---|---|
| Fe | -1.17718 | 0.03369 | 0.21432 |
| N | -1.84250 | -1.87232 | 0.30080 |
| N | -2.76995 | 0.54082 | -0.93502 |
| N | 0.28371 | -0.43599 | 1.50345 |
| N | -0.59936 | 1.96692 | 0.22326 |
| C | -1.33882 | -2.90031 | 1.06155 |
| C | -3.06975 | 1.78091 | -1.44343 |
| C | -2.88845 | -2.40496 | -0.40830 |
| C | -3.68270 | -0.32850 | -1.48891 |
| C | 0.51560 | -1.64620 | 2.10434 |
| C | -1.17142 | 3.01305 | -0.44878 |
| C | 1.28206 | 0.39808 | 1.93498 |
| C | 0.53154 | 2.46974 | 0.81280 |
| C | -2.08204 | -4.11282 | 0.81951 |
| C | -4.22322 | 1.69977 | -2.30827 |
| C | -3.03712 | -3.80791 | -0.10201 |
| C | -4.59696 | 0.39192 | -2.34272 |
| C | 1.69003 | -1.57538 | 2.93825 |
| C | -0.39110 | 4.21497 | -0.26732 |
| C | 2.17240 | -0.30692 | 2.82452 |
| C | 0.67059 | 3.87527 | 0.51281 |
| H | -1.88542 | -5.06387 | 1.29793 |
| H | -4.66485 | 2.54099 | -2.82730 |
| H | -3.78971 | -4.45641 | -0.53227 |
| H | -5.41213 | -0.06128 | -2.89239 |

H 2.08576 -2.39874 3.51903
H -0.63296 5.17881 -0.69678
H 3.04461 0.12778 3.29541
H 1.48411 4.50098 0.85623
C -0.25191 -2.79291 1.91962
C -2.33771 2.93744 -1.20580
C 1.41101 1.74322 1.60354
C -3.72726 -1.69535 -1.26313
H 0.04232 -3.68302 2.46773
H -2.68032 3.85028 -1.68402
H 2.27395 2.26940 1.99780
H -4.50879 -2.25588 -1.76733
O -0.21242 -0.23033 -1.23582
S -2.86542 0.38497 1.97310
C -3.16202 2.16577 2.18607
H -3.56357 2.58649 1.25640
H -3.90438 2.30398 2.97768
H -2.23968 2.69651 2.43569
H 0.25191 -1.35735 -1.35615
C 1.01246 -2.36228 -1.69228
O 2.06628 -1.83710 -2.42130
H 0.38712 -2.97508 -2.34416
H 1.30924 -2.84448 -0.75518
C 3.17100 -1.38379 -1.70356
C 4.22630 -2.27174 -1.47436
C 3.22921 -0.03998 -1.30398
C 5.38231 -1.81580 -0.85452
H 4.12798 -3.29675 -1.81826
C 4.42258 0.40494 -0.67668
O 2.25391 0.86629 -1.51478
C 5.48280 -0.47617 -0.45966
H 6.21419 -2.49192 -0.68107
H 1.35086 0.44985 -1.54689
H 6.39049 -0.12969 0.02099
O 4.42584 1.72481 -0.31534
C 5.62683 2.26219 0.20305
H 5.43508 3.32498 0.36670
H 5.90766 1.79559 1.15845
H 6.46053 2.15047 -0.50367

TS1-a - quartet

Fe 1.55737 -0.16135 0.01966
N 1.29854 -0.83859 1.90931
N 1.54487 1.75177 0.74357
N 1.64730 -2.04379 -0.66328
N 2.01561 0.53148 -1.80577
C 1.16504 -2.14915 2.28634
C 1.68480 2.90022 0.00848
C 1.12493 -0.09202 3.05008
C 1.34278 2.15052 2.04335
C 1.45973 -3.18874 0.06453
C 2.07173 1.84702 -2.19592
C 1.87935 -2.44569 -1.95090
C 2.19242 -0.21184 -2.94488
C 0.91796 -2.23615 3.70537

C 1.56233 4.05761 0.86410
C 0.89247 -0.95987 4.17886
C 1.35112 3.59159 2.12667
C 1.56148 -4.34941 -0.79339
C 2.31837 1.93368 -3.61529
C 1.82369 -3.88814 -2.04446
C 2.39721 0.65628 -4.07930
H 0.78073 -3.16090 4.25133
H 1.63608 5.08429 0.52821
H 0.72991 -0.62000 5.19381
H 1.21553 4.15733 3.03979
H 1.45041 -5.37344 -0.46001
H 2.41005 2.85946 -4.16910
H 1.97187 -4.45280 -2.95616
H 2.56461 0.31570 -5.09319
C 1.23852 -3.24535 1.43357
C 1.92324 2.94835 -1.36155
C 2.14516 -1.59855 -3.01774
C 1.14283 1.29577 3.11951
H 1.11492 -4.22833 1.87790
H 2.00179 3.93036 -1.81845
H 2.30781 -2.05294 -3.99028
H 0.98851 1.74754 4.09469
O -0.14585 -0.12527 -0.31502
S 3.87056 -0.10781 0.72577
C 4.81942 0.99956 -0.36220
H 4.41758 2.01651 -0.29352
H 5.85840 1.01254 -0.02017
H 4.76741 0.67347 -1.40384
H -0.80888 0.72239 0.16037
C -1.73463 1.56208 0.73360
O -2.88893 1.62350 -0.02770
H -1.26939 2.54610 0.74182
H -1.88302 1.11230 1.72037
C -3.75882 0.54737 -0.02460
C -3.38685 -0.77176 0.24433
C -5.07726 0.89490 -0.34546
C -4.37312 -1.75351 0.20203
C -6.06091 -0.11089 -0.38560
O -5.42541 2.18493 -0.60858
C -5.70028 -1.43301 -0.10529
H -4.10708 -2.78800 0.39758
H -4.60877 2.70897 -0.53132
H -6.44665 -2.21828 -0.13342
O -7.31855 0.31663 -0.69779
C -8.35125 -0.65010 -0.74809
H -9.25963 -0.10381 -1.00964
H -8.15550 -1.41421 -1.51294
H -8.49289 -1.14431 0.22305
H -2.34728 -1.01874 0.42874

TS1-b - quartet

Fe -1.17312 0.17009 0.20812
N 0.29609 -0.32612 1.50594

| | | | |
|---|---|---|---|
| N | -1.80559 | -1.77512 | 0.25511 |
| N | -0.59830 | 2.08969 | 0.25052 |
| N | -2.75435 | 0.66619 | -0.91659 |
| C | 1.26934 | 0.51689 | 1.97863 |
| C | -2.86771 | -2.29859 | -0.43684 |
| C | 0.58759 | -1.56884 | 2.01586 |
| C | -1.24008 | -2.82721 | 0.93906 |
| C | 0.49892 | 2.59829 | 0.89183 |
| C | -3.66985 | -0.19595 | -1.46838 |
| C | -1.19205 | 3.14898 | -0.38212 |
| C | -3.06372 | 1.91206 | -1.40318 |
| C | 2.19007 | -0.20782 | 2.81897 |
| C | -2.97653 | -3.71687 | -0.18646 |
| C | 1.76561 | -1.50124 | 2.84427 |
| C | -1.96782 | -4.04344 | 0.66796 |
| C | 0.60861 | 4.01872 | 0.64766 |
| C | -4.59349 | 0.52966 | -2.30589 |
| C | -0.44399 | 4.36205 | -0.14067 |
| C | -4.22030 | 1.83866 | -2.26139 |
| H | 3.04718 | 0.23067 | 3.31344 |
| H | -3.73313 | -4.36262 | -0.61408 |
| H | 2.20282 | -2.34487 | 3.36287 |
| H | -1.72617 | -5.01242 | 1.08610 |
| H | 1.39598 | 4.65072 | 1.03757 |
| H | -5.41268 | 0.08024 | -2.85283 |
| H | -0.70505 | 5.33602 | -0.53442 |
| H | -4.66745 | 2.68532 | -2.76655 |
| C | 1.37022 | 1.87331 | 1.69321 |
| C | -3.72923 | -1.56779 | -1.24858 |
| C | -2.35038 | 3.07567 | -1.14396 |
| C | -0.12378 | -2.73545 | 1.75831 |
| H | 2.20763 | 2.40930 | 2.12738 |
| H | -4.52324 | -2.11444 | -1.74871 |
| H | -2.71287 | 3.99444 | -1.59484 |
| H | 0.22479 | -3.64687 | 2.23443 |
| O | -0.16845 | -0.10342 | -1.20141 |
| S | -2.47320 | 0.31293 | 2.26298 |
| C | -4.24492 | 0.20746 | 1.86699 |
| H | -4.45781 | -0.75268 | 1.38320 |
| H | -4.81273 | 0.26003 | 2.80044 |
| H | -4.54855 | 1.01127 | 1.19167 |
| H | 0.21975 | -1.23741 | -1.41506 |
| C | 0.94646 | -2.24374 | -1.86788 |
| O | 2.00714 | -1.66681 | -2.54562 |
| H | 0.29672 | -2.76190 | -2.57532 |
| H | 1.23663 | -2.83015 | -0.99060 |
| C | 3.13382 | -1.33313 | -1.79706 |
| C | 4.14003 | -2.29036 | -1.63536 |
| C | 3.26011 | -0.02873 | -1.29631 |
| C | 5.31593 | -1.94205 | -0.98388 |
| H | 3.99078 | -3.28025 | -2.05549 |
| C | 4.47336 | 0.30713 | -0.64050 |
| O | 2.32863 | 0.93618 | -1.43301 |
| C | 5.48542 | -0.64217 | -0.49124 |
| H | 6.11127 | -2.67137 | -0.86216 |
| H | 1.41096 | 0.55954 | -1.49396 |
| H | 6.40882 | -0.38038 | 0.01245 |
| O | 4.54361 | 1.59431 | -0.18395 |

| | | | |
|---|---|---|---|
| C | 5.76189 | 2.02346 | 0.39137 |
| H | 5.62261 | 3.07810 | 0.63899 |
| H | 6.00167 | 1.46638 | 1.30866 |
| H | 6.60061 | 1.92563 | -0.31160 |

Int1-b - doublet

| | | | |
|---|---|---|---|
| Fe | -1.25528 | -0.03555 | 0.20428 |
| N | -0.26893 | 1.32581 | 1.31679 |
| N | -0.00003 | -1.48251 | 0.82637 |
| N | -2.57022 | 1.40329 | -0.33281 |
| N | -2.28895 | -1.41940 | -0.84564 |
| C | -0.59646 | 2.64954 | 1.51499 |
| C | -0.03295 | -2.81862 | 0.50293 |
| C | 0.88562 | 1.11412 | 2.02291 |
| C | 1.11812 | -1.31127 | 1.60762 |
| C | -2.59937 | 2.70897 | 0.07066 |
| C | -2.00468 | -2.74717 | -0.98436 |
| C | -3.61979 | 1.24909 | -1.19884 |
| C | -3.36411 | -1.17340 | -1.65884 |
| C | 0.38025 | 3.28217 | 2.36772 |
| C | 1.07208 | -3.51068 | 1.12397 |
| C | 1.30550 | 2.33252 | 2.67428 |
| C | 1.78961 | -2.57558 | 1.79984 |
| C | -3.69305 | 3.40674 | -0.55943 |
| C | -2.93183 | -3.36984 | -1.89962 |
| C | -4.32533 | 2.49827 | -1.35507 |
| C | -3.77715 | -2.38897 | -2.32163 |
| H | 0.34964 | 4.31929 | 2.67668 |
| H | 1.26789 | -4.57070 | 1.02489 |
| H | 2.19096 | 2.42743 | 3.28973 |
| H | 2.69566 | -2.70749 | 2.37684 |
| H | -3.93115 | 4.45170 | -0.40644 |
| H | -2.91950 | -4.41629 | -2.17678 |
| H | -5.19152 | 2.64391 | -1.98811 |
| H | -4.60384 | -2.46374 | -3.01676 |
| C | -1.68778 | 3.29248 | 0.95113 |
| C | -0.96583 | -3.41315 | -0.33417 |
| C | -3.98567 | 0.05911 | -1.82187 |
| C | 1.54659 | -0.10591 | 2.14165 |
| H | -1.82940 | 4.34162 | 1.19288 |
| H | -0.85903 | -4.47695 | -0.52410 |
| H | -4.84099 | 0.09596 | -2.49031 |
| H | 2.45793 | -0.11955 | 2.73123 |
| O | -0.23919 | 0.29107 | -1.29153 |
| S | -2.44699 | -0.49595 | 2.25535 |
| C | -3.22849 | -2.13608 | 2.14423 |
| H | -2.46624 | -2.90734 | 1.98476 |
| H | -3.74395 | -2.34242 | 3.08663 |
| H | -3.93878 | -2.17741 | 1.31388 |
| H | -0.09023 | 1.24942 | -1.33423 |
| C | 2.03004 | 3.24659 | -2.36730 |
| O | 2.01133 | 2.02275 | -1.79896 |
| H | 1.05998 | 3.55903 | -2.73071 |
| H | 2.95489 | 3.60129 | -2.80965 |

C 3.17401 1.45955 -1.30174
C 4.28361 2.23139 -0.93802
C 3.16277 0.05436 -1.18033
C 5.43129 1.59495 -0.48391
H 4.22903 3.31304 -0.99063
C 4.35865 -0.56562 -0.72083
O 2.13730 -0.73141 -1.51462
C 5.47643 0.19835 -0.38560
H 6.29770 2.18212 -0.19461
H 1.22790 -0.27425 -1.48005
H 6.38201 -0.28215 -0.03407
O 4.29900 -1.92614 -0.64910
C 5.46840 -2.62152 -0.26397
H 5.21151 -3.68209 -0.30013
H 6.30256 -2.42698 -0.95224
H 5.78255 -2.36071 0.75689

Int1-a - quartet

Fe -1.65803 0.09007 0.07373
N -2.37919 1.34648 -1.33345
N -0.64112 1.60756 0.92167
N -2.84004 -1.36850 -0.65833
N -1.00808 -1.13848 1.54379
C -3.32604 1.07575 -2.29505
C 0.16361 1.55409 2.03871
C -2.00456 2.64959 -1.53279
C -0.50027 2.88149 0.41348
C -3.72007 -1.29013 -1.70362
C -0.13692 -0.84505 2.55207
C -2.87134 -2.66948 -0.23254
C -1.27540 -2.47923 1.65214
C -3.54270 2.23627 -3.12499
C 0.79214 2.83416 2.25988
C -2.71472 3.21043 -2.65819
C 0.38774 3.65229 1.24979
C -4.33170 -2.57382 -1.94335
C 0.15607 -2.02733 3.33008
C -3.79771 -3.43452 -1.03148
C -0.55046 -3.04546 2.76767
H -4.23886 2.28200 -3.95274
H 1.46752 3.05524 3.07651
H -2.59405 4.22332 -3.02070
H 0.65857 4.68509 1.07090
H -5.06337 -2.77621 -2.71507
H 0.81987 -2.05720 4.18466
H -4.00402 -4.48892 -0.89840
H -0.58910 -4.08532 3.06639
C -3.96672 -0.14438 -2.45792
C 0.39348 0.42019 2.80441
C -2.13958 -3.19180 0.83183
C -1.11086 3.35934 -0.73517
H -4.69599 -0.22257 -3.25864
H 1.07358 0.51729 3.64527
H -2.26696 -4.24902 1.04541

| | | | |
|---|---|---|---|
| H | -0.90250 | 4.38697 | -1.01710 |
| O | -0.25411 | -0.41889 | -0.93453 |
| S | -3.44859 | 1.04926 | 1.48339 |
| C | -3.17633 | 0.57002 | 3.21465 |
| H | -2.21918 | 0.97947 | 3.56033 |
| H | -3.97738 | 0.98975 | 3.82964 |
| H | -3.14329 | -0.51716 | 3.32315 |
| H | -0.53211 | -0.35459 | -1.86499 |
| C | 2.15413 | -2.49863 | -1.87286 |
| O | 3.48108 | -2.24175 | -1.67141 |
| H | 1.99234 | -3.51202 | -2.21800 |
| H | 1.40760 | -1.94703 | -1.31211 |
| C | 3.92313 | -1.04588 | -1.13711 |
| C | 3.11428 | 0.03422 | -0.77668 |
| C | 5.31688 | -1.00705 | -0.98615 |
| C | 3.73224 | 1.16709 | -0.24852 |
| H | 2.03627 | -0.00117 | -0.89682 |
| C | 5.92089 | 0.14492 | -0.45364 |
| O | 6.09420 | -2.06836 | -1.34527 |
| C | 5.11972 | 1.23147 | -0.08481 |
| H | 3.12120 | 2.01723 | 0.04110 |
| H | 5.48382 | -2.74336 | -1.69041 |
| H | 5.57069 | 2.12727 | 0.32638 |
| O | 7.28239 | 0.09204 | -0.34178 |
| C | 7.94428 | 1.22775 | 0.17877 |
| H | 7.62558 | 1.45022 | 1.20697 |
| H | 9.00821 | 0.98145 | 0.17842 |
| H | 7.77912 | 2.11753 | -0.44492 |

Int1-b - quartet

| | | | |
|---|---|---|---|
| Fe | 1.33967 | 0.07866 | 0.10195 |
| N | 1.43782 | -1.85365 | 0.67802 |
| N | 0.23641 | 0.51719 | 1.72333 |
| N | 2.60650 | -0.33482 | -1.41211 |
| N | 1.30631 | 2.03031 | -0.42272 |
| C | 2.17929 | -2.86342 | 0.10635 |
| C | -0.26256 | 1.74837 | 2.09036 |
| C | 0.75378 | -2.43083 | 1.71462 |
| C | -0.28369 | -0.38481 | 2.62870 |
| C | 3.19338 | -1.53341 | -1.71176 |
| C | 0.64181 | 3.04901 | 0.19412 |
| C | 3.00034 | 0.53595 | -2.39133 |
| C | 1.86077 | 2.57030 | -1.55512 |
| C | 1.94403 | -4.10816 | 0.79656 |
| C | -1.07566 | 1.62291 | 3.27510 |
| C | 1.04995 | -3.84173 | 1.78899 |
| C | -1.09554 | 0.30332 | 3.60228 |
| C | 3.98953 | -1.41883 | -2.90805 |
| C | 0.78064 | 4.27498 | -0.55759 |
| C | 3.86270 | -0.13069 | -3.33603 |
| C | 1.53675 | 3.97585 | -1.64904 |
| H | 2.40946 | -5.05224 | 0.54330 |
| H | -1.58135 | 2.44596 | 3.76335 |
| H | 0.63313 | -4.52133 | 2.52134 |

| | | | |
|---|---|---|---|
| H | -1.61587 | -0.18007 | 4.41914 |
| H | 4.55538 | -2.22627 | -3.35497 |
| H | 0.34495 | 5.22538 | -0.27711 |
| H | 4.30645 | 0.33782 | -4.20529 |
| H | 1.85371 | 4.63089 | -2.45062 |
| C | 3.01532 | -2.71302 | -0.99051 |
| C | -0.06919 | 2.92830 | 1.38851 |
| C | 2.64750 | 1.88203 | -2.46806 |
| C | -0.06740 | -1.75199 | 2.61171 |
| H | 3.54590 | -3.59433 | -1.33837 |
| H | -0.54903 | 3.82171 | 1.77613 |
| H | 3.03343 | 2.44481 | -3.31300 |
| H | -0.55335 | -2.34109 | 3.38331 |
| O | -0.11271 | -0.18799 | -0.95297 |
| S | 3.15866 | 0.39517 | 1.72000 |
| C | 3.36891 | 2.17017 | 2.04977 |
| H | 2.44673 | 2.57706 | 2.48238 |
| H | 4.17982 | 2.30070 | 2.77204 |
| H | 3.58872 | 2.71964 | 1.13068 |
| H | -0.13064 | -1.12318 | -1.22608 |
| C | -1.71053 | -3.02550 | -1.24058 |
| O | -2.26602 | -2.15002 | -0.35454 |
| H | -1.00453 | -3.70853 | -0.78314 |
| H | -2.28505 | -3.29632 | -2.12069 |
| C | -3.49100 | -1.56676 | -0.65375 |
| C | -4.59868 | -2.34698 | -1.00166 |
| C | -3.57060 | -0.17218 | -0.51906 |
| C | -5.82583 | -1.72890 | -1.20607 |
| H | -4.48892 | -3.42457 | -1.07409 |
| C | -4.83938 | 0.43235 | -0.71312 |
| O | -2.53074 | 0.61452 | -0.19412 |
| C | -5.94926 | -0.34198 | -1.05680 |
| H | -6.69738 | -2.32104 | -1.46910 |
| H | -1.64764 | 0.20087 | -0.42699 |
| H | -6.91403 | 0.12651 | -1.21349 |
| O | -4.85789 | 1.78870 | -0.55029 |
| C | -6.08260 | 2.46205 | -0.75981 |
| H | -5.87558 | 3.52119 | -0.59172 |
| H | -6.45359 | 2.32547 | -1.78551 |
| H | -6.85805 | 2.12994 | -0.05485 |

TS2-b - quartet

| | | | |
|---|---|---|---|
| Fe | -1.21047 | 0.20705 | 0.21253 |
| N | -1.64898 | 2.06708 | -0.43347 |
| N | 0.38702 | 0.94908 | 1.18371 |
| N | -2.83143 | -0.55077 | -0.73520 |
| N | -0.77568 | -1.65770 | 0.86608 |
| C | -2.68823 | 2.40548 | -1.25950 |
| C | 1.30306 | 0.23610 | 1.90705 |
| C | -0.98608 | 3.22432 | -0.16316 |
| C | 0.78296 | 2.26739 | 1.26524 |
| C | -3.70339 | 0.15748 | -1.51504 |
| C | 0.30672 | -2.01265 | 1.63457 |
| C | -3.25915 | -1.85335 | -0.77287 |

| | | | |
|---|---|---|---|
| C | -1.48135 | -2.81706 | 0.64742 |
| C | -2.67175 | 3.82777 | -1.52360 |
| C | 2.30084 | 1.12065 | 2.46626 |
| C | -1.61352 | 4.33828 | -0.83784 |
| C | 1.97463 | 2.37947 | 2.07192 |
| C | -4.71534 | -0.71746 | -2.05668 |
| C | 0.27888 | -3.42956 | 1.91033 |
| C | -4.43993 | -1.96943 | -1.59291 |
| C | -0.83347 | -3.92788 | 1.30277 |
| H | -3.38376 | 4.34747 | -2.15212 |
| H | 3.13346 | 0.79844 | 3.07854 |
| H | -1.27286 | 5.36441 | -0.78547 |
| H | 2.48378 | 3.30940 | 2.28965 |
| H | -5.52231 | -0.40169 | -2.70571 |
| H | 1.01687 | -3.95638 | 2.50208 |
| H | -4.97333 | -2.89212 | -1.78347 |
| H | -1.19374 | -4.94885 | 1.28773 |
| C | -3.63993 | 1.52959 | -1.76012 |
| C | 1.28393 | -1.14187 | 2.09940 |
| C | -2.63960 | -2.91348 | -0.11725 |
| C | 0.14407 | 3.32859 | 0.65095 |
| H | -4.40958 | 1.94984 | -2.40092 |
| H | 2.08582 | -1.56930 | 2.69364 |
| H | -3.08920 | -3.89668 | -0.22056 |
| H | 0.56814 | 4.31799 | 0.79152 |
| O | -0.19881 | -0.03633 | -1.36554 |
| S | -2.36397 | 0.60328 | 2.21444 |
| C | -3.71748 | -0.60706 | 2.38667 |
| H | -3.32953 | -1.63099 | 2.38548 |
| H | -4.21636 | -0.42238 | 3.34269 |
| H | -4.44168 | -0.50735 | 1.57323 |
| H | -0.26092 | -0.95893 | -1.65593 |
| C | 1.56957 | -3.29407 | -1.82634 |
| O | 1.87779 | -1.97096 | -1.97810 |
| H | 0.66102 | -3.56426 | -2.35071 |
| H | 1.83240 | -3.76429 | -0.88233 |
| C | 3.06450 | -1.49645 | -1.46231 |
| C | 4.17108 | -2.31509 | -1.25300 |
| C | 3.10342 | -0.09607 | -1.20140 |
| C | 5.35941 | -1.75862 | -0.77591 |
| H | 4.10946 | -3.36905 | -1.50084 |
| C | 4.35026 | 0.43874 | -0.73039 |
| O | 2.10087 | 0.72184 | -1.39071 |
| C | 5.45386 | -0.38773 | -0.51729 |
| H | 6.22554 | -2.39463 | -0.61937 |
| H | 1.05150 | 0.30070 | -1.41160 |
| H | 6.38885 | 0.02489 | -0.15687 |
| O | 4.33741 | 1.78049 | -0.52550 |
| C | 5.52549 | 2.39752 | -0.06506 |
| H | 5.29595 | 3.46123 | 0.02051 |
| H | 5.82783 | 2.01036 | 0.91767 |
| H | 6.35304 | 2.26106 | -0.77416 |

TS2-a - quartet

| | | | |
|---|---|---|---|
| Fe | -1.59804 | 0.05437 | 0.23260 |
| N | -0.53255 | -0.46411 | 1.86960 |
| N | -1.81540 | -1.89901 | -0.25057 |
| N | -1.30811 | 2.00568 | 0.66549 |
| N | -2.74368 | 0.57687 | -1.33653 |
| C | 0.09026 | 0.38194 | 2.74854 |
| C | -2.45637 | -2.40348 | -1.34939 |
| C | -0.23954 | -1.74091 | 2.28127 |
| C | -1.33275 | -2.97391 | 0.44708 |
| C | -0.58725 | 2.51546 | 1.71946 |
| C | -3.25464 | -0.26903 | -2.29161 |
| C | -1.86741 | 3.08536 | 0.02365 |
| C | -3.08679 | 1.84870 | -1.72223 |
| C | 0.78005 | -0.38095 | 3.76073 |
| C | -2.36956 | -3.84601 | -1.35275 |
| C | 0.57238 | -1.69648 | 3.47305 |
| C | -1.67644 | -4.19963 | -0.23614 |
| C | -0.66623 | 3.95646 | 1.71818 |
| C | -3.96358 | 0.49091 | -3.29065 |
| C | -1.46250 | 4.30885 | 0.67231 |
| C | -3.86526 | 1.80249 | -2.93488 |
| H | 1.34553 | 0.05016 | 4.57710 |
| H | -2.79494 | -4.48695 | -2.11465 |
| H | 0.93436 | -2.56824 | 4.00319 |
| H | -1.41204 | -5.19127 | 0.10856 |
| H | -0.17940 | 4.59824 | 2.44147 |
| H | -4.46276 | 0.06133 | -4.15006 |
| H | -1.76259 | 5.29998 | 0.35665 |
| H | -4.26385 | 2.67124 | -3.44332 |
| C | 0.07598 | 1.77014 | 2.68297 |
| C | -3.12341 | -1.65104 | -2.30878 |
| C | -2.70201 | 3.02095 | -1.08227 |
| C | -0.61408 | -2.91027 | 1.63363 |
| H | 0.60857 | 2.31323 | 3.45709 |
| H | -3.58922 | -2.18631 | -3.13038 |
| H | -3.06117 | 3.95826 | -1.49526 |
| H | -0.29996 | -3.84903 | 2.07894 |
| O | -0.08731 | 0.03760 | -0.81666 |
| S | -3.42957 | -0.08548 | 1.67471 |
| C | -4.94480 | -0.40852 | 0.70798 |
| H | -4.84831 | -1.34917 | 0.15796 |
| H | -5.78205 | -0.48783 | 1.40742 |
| H | -5.13930 | 0.39945 | -0.00178 |
| H | 0.16693 | 0.97135 | -0.92848 |
| C | 1.84671 | -0.48696 | -2.48034 |
| O | 3.05090 | 0.11678 | -2.30552 |
| H | 1.33175 | -0.16396 | -3.37366 |
| H | 1.67648 | -1.44886 | -2.01746 |
| C | 3.77279 | -0.14568 | -1.14585 |
| C | 3.18944 | -0.49839 | 0.06820 |
| C | 5.16088 | -0.00481 | -1.29734 |
| C | 4.02714 | -0.72625 | 1.16275 |
| H | 2.10869 | -0.56288 | 0.14775 |
| C | 5.98674 | -0.21884 | -0.18237 |
| O | 5.70358 | 0.31785 | -2.50861 |
| C | 5.40902 | -0.58314 | 1.03835 |
| H | 3.59033 | -0.99822 | 2.11878 |
| H | 4.96098 | 0.40868 | -3.13147 |

| | | | |
|---|---|---|---|
| H | 6.07383 | -0.75196 | 1.87968 |
| O | 7.35515 | -0.15333 | -0.26904 |
| C | 7.90712 | 1.11211 | -0.63919 |
| H | 8.99179 | 0.99245 | -0.58492 |
| H | 7.61859 | 1.39801 | -1.65480 |
| H | 7.59402 | 1.89479 | 0.06523 |

TS1-c - doublet

| | | | |
|---|---|---|---|
| Fe | -1.17081 | 0.18752 | 0.12855 |
| N | -1.80704 | 2.00881 | -0.47404 |
| N | 0.34324 | 1.05647 | 1.13445 |
| N | -2.80335 | -0.65856 | -0.69168 |
| N | -0.64015 | -1.60953 | 0.90000 |
| C | -2.90263 | 2.27560 | -1.24993 |
| C | 1.30301 | 0.42742 | 1.88317 |
| C | -1.19636 | 3.20851 | -0.24861 |
| C | 0.66038 | 2.39466 | 1.15041 |
| C | -3.75622 | -0.02834 | -1.44799 |
| C | 0.46227 | -1.87831 | 1.67048 |
| C | -3.10879 | -1.99539 | -0.72774 |
| C | -1.24924 | -2.81553 | 0.66846 |
| C | -2.98438 | 3.69194 | -1.52608 |
| C | 2.24644 | 1.39223 | 2.39330 |
| C | -1.92393 | 4.27190 | -0.90340 |
| C | 1.84727 | 2.61079 | 1.93948 |
| C | -4.69965 | -0.99036 | -1.96180 |
| C | 0.54242 | -3.29322 | 1.94647 |
| C | -4.29665 | -2.21247 | -1.51551 |
| C | -0.52104 | -3.87509 | 1.32662 |
| H | -3.76000 | 4.15968 | -2.11903 |
| H | 3.09618 | 1.14766 | 3.01783 |
| H | -1.64372 | 5.31716 | -0.87574 |
| H | 2.30005 | 3.57861 | 2.11246 |
| H | -5.55069 | -0.74564 | -2.58458 |
| H | 1.31341 | -3.75987 | 2.54658 |
| H | -4.74902 | -3.17970 | -1.69443 |
| H | -0.80483 | -4.91997 | 1.30970 |
| C | -3.81530 | 1.33661 | -1.70443 |
| C | 1.37557 | -0.93838 | 2.12484 |
| C | -2.39583 | -3.00397 | -0.09251 |
| C | -0.04867 | 3.39894 | 0.51325 |
| H | -4.63774 | 1.69322 | -2.31678 |
| H | 2.20264 | -1.29501 | 2.73046 |
| H | -2.76579 | -4.01940 | -0.19761 |
| H | 0.32261 | 4.41388 | 0.61336 |
| O | -0.25270 | -0.03113 | -1.26111 |
| S | -2.35272 | 0.55857 | 2.25099 |
| C | -3.67013 | -0.69448 | 2.41914 |
| H | -3.25120 | -1.70608 | 2.43474 |
| H | -4.18047 | -0.51716 | 3.37130 |
| H | -4.39607 | -0.62712 | 1.60396 |
| O | 1.85003 | -1.97199 | -1.73051 |
| C | 3.03582 | -1.49003 | -1.30252 |
| C | 4.16445 | -2.25872 | -1.01952 |

| | | | |
|---|---|---|---|
| C | 3.06252 | -0.05810 | -1.16477 |
| C | 5.34591 | -1.62734 | -0.62238 |
| H | 4.13577 | -3.33733 | -1.11914 |
| C | 4.30564 | 0.54836 | -0.76603 |
| O | 2.03945 | 0.70045 | -1.41264 |
| C | 5.42624 | -0.23447 | -0.49941 |
| H | 6.22476 | -2.22933 | -0.41043 |
| H | 0.95845 | 0.25268 | -1.36394 |
| H | 6.36106 | 0.22189 | -0.19648 |
| O | 4.26260 | 1.89928 | -0.68730 |
| C | 5.44092 | 2.58492 | -0.30004 |
| H | 5.18450 | 3.64557 | -0.30700 |
| H | 5.76248 | 2.29352 | 0.70883 |
| H | 6.26213 | 2.40418 | -1.00620 |
| C | 1.68298 | -3.37580 | -1.86065 |
| H | 2.37689 | -3.78955 | -2.60407 |
| H | 1.82599 | -3.88157 | -0.89819 |
| H | 0.65530 | -3.51744 | -2.19591 |

TS1-c - quartet

| | | | |
|---|---|---|---|
| Fe | -1.18438 | 0.19072 | 0.13908 |
| N | -2.10430 | 1.81442 | -0.63860 |
| N | 0.19898 | 1.38976 | 0.99144 |
| N | -2.66690 | -0.98526 | -0.54434 |
| N | -0.34946 | -1.40959 | 1.05884 |
| C | -3.24488 | 1.82325 | -1.39311 |
| C | 1.25271 | 1.00353 | 1.77909 |
| C | -1.67373 | 3.10925 | -0.57782 |
| C | 0.30482 | 2.75092 | 0.84954 |
| C | -3.73118 | -0.59320 | -1.31480 |
| C | 0.79162 | -1.41732 | 1.81842 |
| C | -2.75228 | -2.34970 | -0.43904 |
| C | -0.75469 | -2.71627 | 0.96025 |
| C | -3.54488 | 3.16909 | -1.82673 |
| C | 2.03675 | 2.15463 | 2.15712 |
| C | -2.56723 | 3.96808 | -1.32146 |
| C | 1.44892 | 3.23734 | 1.58153 |
| C | -4.52022 | -1.74047 | -1.69192 |
| C | 1.10985 | -2.76634 | 2.22254 |
| C | -3.91076 | -2.83152 | -1.15076 |
| C | 0.14934 | -3.57273 | 1.69206 |
| H | -4.39566 | 3.44431 | -2.43699 |
| H | 2.91903 | 2.11704 | 2.78330 |
| H | -2.44591 | 5.03857 | -1.42769 |
| H | 1.74695 | 4.27671 | 1.63386 |
| H | -5.41700 | -1.69860 | -2.29701 |
| H | 1.95847 | -3.04005 | 2.83658 |
| H | -4.20509 | -3.87141 | -1.21677 |
| H | 0.04244 | -4.64649 | 1.78354 |
| C | -4.01366 | 0.71035 | -1.70324 |
| C | 1.54413 | -0.29997 | 2.15626 |
| C | -1.86836 | -3.16228 | 0.26091 |
| C | -0.55636 | 3.55523 | 0.11858 |
| H | -4.89817 | 0.86776 | -2.31277 |

| | | | |
|---|---|---|---|
| H | 2.42422 | -0.45750 | 2.77165 |
| H | -2.06960 | -4.22936 | 0.26758 |
| H | -0.34231 | 4.61905 | 0.09085 |
| O | -0.26350 | -0.00947 | -1.25154 |
| S | -2.35877 | 0.61040 | 2.22172 |
| C | -3.36103 | -0.85578 | 2.64155 |
| H | -2.72726 | -1.74093 | 2.75597 |
| H | -3.86655 | -0.66283 | 3.59265 |
| H | -4.10709 | -1.05816 | 1.86774 |
| O | 1.82481 | -1.95416 | -1.78225 |
| C | 3.01196 | -1.48310 | -1.34641 |
| C | 4.13407 | -2.26059 | -1.06100 |
| C | 3.04427 | -0.05441 | -1.19386 |
| C | 5.31440 | -1.63781 | -0.64789 |
| H | 4.10149 | -3.33797 | -1.17144 |
| C | 4.28223 | 0.54307 | -0.77124 |
| O | 2.02487 | 0.70930 | -1.45113 |
| C | 5.39872 | -0.24703 | -0.50669 |
| H | 6.18968 | -2.24535 | -0.43680 |
| H | 0.95333 | 0.27026 | -1.37936 |
| H | 6.33260 | 0.20223 | -0.19052 |
| O | 4.24164 | 1.89246 | -0.67092 |
| C | 5.41928 | 2.57061 | -0.26876 |
| H | 5.16261 | 3.63105 | -0.25665 |
| H | 5.73939 | 2.26124 | 0.73530 |
| H | 6.24184 | 2.40214 | -0.97639 |
| C | 1.64052 | -3.35772 | -1.89366 |
| H | 2.32084 | -3.78750 | -2.64053 |
| H | 1.79173 | -3.85225 | -0.92661 |
| H | 0.60736 | -3.49197 | -2.21475 |

Int1-c - quartet

| | | | |
|---|---|---|---|
| Fe | -1.41830 | 0.20217 | 0.13328 |
| N | -2.73106 | 0.52998 | -1.35508 |
| N | -0.99569 | 2.17520 | 0.20248 |
| N | -1.92395 | -1.74438 | 0.15839 |
| N | -0.08634 | -0.12852 | 1.62980 |
| C | -3.55766 | -0.39098 | -1.94094 |
| C | -0.12315 | 2.80036 | 1.05766 |
| C | -2.96490 | 1.72009 | -1.99208 |
| C | -1.47054 | 3.13998 | -0.64391 |
| C | -2.85459 | -2.35862 | -0.63976 |
| C | 0.66093 | 0.81463 | 2.28691 |
| C | -1.32536 | -2.73914 | 0.89370 |
| C | 0.24983 | -1.33937 | 2.17444 |
| C | -4.33549 | 0.23477 | -2.98651 |
| C | -0.06478 | 4.20975 | 0.75369 |
| C | -3.96264 | 1.54170 | -3.02292 |
| C | -0.89461 | 4.41944 | -0.30450 |
| C | -2.86734 | -3.77736 | -0.38009 |
| C | 1.49795 | 0.17819 | 3.27526 |
| C | -1.91433 | -4.01431 | 0.56492 |
| C | 1.24806 | -1.15805 | 3.20215 |
| H | -5.06349 | -0.27823 | -3.60227 |
| H | 0.54898 | 4.92708 | 1.28350 |

| | | | |
|---|---|---|---|
| H | -4.32266 | 2.32964 | -3.67241 |
| H | -1.10847 | 5.34601 | -0.82212 |
| H | -3.51885 | -4.48706 | -0.87436 |
| H | 2.18424 | 0.70227 | 3.92829 |
| H | -1.62722 | -4.95830 | 1.01142 |
| H | 1.68218 | -1.95937 | 3.78700 |
| C | -3.63808 | -1.73319 | -1.60186 |
| C | 0.63614 | 2.18017 | 2.03921 |
| C | -0.31255 | -2.56229 | 1.82710 |
| C | -2.37791 | 2.93606 | -1.67630 |
| H | -4.33878 | -2.35047 | -2.15546 |
| H | 1.29030 | 2.80829 | 2.63534 |
| H | 0.05219 | -3.44567 | 2.34260 |
| H | -2.66600 | 3.80031 | -2.26653 |
| O | -0.15427 | -0.07823 | -1.06664 |
| S | -2.99839 | 0.75186 | 1.73400 |
| C | -3.32518 | -0.69931 | 2.79222 |
| H | -2.40776 | -1.01666 | 3.29681 |
| H | -4.06160 | -0.40527 | 3.54591 |
| H | -3.71504 | -1.53573 | 2.20676 |
| O | 2.19908 | -1.91488 | -1.03817 |
| C | 3.46913 | -1.49484 | -1.06092 |
| C | 4.57601 | -2.26371 | -1.39772 |
| C | 3.60819 | -0.08127 | -0.68673 |
| C | 5.85128 | -1.68217 | -1.36498 |
| H | 4.46773 | -3.30407 | -1.68171 |
| C | 4.97394 | 0.45498 | -0.66551 |
| O | 2.61799 | 0.62982 | -0.40968 |
| C | 6.05987 | -0.34106 | -1.00279 |
| H | 6.71157 | -2.29124 | -1.62780 |
| H | 0.75237 | 0.07836 | -0.71601 |
| H | 7.06866 | 0.05483 | -0.99360 |
| O | 5.03513 | 1.74927 | -0.30428 |
| C | 6.30708 | 2.38250 | -0.26978 |
| H | 6.11765 | 3.41214 | 0.03636 |
| H | 6.97260 | 1.89935 | 0.45687 |
| H | 6.78153 | 2.37481 | -1.25915 |
| C | 1.89076 | -3.24143 | -1.44957 |
| H | 2.20136 | -3.40970 | -2.48800 |
| H | 2.37621 | -3.97803 | -0.79702 |
| H | 0.80800 | -3.32666 | -1.36519 |

TS2-c - doublet

| | | | |
|---|---|---|---|
| Fe | -1.44575 | 0.28713 | 0.07710 |
| N | -0.65340 | 1.03212 | 1.79665 |
| N | -1.71190 | -1.51051 | 0.95223 |
| N | -1.05819 | 2.06046 | -0.81229 |
| N | -2.13931 | -0.47208 | -1.64512 |
| C | -0.17842 | 2.29729 | 2.02529 |
| C | -2.20283 | -2.65679 | 0.37721 |
| C | -0.55963 | 0.36990 | 2.99406 |
| C | -1.46135 | -1.81928 | 2.26756 |
| C | -0.51378 | 3.18771 | -0.25128 |
| C | -2.56178 | -1.75433 | -1.89037 |

| | | | |
|---|---|---|---|
| C | -1.35279 | 2.38553 | -2.11049 |
| C | -2.27746 | 0.20567 | -2.82761 |
| C | 0.23070 | 2.43853 | 3.40347 |
| C | -2.26031 | -3.71861 | 1.35291 |
| C | -0.00657 | 1.24189 | 4.00545 |
| C | -1.80244 | -3.19788 | 2.52639 |
| C | -0.45008 | 4.25110 | -1.22795 |
| C | -2.98650 | -1.88762 | -3.26521 |
| C | -0.97344 | 3.75441 | -2.38087 |
| C | -2.81435 | -0.66931 | -3.84587 |
| H | 0.64386 | 3.34141 | 3.83502 |
| H | -2.61118 | -4.72330 | 1.15272 |
| H | 0.17070 | 0.95773 | 5.03510 |
| H | -1.69924 | -3.68872 | 3.48610 |
| H | -0.05729 | 5.24164 | -1.03589 |
| H | -3.36314 | -2.80047 | -3.70939 |
| H | -1.09911 | 4.25167 | -3.33446 |
| H | -3.01782 | -0.37397 | -4.86755 |
| C | -0.10247 | 3.30564 | 1.07050 |
| C | -2.59124 | -2.77902 | -0.95290 |
| C | -1.92822 | 1.53378 | -3.04474 |
| C | -0.92741 | -0.95218 | 3.21534 |
| H | 0.30192 | 4.26292 | 1.38515 |
| H | -2.95722 | -3.74769 | -1.28032 |
| H | -2.10219 | 1.93465 | -4.03902 |
| H | -0.78753 | -1.34338 | 4.21885 |
| O | 0.37315 | -0.24203 | -0.51735 |
| S | -3.45782 | 1.03284 | 0.78684 |
| C | -4.74145 | -0.19230 | 0.34940 |
| H | -4.54680 | -1.15957 | 0.82141 |
| H | -5.69818 | 0.19139 | 0.71756 |
| H | -4.80441 | -0.33130 | -0.73326 |
| H | 0.84013 | -1.33254 | -0.04614 |
| C | 1.50911 | -2.28020 | 0.53151 |
| O | 2.57972 | -2.65602 | -0.27747 |
| H | 0.85935 | -3.15021 | 0.61872 |
| H | 1.80443 | -1.81996 | 1.47385 |
| C | 3.68447 | -1.94295 | -0.51446 |
| C | 4.63128 | -2.54995 | -1.35097 |
| C | 3.90903 | -0.59962 | 0.03598 |
| C | 5.81490 | -1.89326 | -1.64392 |
| H | 4.41419 | -3.53622 | -1.74686 |
| C | 5.19453 | 0.03243 | -0.31821 |
| O | 3.06838 | -0.00228 | 0.74101 |
| C | 6.10795 | -0.60625 | -1.13071 |
| H | 6.54579 | -2.37629 | -2.28571 |
| H | 7.04971 | -0.13703 | -1.39046 |
| O | 5.34186 | 1.25322 | 0.22882 |
| C | 6.53538 | 1.97372 | -0.04797 |
| H | 6.44046 | 2.92111 | 0.48402 |
| H | 7.41868 | 1.43382 | 0.31647 |
| H | 6.64315 | 2.16425 | -1.12329 |
| H | 1.01198 | 0.36763 | -0.09634 |

TS2-c - quartet

| | | | |
|---|---|---|---|
| Fe | -1.45807 | 0.25893 | 0.12797 |
| N | -0.44006 | 1.76004 | 1.04548 |
| N | -1.01409 | -1.00281 | 1.63499 |
| N | -1.80597 | 1.50832 | -1.42347 |
| N | -2.36395 | -1.25350 | -0.83296 |
| C | -0.27485 | 3.05189 | 0.61386 |
| C | -1.35534 | -2.32813 | 1.75267 |
| C | 0.15227 | 1.69388 | 2.28029 |
| C | -0.33169 | -0.68155 | 2.78414 |
| C | -1.44754 | 2.82499 | -1.54419 |
| C | -2.51959 | -2.55131 | -0.41030 |
| C | -2.49819 | 1.19609 | -2.56280 |
| C | -2.97908 | -1.17776 | -2.05590 |
| C | 0.44854 | 3.81994 | 1.59997 |
| C | -0.87673 | -2.85763 | 3.00669 |
| C | 0.71478 | 2.97660 | 2.63401 |
| C | -0.24169 | -1.83585 | 3.64617 |
| C | -1.92289 | 3.36022 | -2.80029 |
| C | -3.25291 | -3.31390 | -1.39347 |
| C | -2.57562 | 2.34896 | -3.43292 |
| C | -3.54002 | -2.46044 | -2.41400 |
| H | 0.71014 | 4.86549 | 1.49682 |
| H | -1.01639 | -3.87854 | 3.33956 |
| H | 1.24165 | 3.18522 | 3.55652 |
| H | 0.24683 | -1.84614 | 4.61246 |
| H | -1.76725 | 4.37802 | -3.13518 |
| H | -3.50625 | -4.36236 | -1.29843 |
| H | -3.06839 | 2.36168 | -4.39693 |
| H | -4.07669 | -2.66350 | -3.33223 |
| C | -0.73976 | 3.55352 | -0.59598 |
| C | -2.05089 | -3.06011 | 0.79503 |
| C | -3.04909 | -0.04414 | -2.85796 |
| C | 0.21130 | 0.56212 | 3.08465 |
| H | -0.53610 | 4.59708 | -0.81644 |
| H | -2.25362 | -4.10478 | 1.01202 |
| H | -3.57316 | -0.14043 | -3.80431 |
| H | 0.72504 | 0.65866 | 4.03651 |
| O | 0.23580 | -0.18234 | -0.82795 |
| S | -3.32296 | 0.99767 | 1.15735 |
| C | -4.33850 | -0.43520 | 1.66428 |
| H | -3.79658 | -1.08485 | 2.35743 |
| H | -5.22303 | -0.03947 | 2.17363 |
| H | -4.65921 | -1.02341 | 0.80025 |
| H | 0.77396 | -1.26998 | -0.56290 |
| C | 1.47770 | -2.32083 | -0.21712 |
| O | 2.55368 | -2.51438 | -1.08864 |
| H | 0.79063 | -3.15628 | -0.36030 |
| H | 1.77238 | -2.14280 | 0.81717 |
| C | 3.70894 | -1.81049 | -1.02875 |
| C | 4.79022 | -2.36951 | -1.69937 |
| C | 3.82475 | -0.51745 | -0.35720 |
| C | 6.02449 | -1.71521 | -1.71344 |
| H | 4.65374 | -3.32273 | -2.19909 |
| C | 5.15566 | 0.10890 | -0.39851 |
| O | 2.86451 | 0.05669 | 0.21290 |
| C | 6.21809 | -0.48462 | -1.06450 |

| | | | |
|---|---|---|---|
| H | 6.86009 | -2.17281 | -2.23478 |
| H | 7.19325 | -0.01300 | -1.09534 |
| O | 5.20264 | 1.28457 | 0.25189 |
| C | 6.42828 | 2.00529 | 0.26346 |
| H | 6.23128 | 2.91474 | 0.83234 |
| H | 7.22318 | 1.42945 | 0.75394 |
| H | 6.74319 | 2.26842 | -0.75406 |
| H | 0.94003 | 0.39710 | -0.46855 |

References


1. Mallinson SJB*, et al.* (2018) A promiscuous cytochrome P450 aromatic O-demethylase for lignin bioconversion. *Nat Commun* 9(1):2487.
2. Evans P (2006) Scaling and assessment of data quality. *Acta Crystallographica Section D* 62(1):72-82.
3. Winter G (2010) xia2: an expert system for macromolecular crystallography data reduction. *Journal of Applied Crystallography* 43(1):186-190.
4. Evans P (2011) An introduction to data reduction: space-group determination, scaling and intensity statistics. *Acta Crystallographica Section D* 67(4):282-292.
5. Padilla JE & Yeates TO (2003) A statistic for local intensity differences: robustness to anisotropy and pseudo-centering and utility for detecting twinning. *Acta Crystallographica Section D* 59(7):1124-1130.
6. Kabsch W (2010) XDS. *Acta Crystallographica Section D: Biological Crystallography* 66(Pt 2):125-132.
7. Emsley P & Cowtan K (2004) Coot: model-building tools for molecular graphics. *Acta Crystallographica Section D* 60(12 Part 1):2126-2132.
8. Adams PD*, et al.* (2010) PHENIX: a comprehensive Python-based system for macromolecular structure solution. *Acta Crystallographica Section D* 66(2):213-221.
9. Terwilliger TC*, et al.* (2008) Iterative model building, structure refinement and density modification with the PHENIX AutoBuild wizard. *Acta Crystallographica Section D* 64(1):61-69.
10. Afonine PV*, et al.* (2012) Towards automated crystallographic structure refinement with phenix.refine. *Acta Crystallographica Section D* 68(4):352-367.
11. Blomfield IC, McClain MS, Princ JA, Calie PJ, & Eisenstein BI (1991) Type 1 fimbriation and fimE mutants of Escherichia coli K-12. *Journal of bacteriology* 173(17):5298-5307.
12. Johnson CW & Beckham GT (2015) Aromatic catabolic pathway selection for optimal production of pyruvate and lactate from lignin. *Metab Eng* 28:240-247.
13. Cadoret F, Soscia C, & Voulhoux R (2014) Gene Transfer: Transformation/Electroporation. *Pseudomonas Methods and Protocols*, eds Filloux A & Ramos J-L (Springer New York, New York, NY), pp 11-15.
14. Johnson M*, et al.* (2008) NCBI BLAST: a better web interface. *Nucleic Acids Research* 36(suppl_2):W5-W9.
15. Katoh K & Standley DM (2013) MAFFT Multiple Sequence Alignment Software Version 7: Improvements in Performance and Usability. *Molecular Biology and Evolution* 30(4):772-780.
16. Cock PJA*, et al.* (2009) Biopython: freely available Python tools for computational molecular biology and bioinformatics. *Bioinformatics* 25(11):1422-1423.
17. Capra JA & Singh M (2007) Predicting functionally important residues from sequence conservation. *Bioinformatics* 23(15):1875-1882.
18. Maier JA*, et al.* (2015) ff14SB: Improving the Accuracy of Protein Side Chain and Backbone Parameters from ff99SB. *J Chem Theory Comput* 11(8):3696-3713.
19. Wang J, Wang W, Kollman PA, & Case DA (2006) Automatic atom type and bond type perception in molecular mechanical calculations. *J Mol Graph Model* 25(2):247-260.
20. Wang J, Wolf RM, Caldwell JW, Kollman PA, & Case DA (2004) Development and testing of a general amber force field. *J Comput Chem* 25(9):1157-1174.
21. Shahrokh K, Orendt A, Yost GS, & Cheatham TE, 3rd (2012) Quantum mechanically derived AMBER-compatible heme parameters for various states of the cytochrome P450 catalytic cycle. *J Comput Chem* 33(2):119-133.
22. Darden T, York D, & Pedersen L (1993) Particle mesh Ewald: An N· log (N) method for Ewald sums in large systems. *The Journal of chemical physics* 98(12):10089-10092.

23. Phillips JC*, et al.* (2005) Scalable molecular dynamics with NAMD. *J Comput Chem* 26(16):1781-1802.
24. Kräutler V, Van Gunsteren WF, & Hünenberger PH (2001) A fast SHAKE algorithm to solve distance constraint equations for small molecules in molecular dynamics simulations. *Journal of computational chemistry* 22(5):501-508.
25. Martyna GJ, Tobias DJ, & Klein ML (1994) Constant pressure molecular dynamics algorithms. *The Journal of Chemical Physics* 101(5):4177-4189.
26. Feller SE, Zhang Y, Pastor RW, & Brooks BR (1995) Constant pressure molecular dynamics simulation: The Langevin piston method. *The Journal of Chemical Physics* 103(11):4613-4621.
27. Kirkwood JG (1935) Statistical mechanics of fluid mixtures. *Journal of Chemical Physics* 3(5):300-313.
28. Kollman P (1993) FREE-ENERGY CALCULATIONS - APPLICATIONS TO CHEMICAL AND BIOCHEMICAL PHENOMENA. *Chemical Reviews* 93(7):2395-2417.
29. Phillips JC*, et al.* (2005) Scalable molecular dynamics with NAMD. *Journal of Computational Chemistry* 26(16):1781-1802.
30. Axelsen PH & Li DH (1998) Improved convergence in dual-topology free energy calculations through use of harmonic restraints. *Journal of Computational Chemistry* 19(11):1278-1283.
31. Deng YQ & Roux B (2009) Computations of Standard Binding Free Energies with Molecular Dynamics Simulations. *Journal of Physical Chemistry B* 113(8):2234-2246.
32. Radak BK*, et al.* (2017) Constant-pH Molecular Dynamics Simulations for Large Biomolecular Systems. *Journal of Chemical Theory and Computation* 13(12):5933-5944.
33. Pohorille A, Jarzynski C, & Chipot C (2010) Good Practices in Free-Energy Calculations. *Journal of Physical Chemistry B* 114(32):10235-10253.
34. Sindhikara DJ, Emerson DJ, & Roitberg AE (2010) Exchange Often and Properly in Replica Exchange Molecular Dynamics. *Journal of Chemical Theory and Computation* 6(9):2804-2808.
35. Steinbrecher T, Joung I, & Case DA (2011) Soft-Core Potentials in Thermodynamic Integration: Comparing One- and Two-Step Transformations. *Journal of Computational Chemistry* 32(15):3253-3263.
36. M. J. Frisch GWT, H. B. Schlegel, G. E. Scuseria, M. A. Robb, J. R. Cheeseman, G. Scalmani, V. Barone, G. A. Petersson, H. Nakatsuji, X. Li, M. Caricato, A. Marenich, J. Bloino, B. G. Janesko, R. Gomperts, B. Mennucci, H. P. Hratchian, J. V. Ortiz, A. F. Izmaylov, J. L. Sonnenberg, D. Williams-Young, F. Ding, F. Lipparini, F. Egidi, J. Goings, B. Peng, A. Petrone, T. Henderson, D. Ranasinghe, V. G. Zakrzewski, J. Gao, N. Rega, G. Zheng, W. Liang, M. Hada, M. Ehara, K. Toyota, R. Fukuda, J. Hasegawa, M. Ishida, T. Nakajima, Y. Honda, O. Kitao, H. Nakai, T. Vreven, K. Throssell, J. A. Montgomery, Jr., J. E. Peralta, F. Ogliaro, M. Bearpark, J. J. Heyd, E. Brothers, K. N. Kudin, V. N. Staroverov, T. Keith, R. Kobayashi, J. Normand, K. Raghavachari, A. Rendell, J. C. Burant, S. S. Iyengar, J. Tomasi, M. Cossi, J. M. Millam, M. Klene, C. Adamo, R. Cammi, J. W. Ochterski, R. L. Martin, K. Morokuma, O. Farkas, J. B. Foresman, and D. J. Fox (2009) Gaussian 09, Revision A. 02. Gaussian. *Inc.: Wallingford, CT*.
37. Becke AD (1988) Density-functional exchange-energy approximation with correct asymptotic behavior. *Physical Review A* 38(6):3098-3100.
38. Becke AD (1993) Density-functional thermochemistry. III. The role of exact exchange. *The Journal of Chemical Physics* 98(7):5648-5652.
39. Lee C, Yang W, & Parr RG (1988) Development of the Colle-Salvetti correlation-energy formula into a functional of the electron density. *Physical Review B* 37(2):785-789.
40. Ribeiro RF, Marenich AV, Cramer CJ, & Truhlar DG (2011) Use of Solution-Phase Vibrational Frequencies in Continuum Models for the Free Energy of Solvation. *The Journal of Physical Chemistry B* 115(49):14556-14562.

41. Zhao Y & Truhlar DG (2008) Computational characterization and modeling of buckyball tweezers: density functional study of concave-convex [small pi][three dots, centered][small pi] interactions. *Physical Chemistry Chemical Physics* 10(19):2813-2818.
42. Grimme S, Ehrlich S, & Goerigk L (2011) Effect of the damping function in dispersion corrected density functional theory. *Journal of Computational Chemistry* 32(7):1456-1465.
43. Grimme S, Antony J, Ehrlich S, & Krieg H (2010) A consistent and accurate ab initio parametrization of density functional dispersion correction (DFT-D) for the 94 elements H-Pu. *The Journal of Chemical Physics* 132(15):154104.
44. Barone V & Cossi M (1998) Quantum Calculation of Molecular Energies and Energy Gradients in Solution by a Conductor Solvent Model. *The Journal of Physical Chemistry A* 102(11):1995-2001.
45. Cossi M, Rega N, Scalmani G, & Barone V (2003) Energies, structures, and electronic properties of molecules in solution with the C-PCM solvation model. *Journal of Computational Chemistry* 24(6):669-681.
46. Schutz CN & Warshel A (2001) What are the dielectric “constants” of proteins and how to validate electrostatic models? *Proteins: Structure, Function, and Bioinformatics* 44(4):400-417.
47. Li L, Li C, Zhang Z, & Alexov E (2013) On the Dielectric “Constant” of Proteins: Smooth Dielectric Function for Macromolecular Modeling and Its Implementation in DelPhi. *Journal of Chemical Theory and Computation* 9(4):2126-2136.
48. Legault C (CYLview, 1.0 b, Université de Sherbrooke, Sherbrooke, Québec, Canada, 2009. *URL http://www. cylview. org (accessed February 1, 2016)*.
49. Lee Y-T, Wilson RF, Rupniewski I, & Goodin DB (2010) P450cam Visits an Open Conformation in the Absence of Substrate. *Biochemistry* 49(16):3412-3419.
50. Sevrioukova IF, Li H, Zhang H, Peterson JA, & Poulos TL (1999) Structure of a cytochrome P450–redox partner electron-transfer complex. *Proceedings of the National Academy of Sciences* 96(5):1863-1868.
51. Haines DC, Tomchick DR, Machius M, & Peterson JA (2001) Pivotal Role of Water in the Mechanism of P450BM-3. *Biochemistry* 40(45):13456-13465.
52. Li H & Poulos TL (1997) The structure of the cytochrome p450BM-3 haem domain complexed with the fatty acid substrate, palmitoleic acid. *Nature Structural Biology* 4(2):140-146.
53. Dubey KD, Wang B, & Shaik S (2016) Molecular Dynamics and QM/MM Calculations Predict the Substrate-Induced Gating of Cytochrome P450 BM3 and the Regio- and Stereoselectivity of Fatty Acid Hydroxylation. *Journal of the American Chemical Society* 138(3):837-845.
54. Follmer AH, Mahomed M, Goodin DB, & Poulos TL (2018) Substrate-Dependent Allosteric Regulation in Cytochrome P450cam (CYP101A1). *Journal of the American Chemical Society* 140(47):16222-16228.
55. Prior JE, Lynch MD, & Gill RT (2010) Broad-host-range vectors for protein expression across gram negative hosts. *Biotechnology and Bioengineering* 106(2):326-332.
56. Tumen-Velasquez M*, et al.* (2018) Accelerating pathway evolution by increasing the gene dosage of chromosomal segments. *Proc Natl Acad Sci U S A* 115(27):7105-7110.
57. Jayakody LN*, et al.* (2018) Thermochemical wastewater valorization via enhanced microbial toxicity tolerance. *Energy & Environmental Science* 11(6):1625-1638.
